# Supplementary material for: Characterization and prediction of individual functional outcome trajectories in schizophrenia spectrum disorders (PREDICTS study): Study protocol
Source: PLoS One. 2023 Sep 21;18(9):e0288354. doi: 10.1371/journal.pone.0288354 (PMC10513234; doi:10.1371/journal.pone.0288354)
Supplement: S1 File — (DOCX) [file pone.0288354.s001.docx]

**STUDY PROTOCOL**

**(Version 5.0, September 29, 2022)**

**Study Title: Characterization and Prediction of Individual Functional Outcome Trajectories in Schizophrenia Spectrum Disorders (PREDICTS Study)**

**Principal Investigator:** George Foussias, MD PhD FRCPC

**Institution:** Centre for Addiction and Mental Health

**Co-Investigators:**  Drs. Andreea Diaconescu, Araba Chintoh, Ariel Graff-Guerrero,

Arun Tiwari, Christopher Bowie, Clement Zai, Colin Hawco, Daniel

Mueller, David Castle, Farooq Naeem, Gary Remington, Ishraq Siddiqui,

James L. Kennedy, Joanna Yu, Mahavir Agarwal, Margaret Hahn, Marta

Maslej, Martin Rotenberg, Michael Kiang, Nicholas Neufeld, Nicole

Kozloff, Ofer Agid, Omair Husain, Peter Selby, Philip Gerretsen, Robert

Zipursky, Sean Hill, Sean Kidd, Vincenzo De Luca, Wei Wang, Yarissa

Herman.

**Source of funding:** CAMH Discovery Fund

**1.0 SPECIFIC AIMS**

Schizophrenia spectrum disorders (SSDs) are associated with significant functional impairments, disability, and low rates of personal recovery, along with tremendous economic costs linked primarily to lost productivity and premature mortality (from natural and unnatural causes). Efforts to delineate the contributors to this disability have highlighted prominent roles for a diverse range of symptoms, physical health conditions, substance use disorders, neurobiological changes, and social factors. These findings have provided valuable advances in knowledge, and helped define broad patterns of illness and outcomes at the diagnostic group level. Not surprisingly, however, there have also been conflicting findings for many of these determinants that reflect the heterogeneous population of individuals with SSDs, and the challenges of conceptualizing and treating SSDs as a unitary categorical construct. As a consequence, at present it is not possible to identify with confidence a particular individual’s functional course in a manner that would enable a personalized approach to treatment intervention that would stand to alter the individual’s functional trajectory and mitigate the ensuing disability they would otherwise experience. To address this ongoing challenge, this study aims to conduct a longitudinal multimodal investigation of a large cohort of individuals with SSDs in order to establish discrete trajectories of personal recovery, disability, and community functioning experienced by individuals with SSDs, and the antecedents and predictors of these trajectories. This will also provide the foundation for the co-design and testing of personalized interventions to alter these functional trajectories and improve outcomes for people with SSDs.

**1.1 Objectives and Hypotheses**

**Objective 1:** Determine the longitudinal functional trajectories of individuals with SSDs across three co-primary domains consisting of personal recovery, disability, and community functioning.

**Hypothesis 1a:** Within each outcome domain, we anticipate approximately 4-5 longitudinal trajectories, consisting of early-sustained improvement, delayed gradual improvement, early improvement / delayed mild-moderate deterioration, early improvement / delayed moderate-severe deterioration, and persistent disability.

**Hypothesis 1b:** We hypothesize that early improvement in personal recovery trajectories will be antecedents of subsequent improvement in disability and community functioning trajectories, while deteriorating or persistent disability, particularly with regards to physical disability, will be early antecedents of worse community functioning.

**Objective 2:** Develop and test predictive models to accurately determine functional trajectories at the person level.

**Hypothesis 2a:** Trajectories of functioning will be differentiable based on sociodemographic and treatment factors, psychopathology, physical health, and biological measures.

**Hypothesis 2b:** A subset of these differential baseline sociodemographic, symptoms, and biological measures will enable the prediction of individual functional trajectories with high positive predictive power.

**Objective 3:** Based on the predictors of longitudinal outcome trajectory subtypes, co-design and pilot targeted real-world interventions developed with patients, family members, and service providers.

**Hypothesis 3:** The co-designed interventions targeting the individual’s longitudinal outcome trajectory will be feasible to implement, satisfactory to participants, and offer preliminary evidence of efficacy for improving longitudinal outcomes compared to treatment as usual.

**Exploratory Objective:** Develop and validate a novel automated method using natural language processing (NLP) and machine learning (ML) to interrogate electronic medical records for the ascertainment of retrospective temporal trajectories in symptom, physical health, treatment, and functional outcomes.

**Exploratory Hypotheses:** NLP-ML-based characterization of temporal trends in functional outcomes, symptoms, physical health, and treatment will be correlated with structured clinical measures of the corresponding constructs, and will differentiate the above prospectively discovered longitudinal outcome trajectory subtypes. Through this, it is anticipated that NLP-ML-based temporal trends in functioning will serve as antecedent markers that inform prospective outcome trajectories.

**2.0 BACKGROUND AND RATIONALE**

**2.1 Schizophrenia spectrum disorders are associated with enduring functional disability**

Schizophrenia spectrum disorders (SSDs) including schizophrenia, schizoaffective disorder, and other primary psychotic disorders, are serious and persistent mental illnesses characterized by a combination of positive symptoms (i.e., delusions and hallucinations), cognitive impairment (including deficits in attention, working memory, and executive function), and negative symptoms (i.e., diminished emotional expression and motivation deficits) [(van Os & Kapur, 2009)](https://www.zotero.org/google-docs/?eSg0tT). Prevalence estimates suggest a lifetime risk of SSDs in the population up to approximately 2% [(Perala et al., 2007)](https://www.zotero.org/google-docs/?tlA5Ch). SSDs are a leading cause of disability worldwide [(Salomon et al., 2015; Whiteford et al., 2015)](https://www.zotero.org/google-docs/?Ib039o), and are associated with tremendous economic and personal costs, with up to 85% of total costs attributed to lost productivity [(Chong et al., 2016; Cloutier et al., 2016; Goeree et al., 2005)](https://www.zotero.org/google-docs/?lo10G7), as well as significant burden on families and caregivers [(Awad & Voruganti, 2008)](https://www.zotero.org/google-docs/?Kuz0UA).

Despite advances in treatment for SSDs and efforts for intensive and early intervention soon after the first psychotic episode, enduring impairments in community functioning continue to be a hallmark of the disorder [(Austin et al., 2013; Schooler, 2006)](https://www.zotero.org/google-docs/?Y2dGC9). In fact, a recent meta-analysis examining recovery rates for individuals with SSDs, operationalized as symptomatic remission combined with a sustained period of adequate functioning, revealed a median recovery rate of 13.5% for individuals with SSDs [(Jaaskelainen et al., 2013)](https://www.zotero.org/google-docs/?kgSIth). Barriers to recovery include impairments in community functioning across multiple domains, including under- or unemployment [(Goeree et al., 2005; Rosenheck et al., 2006)](https://www.zotero.org/google-docs/?Ttykoq), lower rates of educational achievement [(Tempelaar et al., 2017)](https://www.zotero.org/google-docs/?bmak89), and impairments in social and romantic relationships [(Wiersma et al., 2000)](https://www.zotero.org/google-docs/?fL92ls). Compounding these psychosocial contributors to enduring disability are findings that demonstrate significantly elevated rates of physical morbidity leading to early mortality in individuals with SSDs [(M. De Hert et al., 2011; Hennekens et al., 2005; Olfson et al., 2015)](https://www.zotero.org/google-docs/?UPD5oc).

**2.1.1 Individuals with SSDs experience disability across diverse domains of community functioning and quality of life**

Among individuals with SSDs, estimated rates of employment range between 10-30% [(Marwaha & Johnson, 2004; McGurk et al., 2009)](https://www.zotero.org/google-docs/?k3tUIf), yet when people with SSD are asked their views on employment between 55-85% are interested in employment [(McGurk et al., 2009; Westcott et al., 2015)](https://www.zotero.org/google-docs/?VT8cqm). At the population level, employment rates for people with SSD differ between countries due to differences in societal attitudes, levels of stigma, social services, and labour and disability legislation [(Bond & Drake, 2008)](https://www.zotero.org/google-docs/?DoRlYa). There is a dearth of data on employment rates in Canada. One study that included a sample of people with SSD from academic hospitals in Montreal and Halifax found that only 8% received a majority of their income from paid employment [(Caron et al., 2005)](https://www.zotero.org/google-docs/?4clzuu). There is no population-level data available in Canada that estimates employment rates. When employment rates were studied at the population level in Norway, a high-income country with a robust social safety net, only 10% of working-age people with schizophrenia were estimated to be employed [(Evensen et al., 2016)](https://www.zotero.org/google-docs/?IMzWm4). Social and cultural factors are also important to consider at the individual level when looking at both employment and access to services. For example, baseline data from the CATIE study found that only 14% of participants reported competitive employment, however Black participants were approximately half as likely as White participants to report competitive employment [(Rosenheck et al., 2006)](https://www.zotero.org/google-docs/?QiF7Od). In the UK, the AESOP first-episode psychosis cohort found Black Caribbean participants were five times more likely than White British participants to be under- or unemployed [(Morgan et al., 2017)](https://www.zotero.org/google-docs/?ryyq2D). Poor employment outcomes were the norm for most participants, yet at the end of the 10 year follow-up period only 6% of Black Caribbean, 5% of Black African participants, and 14% of White British participants who were initially unemployed at baseline remained employed. In addition, receipt of disability payments has also been linked to reduced competitive employment rates for individuals with SSDs [(Rosenheck et al., 2006)](https://www.zotero.org/google-docs/?eIL0dL).

Although much focus has been given to employment as a functional outcome, there has been increasing study and focus on the importance of educational goals and attainment in people with SSD, with a specific focus on completion of postsecondary education. Independent of the effect education may have on employment, education has value and importance in its own right. Learning may strengthen self-esteem and self-efficacy and increase a person’s sense of purpose which can have a positive downstream impact on mental health [(Hammond, 2004)](https://www.zotero.org/google-docs/?hEEWPz). Although there is likely a bidirectional relationship between education and mental health [(Esch et al., 2014)](https://www.zotero.org/google-docs/?vHIBC9), low educational achievement is often present in people with SSDs from primary school onwards [(Tempelaar et al., 2017)](https://www.zotero.org/google-docs/?ilzlMv), years before the typical onset of symptoms. Considering the average age of onset of SSD is in the teenage and early adult years there may be significant impacts on educational completion and attainment. In line with this, people with SSDs are less likely to have completed secondary and post-secondary education when compared to the general population [(Hakulinen et al., 2019)](https://www.zotero.org/google-docs/?8Pkcym).

Individuals with SSDs also experience impairments across several domains of social functioning. Impairments in social engagement and communication, prosocial activities, and overall interpersonal relationships have been consistently demonstrated across early and later phases of SSDs [(Jean Addington & Addington, 2008; Birchwood et al., 1990; Grant et al., 2001)](https://www.zotero.org/google-docs/?Eqti8W). Longitudinal studies spanning 15 to 20 years have corroborated these findings, demonstrating that up to 77% of individuals with SSDs experience disability in social and romantic relationships, and up to 64% experience social withdrawal [(Eva Velthorst et al., 2017; Wiersma et al., 2000)](https://www.zotero.org/google-docs/?jjEvbi). People with serious mental illnesses, including SSDs, may experience social isolation and limited opportunities to engage in recreational and leisure activities [(Davidson & Stern, 2013)](https://www.zotero.org/google-docs/?UxjxPr). From a psychosocial perspective, there has been less focus on supporting these domains of functioning when compared to vocational, educational and housing. Leisure may be an important focus for people with SSD, particularly if there are significant barriers that exist and limited satisfaction in other domains of functioning and recovery [(Rotenberg & Rudnick, 2017)](https://www.zotero.org/google-docs/?BjhmVX).

In addition to disability that has been described in the context of objective measures of community functioning, substantial evidence has emerged that individuals with SSDs also experience impairments in their subjective perception of their quality of life. Impairments across domains of subjective quality of life, including physical health, subjective feelings, leisure activities, and social relationships have been demonstrated in individuals with SSDs [(Ritsner et al., 2005)](https://www.zotero.org/google-docs/?Fh2UVC). A recent meta-analysis revealed significantly lower quality of life across physical and psychological health, social relationships, and environment among individuals with SSDs compared to healthy control participants [(Dong et al., 2019)](https://www.zotero.org/google-docs/?85ECkf). Several studies by our group have revealed similar overall impairments in subjective well-being and life satisfaction, although with substantial variability such that a sizeable proportion of people with SSDs reported relatively intact subjective well-being, as well as relatively intact subjective happiness across individuals with SSDs [(Agid et al., 2012; Fervaha et al., 2013, 2016; Saperia et al., 2018)](https://www.zotero.org/google-docs/?OY8JMw).

**2.1.2 Individuals with SSDs experience profound physical disability**

Individuals experiencing SSDs have significantly poorer physical health than the general population, including higher rates of cardio-metabolic diseases, respiratory diseases, liver diseases, cancers, and lower physical fitness [(Olfson et al., 2015; Stubbs et al., 2016)](https://www.zotero.org/google-docs/?Z9xW2l). The leading cause of mortality in SSDs is cardiovascular disease (CVD) which reduces lifespan by 15-25 years [(Hennekens et al., 2005)](https://www.zotero.org/google-docs/?Ntjknt), such that the disorder is associated with more loss of lives than cancer or many other physical illnesses [(Saha et al., 2007)](https://www.zotero.org/google-docs/?PzLWfI). SSDs have exceedingly high associated rates of metabolic comorbidity; over half of individuals are obese while the prevalence of type 2 diabetes (T2D) is 3-5 fold higher than the general population [(De Hert et al., 2006b, 2006a; Dixon et al., 2000; Mukherjee et al., 1996)](https://www.zotero.org/google-docs/?rccFq5). Several factors including inherent biological risk, lower self-care, poverty, and increased adverse health behaviors such as smoking, contribute to high rates of obesity and metabolic co-morbidity. Antipsychotic drugs are the cornerstone of treatment in SSDs and widely prescribed across other psychiatric conditions [(Carton et al., 2015)](https://www.zotero.org/google-docs/?8UZXjl). However, their use (across class and individual agents), is associated with severe metabolic adverse effects, including weight gain, dyslipidemia, insulin resistance, and risk of T2D [(Ballon et al., 2014; M. De Hert et al., 2011; Rajkumar et al., 2017)](https://www.zotero.org/google-docs/?4luHAl), all ultimately leading to increased cardiovascular mortality [(Correll et al., 2017)](https://www.zotero.org/google-docs/?UDD7xI).

Despite our field acknowledging the burden of physical health inequalities and cardiovascular mortality in SSDs for over two decades, access to metabolic monitoring, physical health care, and interventions remain suboptimal relative to the general population [(Mitchell et al., 2012; Saxena & Maj, 2017)](https://www.zotero.org/google-docs/?JxMLNB). Concerningly, findings suggest that the mortality gap from CVD in SSDs may be increasing over time relative to the general population [(Oakley et al., 2018; Saha et al., 2007)](https://www.zotero.org/google-docs/?ExPjqB). Recently, a number of international evidence-based recommendations for pharmacological and behavioral interventions have been published to help address these disparities, including a section addressing mental and physical health in the updated Canadian Obesity Guidelines [(Lambert et al., 2017; Moore et al., 2015; Wharton et al., 2020)](https://www.zotero.org/google-docs/?ChcLt2). This represents an important advancement for individuals with SSDs given that beyond cardiovascular health, metabolic comorbidity is also associated with poorer quality of life [(Faulkner et al., 2007)](https://www.zotero.org/google-docs/?IRjsPB), stigma [(Tomiyama et al., 2018)](https://www.zotero.org/google-docs/?qLrBN6), barriers to social engagement [(Young et al., 2017)](https://www.zotero.org/google-docs/?FiQhKO) and poorer adherence with treatment [(Cooper et al., 2016)](https://www.zotero.org/google-docs/?Os2mJp), all contributing to poorer mental health outcomes.

**2.1.3 Personal recovery for individuals with SSDs is a critically important yet largely under-studied longitudinal outcome**

Previous longitudinal studies of functional outcomes in SSD have not focused on subjective recovery as perceived by the person [(Hall et al., 2019; Menezes et al., 2009; Mucci et al., 2021; Velthorst et al., 2017)](https://www.zotero.org/google-docs/?nmHoQe). Beyond measures of function and disability that are often assessed by clinicians and can be conceptualized as objective or clinical forms of recovery, it has become abundantly clear that an individual’s personal experience of recovery is a critically important yet often understudied outcome that recognizes the unique and complex experience of individuals with SSDs. Person-centered or personal recovery can be conceptualized as both a set of processes and/or outcomes that support a personally meaningful life despite having a mental illness [(Slade et al., 2008)](https://www.zotero.org/google-docs/?DPifvD). Personal recovery incorporates key personal factors including connectedness, hope, identity, meaning, and empowerment [(Leamy et al., 2011)](https://www.zotero.org/google-docs/?rBqvtB). Although this conceptualization of personal recovery has been increasingly embraced by stakeholders [(Drake & Whitley, 2014)](https://www.zotero.org/google-docs/?PrBr6p), there has been limited research on longitudinal trajectories of recovery [(Thomas et al., 2017)](https://www.zotero.org/google-docs/?vQjTwM) and gaps in the literature as to how concepts such as community function and disability relate to personal recovery over time [(Van Eck et al., 2018)](https://www.zotero.org/google-docs/?V06IgR).

The relationship between personal recovery and functional outcomes has not been a major focus of study. Previous cross-sectional studies have found an association between recovery and functional outcomes. One study found an association between self-reported recovery (measured by the Mental Health Recovery Measure, MHRM) with the Global Assessment of Functioning (GAF) [(Oliveira-Maia et al., 2016)](https://www.zotero.org/google-docs/?bjr5cf). Another study found recovery (measured by the Recovery Assessment Scale, RAS) to be associated with measures of social support [(Norman et al., 2013)](https://www.zotero.org/google-docs/?eKm6M5). One longitudinal study that assessed recovery (measured using the Questionnaire about the Process of Recovery, QPR) to be associated with disability after nine months (measured using the World Health Organization Disability Assessment Schedule, WHODAS) [(Temesgen et al., 2020)](https://www.zotero.org/google-docs/?1YLKQe). Other studies, however, have not found associations between personal recovery and functional outcomes. For example, across a set of personal recovery measures including the RAS and the MHRM, no association was found with the GAF, Life Skills Profile-16 (LSP-16) nor the Health of the Nation Outcome Scale (HoNOS), which includes items on social functioning [(Andresen et al., 2010)](https://www.zotero.org/google-docs/?LWJmH9). Another longitudinal study that assessed recovery using the QPR and other measures including function using the Personal and Social Performance (PSP) scale or the GAF at baseline and 6-months found that these measures of functioning approached significance as predictors of recovery in a final saturated model [(Heather Law et al., 2016)](https://www.zotero.org/google-docs/?5MYowg). A recent study examining personal recovery among 971 participants with SSDs found that the vast majority of individuals experienced low levels of personal recovery, and moreover, that personal recovery was distinct and only minimally related to objective measures of community functioning [(Best et al., 2020)](https://www.zotero.org/google-docs/?IELnYX). There are also mixed findings for specific components of community functioning such as employment outcomes. Studies have shown higher RAS scores to be associated with employment [(Lloyd et al., 2010; Young et al., 2020)](https://www.zotero.org/google-docs/?aPbuBf), as well as no difference between RAS scores among those employed and not employed [(Connell et al., 2011)](https://www.zotero.org/google-docs/?myLsuk). Many of these studies, however, are limited by their small sample size and cross-sectional or short-term follow-up design.

**2.3 Predictors of functional disability**

**2.3.1 Domains of psychopathology including positive, negative, cognitive, and affective symptoms have been linked to current and enduring disability for individuals with SSDs**

Psychotic symptoms are tremendously disabling [(Salomon et al., 2015; Whiteford et al., 2015)](https://www.zotero.org/google-docs/?PnqQxj) and stand to considerably impair psychosocial functioning. While these symptoms are largely ameliorated by antipsychotic treatment in most individuals, a number of studies have demonstrated relationships between psychotic symptoms and psychosocial functioning even during stable phases of illness, suggesting that residual positive symptoms may influence outcome [(Addington & Addington, 1993, 2000; Alessandrini et al., 2016; Bowie et al., 2008, 2010; Bow-Thomas et al., 1999; Fulford et al., 2013; Goghari et al., 2013; Harrow & Jobe, 2010; Leifker et al., 2009; Pogue-Geile & Harrow, 1984)](https://www.zotero.org/google-docs/?zWp6q4). Positive symptoms also appear to predict aspects of future psychosocial functioning, and have been associated with lower likelihood of meeting criteria for global recovery in later phases of illness [(Goghari et al., 2013; Harrow & Jobe, 2010; Pogue-Geile & Harrow, 1985; Rosen et al., 2011)](https://www.zotero.org/google-docs/?UFg3gF). Further, antipsychotic treatments that largely reduce positive symptom burden (albeit, with the exclusion of individuals who discontinue treatment due to inefficacy or intolerability) are accompanied by only modest improvement in psychosocial functioning [(Swartz et al., 2007)](https://www.zotero.org/google-docs/?c8m9NB). This apparently insinuates that other symptom domains (i.e., those largely unaffected by medications) may bear greater impact on functioning. At best, antipsychotic treatment may be necessary but not sufficient to restore psychosocial functioning [(Swartz et al., 2007)](https://www.zotero.org/google-docs/?Y4uJga). The frequent persistence of poor psychosocial functioning among individuals with SSDs has encouraged the investigation of other symptom domains as potentially more viable targets to improve functional outcome.

Negative symptoms have been more substantially and consistently associated with psychosocial functioning in SSDs. Negative symptoms have generally demonstrated greater association with functioning compared to positive or disorganization symptoms, even when the latter have also been linked to functioning [(Addington & Addington, 1993, 2000; Alessandrini et al., 2016; Brekke et al., 1994; Fulford et al., 2013; Jabben et al., 2010; Möller et al., 2010; Rabinowitz et al., 2012)](https://www.zotero.org/google-docs/?ElnOdb). Cross-sectional investigations have indicated substantial associations between concurrent negative symptom severity and psychosocial functioning or quality of life across illness stages [(Addington & Addington, 2000; Blanchard et al., 2017; Wayne S. Fenton & McGlashan, 1991; Fulford et al., 2013; Kring et al., 2013; Narvaez et al., 2008; Pogue-Geile & Harrow, 1984; Rabinowitz et al., 2012; Van der Does et al., 1993)](https://www.zotero.org/google-docs/?bBPB7v). These relationships, however, may be most prominent during stable illness [(Addington & Addington, 1993; Bow-Thomas et al., 1999)](https://www.zotero.org/google-docs/?8hcZww). Longitudinal investigations of predictors of functional outcome, quality of life, or recovery have consistently indicated that negative symptom severity in the early phase of illness substantially predicts functional impairment in the years to follow [(Ho et al., 1998; Milev et al., 2005; Möller et al., 2010; Ventura, Ered, et al., 2015)](https://www.zotero.org/google-docs/?uMXEUY). Negative symptom severity in first-episode psychosis has similarly been associated with a lower likelihood of recovery five and 10 years later [(Albert et al., 2011; Austin et al., 2013; R. M. G. Norman et al., 2018)](https://www.zotero.org/google-docs/?gXWZON). Negative symptoms measured over the course of chronic illness also appear to predict functioning, quality of life, and recovery at later time points [(Dickerson et al., 1999; Fenton & McGlashan, 1991; Herbener & Harrow, 2004; Novick et al., 2009; Rabinowitz et al., 2012)](https://www.zotero.org/google-docs/?0jF9Rc). Further, relationships with psychosocial functioning are apparent regardless of whether negative symptoms are broadly defined or restricted specifically to primary negative symptoms [(Fervaha et al., 2014; Galderisi et al., 2013)](https://www.zotero.org/google-docs/?hI3kUo). First-episode patients classified as presenting with persistent negative symptoms demonstrate functional impairment [(Hovington et al., 2012; Üçok & Ergül, 2014)](https://www.zotero.org/google-docs/?UlBgTi). Deficit syndrome patients demonstrate greater propensity towards poor psychosocial functioning, long-term disability, and lower longitudinal likelihood of recovery [(Ahmed et al., 2018; Blanchard et al., 2005; Fenton & McGlashan, 1994; Galderisi et al., 2002, 2013; Strauss et al., 2010)](https://www.zotero.org/google-docs/?5ILXfa). With regard to the domains of community functioning, negative symptoms appear to be associated with independent, vocational, and social functioning [(Albert et al., 2011; Alessandrini et al., 2016; Blanchard et al., 2017; Bobes et al., 2009; Fulford et al., 2013; Galderisi et al., 2018; Herbener & Harrow, 2004; Ho et al., 1998; Kring et al., 2013; McGurk et al., 2003; Milev et al., 2005; Norman et al., 2018; Pogue-Geile & Harrow, 1984, 1985; Rosenheck et al., 2006; Shamsi et al., 2011; Ventura et al., 2015)](https://www.zotero.org/google-docs/?BaifJ9). They also appear to be associated with subjective quality of life [(Fujino et al., 2016)](https://www.zotero.org/google-docs/?J2VwlN). Some evidence suggests, however, that the degree of association is strongest with social functioning, somewhat less so with vocational functioning, and weaker still with independent functioning [(Bowie et al., 2006, 2008, 2010; Galderisi et al., 2018; Kring et al., 2013; Leifker et al., 2009; Strassnig et al., 2015)](https://www.zotero.org/google-docs/?E2gMkL).

Cognitive deficits have also been identified as a key determinant of functional outcome. Early investigations had revealed that functional outcome was associated with neurocognition in general and with specific neurocognitive domains, and it was suggested that domain-specific deficits (e.g., in verbal memory and vigilance) may be potential “neurocognitive rate-limiting factors” for adequate functioning (reviewed in [Green, 1996; Green et al., 2000)](https://www.zotero.org/google-docs/?YrcAym). Cognitive impairment was also found to be a predominant predictor, more so than psychopathological symptoms, of overall and specific aspects of functioning in chronically institutionalized elderly individuals with schizophrenia [(Harvey et al., 1998)](https://www.zotero.org/google-docs/?BesXOM) and of independent functioning, particularly in chronically ill inpatients [(Velligan et al., 1997)](https://www.zotero.org/google-docs/?9JzN9f). In contrast, among outpatients, symptoms appeared to be more predictive of community functioning than cognition [(Addington & Addington, 1999, 2000; Norman et al., 1999)](https://www.zotero.org/google-docs/?8KYfTX). Direct associations between functioning (or quality of life) and cognition were limited [(Addington & Addington, 1999, 2000; Dickerson et al., 1999; Reed et al., 2002)](https://www.zotero.org/google-docs/?TYbEF7). Rather, cognition appeared to be more broadly and strongly associated with social skills (or competence), which were in turn associated with functioning or quality of life [(Addington & Addington, 1999, 2000)](https://www.zotero.org/google-docs/?bYfezu). More recently, differences in functioning across older individuals with schizophrenia, categorized by intact versus impaired cognition and symptomatic versus remitted, has suggested cognitive deficits may be a better predictor of functional outcome, especially independent living, than symptoms [(Leung et al., 2008)](https://www.zotero.org/google-docs/?cDJsS6). Other recent work has also highlighted associations between particular domains of cognition and functioning in general [(Cook et al., 2013; Jabben et al., 2010; Milev et al., 2005)](https://www.zotero.org/google-docs/?bkXJaw). Associations have also emerged with the independent, vocational, and social domains of functioning specifically [(Bae et al., 2010; Emre Bora et al., 2006; Greenwood et al., 2005; Kern et al., 2011; Lexén & Bejerholm, 2018; McGurk et al., 2003; Milev et al., 2005; Roncone et al., 2002; Shamsi et al., 2011)](https://www.zotero.org/google-docs/?uoRn94). However, the notion that cognition impacts functioning more substantially than psychopathological symptoms has been challenged by findings of at least equal, often greater, contribution by symptoms [(Jabben et al., 2010; Milev et al., 2005; Mohamed et al., 2008; Narvaez et al., 2008; Villalta-Gil et al., 2006)](https://www.zotero.org/google-docs/?PjkLuB). Further, the independent contribution of cognition typically appears to be modest or nonsignificant in models that also include measures of functional or social competence or skills as predictors of psychosocial functioning [(Bowie et al., 2006, 2008; Leifker et al., 2009)](https://www.zotero.org/google-docs/?HVXEPu). Indeed, cognition’s impact on functioning appears to be largely indirect, mainly through its relationship with functional or social capacity or competence [(Bowie et al., 2006, 2008, 2010; Galderisi et al., 2018; Mucci et al., 2021; Strassnig et al., 2015)](https://www.zotero.org/google-docs/?x5aLbd). Its impact (direct or indirect) appears to be most substantial for independent functioning (or everyday living) followed by vocational functioning, and less so for social functioning [(Bowie et al., 2006, 2008, 2010; Galderisi et al., 2018; Strassnig et al., 2015)](https://www.zotero.org/google-docs/?C3xvfH).

Social cognition has more recently emerged as a potentially important determinant of outcome in schizophrenia, and may uniquely relate to an aspect of psychosocial functioning that is not related to neurocognition [(DeTore et al., 2018)](https://www.zotero.org/google-docs/?MJdQTM). Social cognitive deficits contribute to functional impairment during periods of acute illness and relative remission [(Brekke et al., 2007; Valaparla et al., 2017; Ventura et al., 2015)](https://www.zotero.org/google-docs/?Hj3WYt). With regard to domains of functioning, social cognition may be most closely associated with social functioning [(Shamsi et al., 2011)](https://www.zotero.org/google-docs/?cPCpqY), but also appears to be related to vocational and independent functioning [(Cook et al., 2013; Valaparla et al., 2017; Ventura et al., 2015)](https://www.zotero.org/google-docs/?EQiYCM). Meta-analytic findings suggest that social cognition accounts for more unique variance in functioning than neurocognition [(Fett et al., 2011; Halverson et al., 2019)](https://www.zotero.org/google-docs/?v1F0iL). Theory of mind and social perception and knowledge have emerged as specific cognitive domains that are most strongly associated with psychosocial functioning compared to other neurocognitive and social cognitive domains [(Fett et al., 2011; Halverson et al., 2019)](https://www.zotero.org/google-docs/?0wce6u). Further, the relationship between neurocognition and functioning appears to be at least partially mediated by social cognition [(Addington et al., 2010; Bae et al., 2010; Bhagyavathi et al., 2015; Brekke et al., 2005; Couture et al., 2011; Gard et al., 2009; Halverson et al., 2019; Schmidt et al., 2011; Sergi et al., 2006)](https://www.zotero.org/google-docs/?DyqMRQ). Neurocognition thus appears to largely effect functioning through social cognition.

Beyond traditional domains of psychopathology experienced by individuals with SSDs, i.e., positive, negative and cognitive symptoms, other domains of symptoms have been linked to impairments in community functioning and in particular subjective quality of life. Depressive symptoms have been linked to impairments in social functioning [(Bowie et al., 2006)](https://www.zotero.org/google-docs/?KUI5Vp), and depression and anxiety have been associated with current and longitudinal subjective quality of life, and addition to other psychosocial measures including self-efficacy and social support perceived from significant others [(Ritsner et al., 2012; Saperia et al., 2018)](https://www.zotero.org/google-docs/?oR0Sts). In terms of subjective quality of life, a meta-analysis of the extant literature revealed that while the severity of positive and negative symptoms were linked to subjective quality of life, these relationships are relatively modest, and with substantial heterogeneity across populations studied [(Eack & Newhill, 2007)](https://www.zotero.org/google-docs/?wejuLO). In addition, although cognition has been relatively consistently linked with objective measures of functioning, it has not been found to be related to the subjective quality of life measures among individuals with SSDs [(Tolman & Kurtz, 2012)](https://www.zotero.org/google-docs/?Rrp1j1). Moreover, there have been inconsistent and variable associations between objective measures of functioning and self-rated quality of life for individuals with SSDs [(Nevarez-Flores et al., 2019)](https://www.zotero.org/google-docs/?TqEsXF).

While understudied compared to objective measures of community functioning, recent research has also sought to investigate the factors associated with personal recovery in individuals with SSDs. Here, negative emotions including depression, anxiety, negative self-esteem, and hopelessness, as well as internal locus of control have emerged as central predictors of personal recovery [(Best et al., 2020; Law et al., 2016; Morrison et al., 2013)](https://www.zotero.org/google-docs/?11RSeO). In contrast, positive symptoms, negative symptoms, insight, and neurocognitive impairments, appear to be variably correlated with cross-sectional and longitudinal personal recovery [(Best et al., 2020; Corrigan et al., 2004; Law et al., 2016; Morrison et al., 2013; Norman et al., 2013; Resnick et al., 2004; Roe et al., 2011)](https://www.zotero.org/google-docs/?bc5Ugc)**.**

**2.3.2 Metabolic comorbidity and poor physical health experienced by individuals with SSDs is associated with lower quality of life and disability**

A well-established body of literature reflecting the general population demonstrates that metabolic comorbidity negatively impacts health, psychosocial wellbeing, health-related quality of life measures, and mental health [(Alley & Chang, 2007; Gupta et al., 2015)](https://www.zotero.org/google-docs/?FYj23a). People in the general population living with obesity (which is 2-3 fold more prevalent in individuals with SSDs) also experience pervasive weight bias, stigma and discrimination that further impacts their wellbeing and leads to health and social inequalities [(Sutin et al., 2015)](https://www.zotero.org/google-docs/?EKYH7g). While there is a substantially smaller body of knowledge in this field in individuals with SSDs, findings suggest lower health-related quality of life as compared to the general population [(Foldemo et al., 2014)](https://www.zotero.org/google-docs/?r2I0iM), and similar relationships between obesity, physical health, and cardiovascular (CV) risks factors with poor outcomes.

In keeping with findings in the general population, obese individuals with schizophrenia report poorer general health-related quality of life than those who are not obese [(Strassnig et al., 2003)](https://www.zotero.org/google-docs/?nTeGdC), with independent work from our centre demonstrating that quality of life in SSDs is strongly driven by measures related to body weight [(Faulkner et al., 2007)](https://www.zotero.org/google-docs/?XDjIrL). Having obesity or being overweight in SSDs has also been identified as a barrier to social engagement [(Young et al., 2017)](https://www.zotero.org/google-docs/?hkKTQJ), and has further been associated with poor psychosocial adaptation, low self-esteem [(De Hert et al., 2006b)](https://www.zotero.org/google-docs/?BfCRWH), and excessive personal distress [(Malhotra et al., 2016)](https://www.zotero.org/google-docs/?ilqEbk). Analogous findings have been reported in relation to antipsychotic-induced weight gain, which has been linked to poorer quality of life, reduced well-being and physical vitality [(Allison et al., 2003)](https://www.zotero.org/google-docs/?SKzOyU), and which emerges as the most distressing side effect reported by callers to mental health helplines [(Cooper et al., 2016)](https://www.zotero.org/google-docs/?xIrUmO). Comparable findings have been reported in individuals with co-morbid metabolic syndrome and SSDs, including lower self-ratings of physical health [(Meyer et al., 2005)](https://www.zotero.org/google-docs/?C7lmmB) and psychological health [(Malhotra et al., 2016)](https://www.zotero.org/google-docs/?ZPQgYO). In bipolar disorder, obesity has also been linked to lower levels of functioning, disability and poor health-related quality of life, with parallel findings reported for co-morbid diabetes or insulin resistance, which may also predict a treatment refractory illness course [(Calkin et al., 2015; Kolotkin et al., 2006; Ruzickova et al., 2003)](https://www.zotero.org/google-docs/?h2sV5N). Interestingly, a cohort study of adults living with severe and persistent mental illness demonstrated that self-perceptions of physical health limitations contributed more to reduced life satisfaction and inability to work as compared to psychiatric symptoms [(Dixon et al., 2001)](https://www.zotero.org/google-docs/?M0nYYK).

Additional early work has also examined the presence of metabolic comorbidity in relation to different symptom domains of SSDs, which as discussed in previous sections, are linked to disability and functioning. For example, metabolic syndrome and insulin resistance have been significantly associated with the presence of negative symptoms in SSDs, although causality of this association remains to be established [(Sicras-Mainar et al., 2014; Soontornniyomkij et al., 2019)](https://www.zotero.org/google-docs/?e5y911). A recent meta-analysis of prospective studies examining associations between health risk behaviors and symptoms in bipolar and SSD found that both tobacco use and weight gain/obesity are linked to more severe subsequent psychiatric symptoms and/or decreased level of functioning [(Cerimele & Katon, 2013)](https://www.zotero.org/google-docs/?SFBkPj). Furthermore, as reviewed by our group and highlighted in a recent large meta-analysis [(Bora et al., 2017; MacKenzie et al., 2018)](https://www.zotero.org/google-docs/?CLVeOv), metabolic abnormalities in SSD are increasingly being linked to worse cognitive function. These early findings are in keeping with a growing understanding that obesity, metabolic syndrome and its components are key risk factors for developing cognitive impairment and dementia in the general population [(Biessels et al., 2006; Kullmann et al., 2016; Qiu & Fratiglioni, 2015)](https://www.zotero.org/google-docs/?sDVa4M). To this last point, adiposity, obesity and dysglycemia have been associated with neuro-alterations across numerous brain regions including key areas implicated in cognitive function, which overlap with those altered in SSDs (see section 2.3.3). Thus, the intriguing possibility exists that metabolic comorbidity contributes to brain alterations in SSDs, and may represent an important mediator linking existing brain imaging and cognitive literature to functional outcomes in this population. Furthermore, obesity and other metabolic factors could represent modifiable risk factors for domains such as cognition that are classically recalcitrant to treatment [(Firth, Stubbs, et al., 2017; Gault et al., 2010; Porter et al., 2010)](https://www.zotero.org/google-docs/?D4bw7a).

**Metabolomics**

Metabolomics refers to the large-scale study of metabolites produced by cells, tissues, and organisms, which has high yield potential to identify metabolites that may contribute to, or shift during a disease state highlighting important biochemical illness-related pathways [(Gerszten & Wang, 2008)](https://www.zotero.org/google-docs/?2WUVK8). Not surprisingly, metabolomic approaches have been identified as a powerful tool in understanding the pathophysiology of SSDs [(Nedic Erjavec et al., 2018; Sethi & Brietzke, 2015)](https://www.zotero.org/google-docs/?fDNN46). For example, a recent systematic review of 63 studies identified a number of metabolites associated with SSDs as compared to non-psychiatrically ill controls, including lower levels of essential polyunsaturated fatty acids, vitamin E, and higher levels of lipid peroxidative metabolites and glutamate [(Davison et al., 2018)](https://www.zotero.org/google-docs/?ilJZPK). These metabolites are important in brain development and functioning, protection from cellular damage, energy metabolism, and inflammatory regulation. A recent systematic review has similarly pointed to ten potential metabolite biomarkers for SSDs among others N-acetyl aspartate, lactate, tryptophan, kynurenine, glutamate, creatine, linoleic acid, D-serine, glutathione, and 3-hydroxybutyrate [(Li et al., 2018)](https://www.zotero.org/google-docs/?A0CTEu). A parallel body of literature has suggested that changes in metabolites identified in SSDs represent biomarkers for different components of co-morbid metabolic dysfunction [(Lent-Schochet et al., 2019)](https://www.zotero.org/google-docs/?Myf8fB). Taken together, metabolomics emerges as a novel method with the capacity to bridge metabolism, and metabolic comorbidity in SSD with pathophysiology, leading to identification of novel biomarkers and potential predictors of individual illness trajectories.

**2.3.3 Imaging biomarker-based predictors of functional disability in people with SSDs - structural and functional brain imaging correlates, and potential links to abdominal imaging findings**

There is an emerging brain imaging literature on functional outcomes in patients with SSDs. Historically, gross anatomical findings such as enlarged lateral ventricles have been associated with poor functional outcomes [(Staal et al., 1999)](https://www.zotero.org/google-docs/?loQmHq). Longitudinal magnetic resonance imaging (MRI) has demonstrated volumetric decreases in frontal lobe structures that are also associated with poor functional outcomes [(Ho et al., 2003)](https://www.zotero.org/google-docs/?Ce5qM4). More recent MRI studies have demonstrated more regional findings, particularly with volumetric reductions of the dorsolateral prefrontal cortex (DLPFC, [Prasad et al., 2005)](https://www.zotero.org/google-docs/?kU7Lih) and the inferior, middle, and superior frontal gyri [(Kasparek et al., 2009)](https://www.zotero.org/google-docs/?xbwx8q). Reductions in hippocampal volume also represent amongst the most robust structural findings even early on in illness, and these have been linked to poor cognitive function [(Adriano et al., 2012; Steen et al., 2006)](https://www.zotero.org/google-docs/?tZB1I8). Impaired cognition and negative symptoms are independently associated with poor functional outcomes [(Blanchard et al., 2005; Milev et al., 2005; Rosenheck et al., 2006)](https://www.zotero.org/google-docs/?o9QGkn). Negative symptoms may mediate the relationship between cognition and functional outcomes [(Ventura et al., 2009)](https://www.zotero.org/google-docs/?XsH49s), however recent evidence suggests cognition may mediate the relationship between negative symptoms and functional outcomes [(Eack & Keshavan, 2020; Luther et al., 2020)](https://www.zotero.org/google-docs/?Bvyjn6). Given these complex relationships, multi-modal neuroimaging may be better able to explore gray and white matter relationships with functional outcome, while also probing the network effects of these regional findings.

Network effects may be captured with structural approaches, such as diffusion tensor imaging [(Voineskos et al., 2013)](https://www.zotero.org/google-docs/?pGfBJw). Foundational work by our group at CAMH have demonstrated that the same abnormalities in white matter tracts (inferior longitudinal fasciculus, arcuate fasciculus) impacted in the deficit subtype of schizophrenia and predict functional outcome for schizophrenia patients in general [(Behdinan et al., 2015)](https://www.zotero.org/google-docs/?ZwV828). Network effects may also be captured using functional approaches, such as resting state functional MRI (R-fMRI). For example, cognition in SSDs has been examined with R-fMRI and increased functional connectivity in the mirror neuron and mentalizing systems differentiates patients with poorer functional outcomes from those with better functional outcomes [(Viviano et al., 2018)](https://www.zotero.org/google-docs/?pxDi7R). Interestingly, functional connectivity alone is sufficient to distinguish poor from normal cognitive performers and associated functional outcomes. More recently, decreased functional connectivity between the globus pallidus and prefrontal cortex (including the DLPFC) was associated with functional outcomes [(Tarcijonas et al., 2020)](https://www.zotero.org/google-docs/?y8sxwh).

Intriguingly, a parallel line of evidence from the field of obesity and diabetes research has replicated associations between measures of adiposity, obesity, glycemia and brain structure across thousands of individuals, including neuro alterations that overlap with those associated with SSDs [(Dekkers et al., 2019; García-García et al., 2019; Janowitz et al., 2015; Ronan et al., 2016; Willette & Kapogiannis, 2015)](https://www.zotero.org/google-docs/?JiOwQz). Early findings in individuals with schizophrenia and bipolar disorder have demonstrated that alterations in brain volume and structural connectivity may be linked with metabolic comorbidity, which has been hypothesized to contribute to these disruptions [(Hajek et al., 2014; Kolenic et al., 2018; Spangaro et al., 2018)](https://www.zotero.org/google-docs/?RmyCha). In turn, the recent advent of MRI-based body fat quantification creates an opportunity to investigate relationships between brain and physical health. Mounting evidence suggests the regional distribution of body fat is the critical correlate of metabolic abnormalities; and visceral fat (the intra-abdominal fat surrounding organs in undesired sites such as the liver), is an independent predictor of metabolic abnormalities, and future CV risk [(Boyko et al., 2000; Kuk et al., 2006)](https://www.zotero.org/google-docs/?arg8dW). While routine clinical measurements, such as waist circumference, allow for a crude estimation of visceral fat, they cannot distinguish subcutaneous fat from visceral fat [(Emerging Risk Factors Collaboration et al., 2011)](https://www.zotero.org/google-docs/?QcJxuW). In contrast, MRI is considered the gold standard for measuring body fat composition [(Seidell et al., 1990; Shuster et al., 2012)](https://www.zotero.org/google-docs/?5NJZkR). Our present capacity to concomitantly perform abdominal and brain MRI scans can provide a novel approach to capture contributory effects of metabolic comorbidity to brain structural alterations and functional outcomes.

**2.3.4 Genetics, genome-wide association studies (GWASs) and polygenic risk scores - opportunities for augmenting prediction of outcomes for people with SSDs**

Twin, family, and adoption studies have supported a strong genetic component in the risk of schizophrenia, with heritability estimates in the range of 80% [(Sullivan et al., 2003)](https://www.zotero.org/google-docs/?P9Fe5y). Linkage studies have identified a number of early candidate genes, including Catehcol-o-Methyltransferase *COMT* at 22q11 [(Gothelf et al., 2005; Shifman et al., 2002)](https://www.zotero.org/google-docs/?8J7Jon), Disrupted in Schizophrenia-1 *DISC1* at 1q42 [(Hennah et al., 2005)](https://www.zotero.org/google-docs/?VpcMS2), Dysbindin-1 *DTNBP1* at 6p24-22 [(Talbot et al., 2004)](https://www.zotero.org/google-docs/?29jBzH), Neuregulin-1 *NRG1* at 8p12-21[(T. Li et al., 2004; Zhao et al., 2004)](https://www.zotero.org/google-docs/?UQUFmp), *G72* and *DAAO* at 13q32-34 [(Chumakov et al., 2002)](https://www.zotero.org/google-docs/?NLlspA). However, these genes were not among the top findings in genome-wide association studies of schizophrenia (SCZ) in over 30,000 patients and over 100,000 healthy controls , where 108 genetic loci were found to be significantly associated with schizophrenia, with the HLA region (containing many immune response genes) being the top hit [(Schizophrenia Working Group of the Psychiatric Genomics Consortium, 2014)](https://www.zotero.org/google-docs/?IorOgz). The major component of the HLA signal was found to be explained by the Complement Component *C4* gene [(Sekar et al., 2016)](https://www.zotero.org/google-docs/?3se7Cx). Other findings from GWAS included *TCF4* [(Stefansson et al., 2009)](https://www.zotero.org/google-docs/?ovFXpc), *ZNF804A* [(O’Donovan et al., 2008)](https://www.zotero.org/google-docs/?fXpTLz), *CACNA1C*, and the prime schizophrenia candidate gene, dopamine D2 receptor *DRD2* [(Schizophrenia Working Group of the Psychiatric Genomics Consortium, 2014)](https://www.zotero.org/google-docs/?fyvUBU). As the sample of the PGC-Schizophrenia GWAS continues to grow (currently at over 60,000 patients), additional significant common genetic variants will continue to emerge [(Ripke et al., 2020)](https://www.zotero.org/google-docs/?J4lLSq).

Identification of genome-wide significant variants marks significant progress in understanding the genetic architecture and pathoetiology of SCZ. However, the clinical utility of individual common variants in diagnosing SCZ is minimal. To this end, genome-wide polygenic risk scores (PRS) have emerged as an important tool to potentially identify psychiatric patients at clinically significant risk of diseases, lack of response and poor prognosis among others. PRS is an approach wherein the effect of genome-wide variations is aggregated and can be used to investigate their combined predictive power in a new validation sample of the same disease or to identify genetic overlap with different traits/disorders. PRS is typically calculated as a weighted sum of the risk alleles exceeding a certain p-value threshold, weighted by their association coefficients identified typically from a genome-wide association study (GWAS) [(Euesden et al., 2015)](https://www.zotero.org/google-docs/?PiZ6G2). This technique was first employed in the SCZ GWAS and showed that the risk of SCZ is polygenic and overlaps with BD but not with several non-psychiatric diseases [(International Schizophrenia Consortium et al., 2009)](https://www.zotero.org/google-docs/?Dly7CL). Since then PRS has been used to establish genetic overlap between several diseases including SCZ and BD, MDD and BD [(Bulik-Sullivan et al., 2015; McCoy et al., 2017)](https://www.zotero.org/google-docs/?NthbNh). Similarly, PRS from SCZ has been correlated with severity of psychiatric symptoms, antipsychotic response, prefrontal activity, IQ, educational attainment, brain structures and reduction in cortical volumes among others [(Weinberger, 2019)](https://www.zotero.org/google-docs/?4CdADd). In addition, PRS can be used to estimate the risk for cardiovascular and metabolic disorders as well as for alcohol and cannabis use. Integrating PRS information with clinical and demographic factors can lead to better treatment of psychiatric disorders. A major hindrance in the usage of PRS for genetic risk prediction has been insufficiently powered GWAS and the lack of large diverse datasets to validate and test these scores. However, over the past few years, GWAS with large sample sizes have been conducted and identified several genetic loci associated with SCZ, BD and MDD. Studies with such large sample sizes provide more precise effect size estimates that can be potentially utilized for clinically useful risk stratification. Another limitation of PRS is the limited predictive ability due to allele frequency, linkage disequilibrium differences across ancestries [(Martin et al., 2019)](https://www.zotero.org/google-docs/?G0UFAG). However, recently large GWAS for SCZ, have been conducted in individuals of Asian, African and Latin ancestry improving the possibility of using PRS score for risk prediction across ancestries.

With regards to the effects of genetic variants on functional outcomes and disability for people with SSDs, a recent genetic study in a sample of schizophreniform patients reported that polygenic risk scores were negatively correlated with Global Assessment of Functioning (GAF) scale scores pre-treatment [(Santoro et al., 2018)](https://www.zotero.org/google-docs/?yJRlCp). Another study demonstrated that PRS-SCZ explained a significant, albeit small, proportion of variance in quality of life in people with SSDs above and beyond demographic and clinical variables [(Pazoki et al., 2020)](https://www.zotero.org/google-docs/?js3IMs). While there is currently a paucity of studies directly examining genetic associations of functioning outcomes, additional genetic findings in SSDs examining symptom domains that are predictors of functional outcomes may offer additional insights. There has been substantial focus in the genetic context on treatment-resistant schizophrenia that affects approximately 30% of patients [(Meltzer, 1997)](https://www.zotero.org/google-docs/?jfFA6B). The majority of genetic studies have focused on antipsychotic treatment response, including studies of clozapine response that have sometimes been interpreted together with studies of treatment resistant schizophrenia [(Vita et al., 2019; Nucifora et al., 2019)](https://www.zotero.org/google-docs/?HRNTeg). The preliminary findings of a combined effect of the *SLC6A4* HTTLPR and *SLC6A3* 3’-untranslated region variable-number tandem repeat polymorphisms require independent replications [(Bilic et al., 2014)](https://www.zotero.org/google-docs/?GQ5U4m), and most candidate gene studies have not yielded significant replicated findings [(Teo et al., 2012)](https://www.zotero.org/google-docs/?qctnHp). Studies analyzing polygenic risk scores for schizophrenia (PRS-SCZ) with treatment resistance have also yielded mixed findings. Individuals with treatment-resistant schizophrenia have been found to have higher PRS-SCZ than non-treatment resistant populations in some studies [(Frank et al., 2015; Gasse et al., 2019; Werner et al., 2020)](https://www.zotero.org/google-docs/?ClGBBt) but not in others [(Kowalec et al., 2019; Martin & Mowry, 2016)](https://www.zotero.org/google-docs/?oKB5Nz). While these studies have looked at associations with either diagnosis-level or a priori defined subtypes of SSDs, a recent study offers some parallels to the present proposed work. In this study by Habtewold and colleagues [(Habtewold et al., 2020)](https://www.zotero.org/google-docs/?HCDK96), PRS-SCZ was investigated with respect to its relationship with distinct data-driven trajectories of cognitive functioning in individuals with SSDs over the course of three years, revealing that PRS-SCZ significantly predicted risk of experiencing poor cognitive trajectories. Similarly, transition to psychosis in clinically high risk individuals could be predicted by sequentially combining PRS with clinical and biological variables [(Koutsouleris et al., 2020)](https://www.zotero.org/google-docs/?0ZWXAS). Overall, these findings and the evolving field of PRS analyses suggest that combining genetic information with other clinical and biological risk factors may enable enhanced prediction of outcomes and support clinical decision-making for people with SSDs and other mental health disorders.

**2.3.5 Socio-environmental factors have been linked to recovery and functional disability outcomes in people with SSDs**

The role of socio-environmental exposures at both the individual and environmental levels has been well established as risk factors in developing a SSD and the extension of these known risk factors on functioning, disability and recovery is an area of increasing study.

Some of the most compelling evidence for socio-environmental risk comes from well-replicated findings of increased incidence of SSD in immigrant and minority ethnic communities [(Anderson et al., 2015; Kirkbride et al., 2010; Morgan et al., 2017; Selten et al., 2020)](https://www.zotero.org/google-docs/?IdNpS1). Urbanicity has also been a widely studied risk factor, with earlier studies finding people born and raised in some urban environments being at higher risk of developing SSD [(Vassos et al., 2012)](https://www.zotero.org/google-docs/?2sM5jO), with more recent studies finding mixed results [(Fett et al., 2019; Jongsma et al., 2018)](https://www.zotero.org/google-docs/?z31MLP). Heterogeneous findings in relation to urbanicity are likely suggestive of differential exposure to other factors, including other socio-environmental risk factors like deprivation and marginalization [(O’Donoghue et al., 2016)](https://www.zotero.org/google-docs/?XIJXbI), biological risk factors including substance use [(Forti et al., 2019)](https://www.zotero.org/google-docs/?Y5qRoX) as well protective factors which may include ethnic density (which describes living in areas with people of a similar ethnocultural background) that may help buffer and reduce stress [(Schofield et al., 2017)](https://www.zotero.org/google-docs/?3aKq12).

Other social factors have also been associated with risk and course of SSD, including childhood trauma [(Vassos et al., 2012)](https://www.zotero.org/google-docs/?zqsoQE), discrimination [(Oh et al., 2014)](https://www.zotero.org/google-docs/?jqlygm), social isolation and loneliness [(Michalska da Rocha et al., 2018)](https://www.zotero.org/google-docs/?uCaZYS), as well as employment and achievement-expectation mismatch [(Reininghaus et al., 2008)](https://www.zotero.org/google-docs/?KMA40j). In contrast to these risk factors, psychological resilience [(Kim et al., 2013)](https://www.zotero.org/google-docs/?51WxSE) and social capital [(Rotenberg et al., 2020)](https://www.zotero.org/google-docs/?PPNxhf) have also been identified as potential protective factors that may buffer the impact of detrimental socio-environmental exposures and support positive outcomes. Beyond the ongoing impact these risk factors have on the course of SSD and outcomes, people with a SSD must also contend with the impacts of stigma, social exclusion and challenges in accessing services and care.

Although many socio-environmental factors have been studied in relation to the risk of developing a SSD, in general there has been much less focus on how these factors extend to outcomes and recovery with rigorous methods. A number of previous studies have attempted to examine outcomes and functional trajectories in diverse samples of people with SSD but have been limited by study quality and sample size [(Chorlton et al., 2012)](https://www.zotero.org/google-docs/?CJtoVw). One of the better designed studies that has examined this issue is the UK AESOP-10 cohort which included an ethnically diverse cohort of people who initially presented with a first-episode psychosis. At the 10-year follow-up point, Black Caribbean patients experienced worse clinical, social and service use outcomes with some evidence that baseline social disadvantage contributed to these disparities [(Morgan et al., 2017)](https://www.zotero.org/google-docs/?r8QHZ9).

These findings highlight how social exclusion and discrimination perpetuate poor outcomes. Stigma, which can lead to further social exclusion and discrimination associated with a diagnosis of SSD further compounds and drive negative outcomes. Internalized stigma is negatively associated with social functioning [(Lysaker et al., 2007)](https://www.zotero.org/google-docs/?en7t2U) and recovery-related outcomes [(Yanos et al., 2008)](https://www.zotero.org/google-docs/?pTeQEq). As a process, recovery has been positively associated with social support and a sense of belonging and has been negatively associated with isolation, negative interactions and feelings of hopelessness [(Soundy et al., 2015)](https://www.zotero.org/google-docs/?ziO44Z). Life events and trauma impact how people view themselves and the world around them [(Gluhoski & Wortman, 1996; Pearlman, 1997)](https://www.zotero.org/google-docs/?gbr2Ol). Trauma is a known risk factor in developing SSD [(Schäfer & Fisher, 2011)](https://www.zotero.org/google-docs/?8Roeam) and also extends to how people function and recover following a diagnosis of SSD. Childhood trauma has been associated with poorer social functioning in people with SSD [(Alameda et al., 2015; Nierop et al., 2016)](https://www.zotero.org/google-docs/?IsyVL3), with earlier exposure to trauma being associated with worse outcomes [(Alameda et al., 2015)](https://www.zotero.org/google-docs/?frAetD). Other studies have found specific forms of childhood abuse and neglect, such as physical neglect, may have a greater effect on later adult functioning in people with a SSD [(Gil et al., 2009)](https://www.zotero.org/google-docs/?C0hQd0).

Further longitudinal work in a diverse clinical population with SSD is needed to build on the extant literature to further examine how socio-environmental factors in conjunction with biological factors impact functioning, disability and recovery over time. The proposed study is uniquely positioned to be able to identify specific socio-environmental risk factors, timing of exposures and how these factors interact with biological and genetic factors that are also areas of focus. It is possible that the study may identify trajectories where outcomes are predominantly driven by biological factors (i.e., genetic and neurodevelopmental), trajectories that are primarily driven by socio-environmental factors (i.e., trauma, adverse life events), and trajectories driven by a confluence of both socio-environmental and biological factors. Given the longitudinal nature of this study there is also opportunity to identify points across all trajectories (regardless of the impact of biological or socio-environmental risk factors) where socio-environmental factors may exert a larger degree of influence which may help inform future interventions to improve functioning and recovery.

**2.4 Outcomes for individuals with SSDs are heterogeneous, with distinct trajectories of functioning within population subsets**

With the longstanding recognition of heterogeneity in outcomes for individuals with SSDs, there have been several efforts to delineate course types in SSDs over the past several decades. Early work examining course typology across longitudinal studies confirmed clinical observations of heterogeneous trajectories of both symptoms and functioning over time, with the identification of up to 8 different course trajectories that varied based on type of onset (i.e., acute or chronic), course type (i.e., simple vs. undulating), and end state (i.e., recovery/mild impairment vs moderate/severe impairment) [(Carpenter & Kirkpatrick, 1988; Harding, 1988)](https://www.zotero.org/google-docs/?x6ACPe). Additional efforts have involved a priori subtyping of individuals based on established diagnostic subtypes, where for example individuals meeting criteria for the deficit syndrome (i.e., individuals with SSDs who experience enduring primary negative symptoms) have been shown to have a different longitudinal functional course characterized by enduring disability and fewer periods of adequate community functioning compared to individuals without the deficit syndrome [(Strauss et al., 2010)](https://www.zotero.org/google-docs/?lZYreo).

More recently, investigations have utilized data-driven modeling approaches to interrogate longitudinal trajectories of functional outcomes in people with SSDs, though with most studies focused on the first few years of illness. [Hodgekins et al., (2015)](https://www.zotero.org/google-docs/?R3hRly) examined trajectories of social functioning in the first year following a first episode psychosis, revealing three trajectories of social functioning. These trajectories were characterized by: 1) high initial functioning with gradual deterioration (high-decreasing); 2) moderate initial disability that gradually improved over time (moderate-increasing); and 3) persistent disability over time (low stable). Compared to individuals with persistent disability, those in higher social functioning trajectories were more likely to be female and have better premorbid functioning in late adolescence. Further, individuals in the moderate-increasing trajectory had less positive symptoms at baseline, and were less likely to be from an ethnic minority group, while those in the high-decreasing trajectory were also more likely to have an older age of onset, and lower baseline negative symptoms. Another study also examining short-term functional outcome trajectories within the first year of illness and treatment initiation confirmed the existence of multiple trajectories, revealing a four-cluster trajectory with subsets of individuals demonstrating substantial early or delayed improvements in functioning, while other individuals experienced either middle or poor functional courses [(Hall et al., 2019)](https://www.zotero.org/google-docs/?M1X2Oc). Interestingly, the delayed improvement and poor outcome trajectories were indistinguishable at baseline, with both subgroups exhibiting severe positive and negative symptoms, although with very different longitudinal functioning pathways such that individuals in the delayed improvement subgroup demonstrated equivalent functioning to the good outcome trajectory at one year. This delayed improvement subtype, however, was composed of more female and Caucasian participants, with higher cognitive functioning, better premorbid adjustment, having less substance use history, and from higher socioeconomic backgrounds, characteristics shared with the good outcome trajectory subtype.

Longer-term longitudinal studies have served to confirm these early outcome trajectories. In a three-year follow-up study of individuals after a first episode of psychosis, [Chang et al., (2018)](https://www.zotero.org/google-docs/?omLmB7) identified four outcome trajectories based on the SOFAS, comprising early improvement, gradual improvement, early improvement with later deterioration, and persistently poor outcome subtypes. Individuals across these four trajectories were differentiable based on a higher preponderance of females among better outcome trajectories, higher education attainment in the better outcome trajectories, and a lower frequency of hospitalization at first hospitalization among those demonstrating early improvement. Psychopathology was not predictive of functional outcome trajectories with only a trend related to the severity of negative symptoms at baseline. Finally, data from the Suffolk County Mental Health Project has been used to evaluate 20-year trajectories of social functioning for individuals with SSDs following their first admission, albeit more coarse-grained due to large gaps of 6 and 10 years between assessments in the later stages of follow-up. Initial comparisons of longitudinal functioning for individuals with SSDs compared to those with mood disorders with psychosis and other psychotic disorders described a substantial and consistent decline in functioning over time for people with SSDs, although with notable heterogeneity in levels of functioning between individuals in the same diagnostic group [(Kotov et al., 2017)](https://www.zotero.org/google-docs/?k8L8Mg). Subsequent data-driven examination of social functioning trajectories across these disorders revealed four trajectory subtypes consisting of preserved, moderately impaired, severely impaired, and profoundly impaired functioning [(Velthorst et al., 2017)](https://www.zotero.org/google-docs/?AZq3mv). These trajectories were differentiable even early on based on premorbid adjustment prior to illness onset, sociodemographic characteristics including male sex and ethnicity, and over the course of follow-up assessments differed in the severity of negative symptoms and antipsychotic use, and to a lesser extent severity of positive symptoms.

Overall, these studies provide more nuanced understanding of the heterogeneous longitudinal functional trajectories for individuals with SSDs compared to simple evaluations at a diagnostic group level. The three to four distinct trajectories identified across these studies reveal different extent and timing of functional improvements for individuals with SSDs, with important sociodemographic and clinical correlates that differentiate these course subtypes. Notably, however, these studies have relied on narrow measures of outcomes focused on biomedical frameworks of community functioning rather than personal recovery or self-reported disability, with a limited set of sociodemographic and clinical variables, and without concurrent examination of physical health, neurobiological markers or genetic correlates that may serve to enhance the early identification and prediction of outcome trajectories at the individual patient level.

**2.5 Electronic Medical Record (EMR) - based analytics enable comprehensive phenotyping and longitudinal characterization of clinical outcomes**

EMRs capture a large amount of longitudinal clinical data recorded over the course of an individual’s lifetime. This includes administrative data (e.g., sociodemographic information and treatment encounters), structured clinical data (e.g., prescribed medications, diagnoses, laboratory data, and physical health measurements), and unstructured clinical data (e.g., consultation notes, progress notes, admission and discharge notes), that collectively capture individuals’ rich longitudinal histories and illness course. Natural language processing (NLP) and machine learning (ML) methods are increasingly used to extract this retrospective longitudinal clinical information from free text within EMRs with an efficiency and scale not achievable by manual review of EMRs. Such methods have been successfully utilized at CAMH, for example, to evaluate free text EMR clinical notes for ascertainment of depression diagnoses in youth in the absence of structured diagnostic codes [(Geraci et al., 2017)](https://www.zotero.org/google-docs/?c0sV4M). More broadly, these methods have enabled improved accuracy of case detection across multiple medical conditions compared to structured diagnostic codes [(Ford et al., 2016; Zhou et al., 2015)](https://www.zotero.org/google-docs/?c9T58V), with such phenotyping also enabling clinical prediction of future disease [(Miotto et al., 2016)](https://www.zotero.org/google-docs/?H3zLjx).

Such approaches have also enabled the extraction of structured depression severity ratings on the Patient Health Questionnaire-9 (PHQ-9) from free text clinical notes, demonstrating high sensitivity and specificity, and the ability to identify individuals meeting severity ratings indicative of the presence of depression where such a structured diagnosis was missing from the medical record [(Adekkanattu et al., 2018)](https://www.zotero.org/google-docs/?vXlEwG). This has also enabled the evaluation of other clinical phenomena including mood instability and relationships with clinical outcomes [(Patel, Lloyd, et al., 2015)](https://www.zotero.org/google-docs/?4Xvv7d). For individuals with SSDs, structured EMR-based data also enables retrospective evaluation of the longitudinal evolution of symptoms, treatment trajectory, and outcomes [(Fusar-Poli et al., 2020)](https://www.zotero.org/google-docs/?SZh7fc), as well as evaluations of the impact of structural treatment interventions on health outcomes [(Barkhuizen et al., 2020)](https://www.zotero.org/google-docs/?NOszYq). In addition, NLP-ML methods have enabled the identification of negative symptom burden from free text EMR clinical notes in a large sample of patients with SSDs receiving mental health care in the UK, and its association with clinical characteristics and outcomes [(Patel, Jayatilleke, et al., 2015)](https://www.zotero.org/google-docs/?6cs8K1). A similar approach, again using free text EMR clinical notes was utilized to evaluate the prevalence of adverse drug events including extrapyramidal symptoms in individuals with severe mental illnesses [(Iqbal et al., 2015)](https://www.zotero.org/google-docs/?keRQHo).

EMR-based NLP-ML approaches have also been utilized to investigate and characterize illness trajectories and uncover homogeneous phenotypes amidst heterogeneous clinical presentations. A recent study, for example, utilized this approach to examine illness trajectories and end-of-life care for individuals with dementia in the final two years of life [(Wang et al., 2018)](https://www.zotero.org/google-docs/?RpKjx6). Topic modeling using NLP and ML algorithms was able to identify deteriorating trajectories of mental and physical health, physical functional status, and corresponding trajectories for discrete events such as falls and fracture, elements of end-of-life care, as well as family, social, and spiritual supports. Further, such an approach has been utilized to identify predictors of mortality across different time frames that can help inform early palliative care decision-making [(Wang et al., 2019)](https://www.zotero.org/google-docs/?lvRQ9A). Similar approaches have been used to dissect clinical heterogeneity in the presentation and outcomes for sepsis [(Fohner et al., 2019)](https://www.zotero.org/google-docs/?9w10vO), enabling the identification of distinct clinical phenotypes that were correlated with differential immune response biomarkers and clinical outcomes [(Seymour et al., 2019)](https://www.zotero.org/google-docs/?9mx5Vf).

**2.6 Summary and Rationale**

Despite many decades of research and efforts at treatment innovation, at the diagnostic group level people with SSDs continue to experience significant impairments across important outcome domains including personal recovery, physical and mental health related disability, and community functioning. To date, despite the longstanding recognition of heterogeneity in the courses and outcomes for people with SSDs, investigations for the most part have utilized population-based approaches to identify a broad range of demographic, social, environmental, clinical, physical health, and biological predictors of functional outcomes. This approach has relied on the assumption of uniform effects of these predictors across all individuals with SSDs, i.e., that their outcomes and the predictors of these outcomes are homogeneous. Contradictory findings across studies, however, suggest that such effects are not in fact uniform, but rather depend on the population sampled and the underlying trajectories comprising the sampled population. Similar challenges have emerged with regards to inconsistent findings for treatment interventions that have either focused on treating a specific domain of psychopathology as an effector of subsequent improvements in functioning, or broader health and psychosocial interventions that are delivered to individuals based on their diagnosis of an SSD, without precise consideration of individual heterogeneity that may result in similar longitudinal functional disability arising through different underlying mechanisms. Recent data-driven efforts to dissect this heterogeneity have revealed the existence of distinct underlying outcome trajectories among populations of people with SSDs, that are at times indistinguishable based on presenting clinical characteristics but that have very different longitudinal courses. While this has reinforced historic conceptualizations of the distinct course types experienced by people with SSDs, these investigations have focused on narrow measures of functioning, have either been focused primarily on short-term outcomes in the early years of illness or coarsely examined outcomes over longer periods, and have lacked more comprehensive examinations of physical health and biological characteristics that may enhance prediction and delineation of these differential outcome trajectories and the person level.

Priorities regarding what constitutes recovery and relevant functional outcomes in SSDs vary considerably between patients and care providers within a biomedical framework. Traditional objective measures of functioning and recovery from a biomedical perspective typically focus on symptom management and indices of social and role functioning [(Jaaskelainen et al., 2013; Robinson et al., 2004)](https://www.zotero.org/google-docs/?XOUz6z). Patient perspectives, however, typically prioritize areas related to quality of life, self-esteem, connectedness, meaning and purpose, hope and optimism, and empowerment within the construct of personal recovery [(Law & Morrison, 2014; Leamy et al., 2011)](https://www.zotero.org/google-docs/?DC7gQV). There is currently limited research, however, evaluating the longitudinal trajectories of personal recovery and disability in people with SSDs, and how these outcomes interact with each other and with traditional measures of community functioning over time.

In order to advance the understanding of outcome trajectories and potential treatment intervention opportunities to alter these outcomes for individuals with SSDs, there is a need for a broader and more comprehensive evaluation of discrete trajectories of personal recovery, disability, and community functioning. This will advance understanding of their unique and shared longitudinal courses, as well as their underlying clinical, socio-environmental, physical health and biological predictors. No longitudinal study to date, to our knowledge, has examined these concurrent trajectories in individuals with SSDs, nor incorporated physical health, neurobiology, and genetic predictors of these trajectories. The inclusion of EMR-based analytics and provincial administrative health data proposed in this study affords additional unique opportunities for expanded clinical phenotyping and longitudinal outcome evaluations. The proposed study also sets the stage for the co-design and testing of real-world interventions targeting discrete outcome trajectory subtypes through a precision medicine approach in order to improve the individual’s outcome trajectory and promote recovery and reduce the enduring disability they experience.

**3.0 METHODOLOGICAL DETAILS**

This study will utilize a longitudinal sequential cohort design, with broad sampling across age ranges and phases of illness for individuals with SSDs. The characterization and prediction of trajectories of functional disability will provide the foundation for integration of interventions targeting specific predictors of functional disability that will be co-designed with patients, family members, and service providers, and evaluated as embedded trials within this cohort study. Selection of outcomes measures and assessments including the comprehensive measurement of relevant domains of functional outcome and recovery, was informed by guidance from patient and family member representatives of the study steering committee, their integration within routine clinical services at CAMH, and their alignment with other large clinical research initiatives currently taking place both locally and internationally. Study assessments will be completed at baseline, and longitudinal as outlined in Appendix 1. Extended follow-up beyond the initial three years will be pursued through external funding to support this work.

**3.1 Participants**

Participants will be eligible for this study if they: 1) are 16 years of age and older; 2) have a DSM-5 diagnosis of any schizophrenia spectrum disorder (SSD; e.g., schizophrenia, schizoaffective disorder, schizophreniform disorder, brief psychotic disorder, delusional disorder, unspecified schizophrenia spectrum or other psychotic disorder, other specified schizophrenia spectrum and other psychotic disorder, substance/medication-induced psychotic disorder), or related psychotic disorders (including bipolar I disorder with psychotic features or major depressive disorder with psychotic features); and 3) have adequate fluency in English to participate in clinical care without the need for a translator . Of note, participants will be eligible regardless of duration of illness, co-morbid mental or physical health conditions, capacity to consent to treatment, or whether they are receiving inpatient or outpatient services at CAMH. Participants will provide written informed consent, and for those individuals deemed incapable to consent to treatment by their treatment team and have a substitute decision-maker (SDM), their SDM will provide written informed consent in conjunction with participant assent to study participation. These broad eligibility criteria will serve to support generalizability of the sample and maximize the opportunity to evaluate the influence of common co-occurring illnesses on longitudinal outcome trajectories. Caregivers of study participants will also be invited to participate with the consent of the study participant.

**3.2 Procedures**

Recruitment and Informed Consent:

At the initial baseline visit, participants and their SDM where applicable, will be provided detailed study information via the informed consent form, and through discussion with the study team. The informed consent process will take place in person or through Cisco Webex video conferencing and REDCap as described in CAMH SOP #HSR 229: e-Consent Framework. Study staff will assess eligibility via the inclusion/exclusion criteria following obtaining consent. No study procedures will be completed prior to obtaining informed consent.

Participants will be recruited through the Schizophrenia Division Central Recruitment Model (REB #036/2020), the CAMH Research Registry, physician/clinician referrals, and through direct contact with former research participants who participated in previous studies of the Principal Investigators and Co-Investigators of this study who consented to be contacted for future research studies. If a patient is interested to learn more about the study, the physician/clinician may pass the patient’s preferred contact information to a research staff for follow up. A study recruitment flyer will be shared with participants who need more information about the study. This flyer will also be posted on bulletin boards across CAMH. Study information will also be available to participants through the CAMH Research Connect website.

The Schizophrenia Division Centralized Recruitment Procedure will be used to recruit participants for this study. The Centralized Research Personnel will prescreen patients for eligibility to participate using minimal inclusion/exclusion criteria outlined in REB #036/2020. Once a patient has been identified, the Centralized Research Personnel will send preferred contact information to the Study Specific Research personnel who will reach out to the patient to further explain the study. No information will be passed to the Study Specific Research Personnel prior to the patient’s verbal consent.

For e-Consent, participants will be provided with a read-only copy of the informed consent form via REDCap prior to conducting the consent discussion. The link may be used by participants as many times as they wish (it is not single-use). Upon clicking the link, participants or their caregiver/substitute-decision maker will review the landing page, and continue on to the informed consent form text. The entire contents of the informed consent form will be displayed according to the current REB approved consent form, minus the signature/attestation page(s). Informed consent will be documented using the REDCap e-Consent Framework. Following the consent discussion, the prospective participant or their caregiver will be sent a link to the e-consent via email or the chat feature in WebEx. The participant will complete the e-consent and be provided with the option to download and/or email themselves the signed informed consent form. If email is chosen, the email will only be used for this purpose (it is not retained by REDCap). Following the participant and/or caregiver signature, the person conducting the consent discussion will complete the Person Conducting Consent Discussion Attestation Page. PDF copies of the signed informed consent forms and Attestation pages will be retained in the REDCap File Repository. The research team will provide the participant and/or their caregiver with a copy of the fully signed informed consent form via mail and/or email, in accordance with the participant’s wishes. When the informed consent process takes place in person, participants and/or their caregivers will be provided a copy of the fully signed informed consent form in person at the time of consent. As consent is an ongoing process, even after a participant has provided initial consent to participate, we will use subsequent visits and the implementation of study procedures and assessments as opportunities to again explain what is being done and to ensure continued informed consent.

Study Procedures:

Following written informed consent, enrolled participants will undergo comprehensive clinical and functional characterization using a series of standard measures, including structured diagnostic assessment, clinical assessments to index severity of psychiatric symptoms, substance use, physical health, cognition and functioning, collection of treatment and medical history data from participants and their electronic medical record, as well as laboratory, genetic, and imaging assessments (described in detail in Sections 3.3 - 3.7). This will be complemented by characterization of social and health equity factors and linkage with broader provincial health system data held at the Institute for Clinical Evaluative Sciences (ICES). Study visits will take place at baseline and every 6 months over a period of 3 years as outlined in Appendix 1. Participant assessments will be conducted either in-person or virtually, where possible, based on participant preference.

Study Withdrawal/Termination:

Reasons for withdrawing individual participants from the study may include one or more of the following: a) major protocol violation b) participant lost to follow-up c) withdrawal of consent.

Any participant may be discontinued from the study at the discretion of the investigators if this is deemed to be in the best interest of the participant. The decision may be made either to protect the participant’s health and safety, or because it is part of the research plan that people who develop certain conditions may not continue to participate.

If a participant initially consents to participate and later chooses to withdraw from the study, they will be offered the opportunity to withdraw data and/or samples that have not yet been anonymized, published, or disseminated.

**3.3 Measures of personal recovery, disability, and community functioning**

A broad assessment approach will be utilized to evaluate co-primary and complementary constructs of personal recovery, disability, and community functioning. Evaluation will utilize a combination of participant self-report for the evaluation of personal recovery and disability, and clinician/rater-administered evaluation of community functioning.

Co-Primary measures:

*Recovery Assessment Scale (RAS)*

The 22 item version of the RAS will be used to assess personal recovery. This self-rated measure uses a five point scale ranging from 0 (“Strongly Disagree”) to 4 (“Strongly Agree”) and provides an overall score as well as scores on five subscales which are based on the following 5 factors: personal confidence and hope, willingness to ask for help, goal and success orientation, reliance on others and no domination by symptoms [(Corrigan et al., 2004)](https://www.zotero.org/google-docs/?158VJD). The RAS is the most widely used measure of person-centered recovery in the literature with well-established psychometric properties, including good validity as assessed in 49 studies and good reliability as assessed in 19 studies. It is also sensitive to change over time and has been used in numerous longitudinal studies and RCTs [(Salzer & Brusilovskiy, 2014)](https://www.zotero.org/google-docs/?lgnb1k).

*World Health Organization Disability Assessment Schedule 2.0 (WHO-DAS)*

Disability will be evaluated using the World Health Organization (WHO) Disability Assessment Schedule 2.0 (WHO-DAS), a 36-item measure of disability and health that is grounded in the framework of the WHO’s International Classification of Functioning, Disability and Health [(Ustun et al., 2010)](https://www.zotero.org/google-docs/?WNIYP9). The WHO-DAS enables comprehensive evaluation of individuals’ functioning across six major life domains (cognition, mobility, self-care, getting along, life activities, and participation in society) across different populations. The WHO-DAS has been shown to have good psychometric qualities including good reliability, a stable factor structure across populations, concurrent validity, and is sensitive to change over time [(Ustun et al., 2010)](https://www.zotero.org/google-docs/?4aq4iq). In order to capture diverse perspectives on participants’ trajectories over time, we will employ both the participant self-report version as a primary outcome measure, and where available will also seek input from the informant-version of the WHODAS.

*Personal and Social Performance scale (PSP)*

The PSP will be utilized to capture the trajectory of clinician/rater-measured community functioning. The PSP is an anchored version of the Social and Occupational Functioning Assessment Scale (SOFAS) from the DSM-IV that enables separate evaluations across community functioning domains for individuals with SSDs including role functioning, social functioning, self-care, and disturbing and aggressive behaviours [(Morosini et al., 2000)](https://www.zotero.org/google-docs/?DtRe8w). Ratings across these separate subscales serve to anchor global ratings of community functioning on a scale from 1 to 100. The PSP has been shown to demonstrate good test-retest and inter-rater reliability, validity, and sensitivity to change over time [(Morosini et al., 2000; Nasrallah et al., 2008)](https://www.zotero.org/google-docs/?ZtYw8F).

Secondary measures:

*Canadian Personal Recovery Outcome Measure (C-PROM)*

In addition to the above primary outcome measures, we will augment our assessment of personal recovery with the C-PROM, a 30 item measure of personal recovery initially developed at CAMH with service-user input from the clinical services that the proposed cohort will be recruiting from [(Barbic et al., 2018)](https://www.zotero.org/google-docs/?eyjLyM). At the present time this measure has been used across clinical services in the Canadian context and early psychometric data has found it to be valid and reliable (correspondence from the author). The C-PROM has also been identified as an easy to interpret measure that can inform assessment, care planning and communication between clinicians, service-users and family members. Inclusion of the C-PROM in this large longitudinal cohort study will also enable continued evaluation of this measure and its psychometric properties.

*WHO Quality of Life Brief Version (WHOQOL-BREF)*

The WHO Quality of Life Scale Brief Version (WHOQOL-BREF) is an abbreviated 26-item version of the WHOQOL-100 [(World Health Organization, 1996)](https://www.zotero.org/google-docs/?9RruXQ). It assesses quality of life (QOL) across four domains: physical, psychological, social and environment. The WHOQOL-BREF has good to excellent reliability and is a valid measure of QOL. It is also a cross-culturally valid assessment of QOL, with psychometric properties analyzed in 23 countries [(Skevington et al., 2004)](https://www.zotero.org/google-docs/?SMmT7E). Psychometric properties of the WHOQOL-BREF have been assessed in people with SSD and it has been found to be a valid and reliable measure that can detect different aspects of QOL in this clinical population [(Mas-Expósito et al., 2011; Su et al., 2014)](https://www.zotero.org/google-docs/?yzQFeQ). Inclusion of this measure in this cohort study will enable the longitudinal evaluation of health-related quality of life for people with SSDs (as it relates also to physical health), and relationships with functional outcome trajectories.

**3.4 Demographics**

Best practice to collect participant demographics will be used and this will be collected through routine use of the CAMH Health Equity Form [(Wray et al., 2013)](https://www.zotero.org/google-docs/?peN2Qj) in addition to revised items from the PhenX Toolkit Demographic Protocol (Stover et al., 2010). This will permit the systematic collection of data related to biological sex, gender, ethnicity, immigration history, educational attainment, employment, income, and housing. In addition to the collection of these demographic data, more specific measures assessing socio-environmental exposures and service use will also be utilized with specific details outlined in the relevant sections below.

**3.5 Mental health diagnostic and psychopathology measures**

*Clinical measures*

All participants will be evaluated using the Structured Clinical Interview for DSM-5 (SCID-5) [(First et al., 2015)](https://www.zotero.org/google-docs/?IdZLOd) to establish current psychiatric diagnoses, as well as dimensional assessment for symptom severity using the 24-item Brief Psychiatric Rating Scale (BPRS) [(Ventura et al., 1993)](https://www.zotero.org/google-docs/?MFCBBn), the self-report Modified Colorado Symptom Index (mCSI) (Conrad et al. 2001), the Scale for the Assessment of Negative Symptoms (SANS) [(Andreasen, 1982)](https://www.zotero.org/google-docs/?5D05wL), the Clinical Global Impression (CGI) scale [(Guy, 1976)](https://www.zotero.org/google-docs/?7e8GMH), the VAGUS insight into psychosis scale [(Gerretsen et al., 2014)](https://www.zotero.org/google-docs/?qmL1cz), and the Drug Attitude Inventory (DAI) [(Hogan et al., 1983)](https://www.zotero.org/google-docs/?YpocbM) to evaluate subjective perspectives on medication treatment. Presence and severity of suicidality will be evaluated using the Columbia Suicide Severity Rating Scale (CSSRS) [(Posner et al., 2011)](https://www.zotero.org/google-docs/?U1pAoU). Severity of substance use will be evaluated using the Alcohol Use Disorders Identification Test - Consumption (AUDIT-C) [(Bush et al., 1998)](https://www.zotero.org/google-docs/?ve0IsZ), the NIDA Modified ASSIST tool (question 2 that evaluates substance use frequency specifically) [(NIDA, 2012)](https://www.zotero.org/google-docs/?xIAXVT), Daily Sessions, Frequency, Age of Onset and Quality Cannabis Use Inventory (DFAQ) (Cuttler & Spradlin, 2017), and the CAMH Tobacco Screening Tool.

*Cognition*

Neurocognitive symptoms will be evaluated using the Brief Assessment of Cognition in Schizophrenia (BACS), an assessment of global cognitive functioning that includes assessment of verbal memory, digital sequencing, working memory, motor speed, verbal fluency, symbol coding, attention and processing speed and executive function. (Keefe et al., 2004)

Social cognition measures will also be utilized to evaluate distinct lower-level emotion identification and higher-level mentalizing facets of social cognition, demonstrated by previous work from our group [(Oliver et al., 2019)](https://www.zotero.org/google-docs/?4rSq2F). In line with our previous work, we will use the Penn Emotion Recognition task (ER-40) and The Awareness of Social Inference Test (TASIT) to assess lower- and higher-level social cognition, respectively. The ER-40 measures one’s ability to decode and correctly identify facial expressions of emotion based on static images [(Kohler et al., 2000)](https://www.zotero.org/google-docs/?WNqszI). The TASIT utilizes videos of naturalistic everyday conversations involving two actors to evaluate social perception and inference [(McDonald et al., 2011)](https://www.zotero.org/google-docs/?1iIS1V). To minimize participant burden while ensuring adequate assessment of higher-level social cognition, we will be using the short version of the TASIT (TASIT-S), which contains a subset of stimuli from the original TASIT, has been validated (Honan et al. 2016), and has shown an identical factor structure and loading on facets of social cognition in keeping with our previous work [(Oliver et al., 2019; unpublished data)](https://www.zotero.org/google-docs/?4rSq2F).

*Treatment history and medication side effects*

Medication treatment history will be captured through the PhenX Toolkit medication inventory, as well as from the participant’s electronic medical record. A timeline of treatment and hospitalizations will also be ascertained through participant interview coupled with data available through the participant’s electronic medical record and provincial health administrative data (outlined below).

Antipsychotic-induced motor side effects will be evaluated using the Abnormal Involuntary Movement Scale (AIMS) [(Lane et al., 1985)](https://www.zotero.org/google-docs/?LU2lh9), the Simpson Angus Rating Scale (SARS) [(Simpson & Angus, 1970)](https://www.zotero.org/google-docs/?DQXN6S), and the Barnes Akathisia Rating Scale (BARS) [(Barnes, 1989)](https://www.zotero.org/google-docs/?fuOefU), and the short Neurological Evaluation Scale (S-NES) (Ojagbemi, Emsely, & Gureje, 2017). The self-report Subjective Well-being under Neuroleptics (SWN) scale [(Naber, 1995)](https://www.zotero.org/google-docs/?zU3S1t) will be used to index severity of antipsychotic-induced effects on subjective well-being, while the UKU side effect rating scale will be utilized to evaluate other common antipsychotic side effects [(Lingjaerde et al., 1987)](https://www.zotero.org/google-docs/?kS0yOV).

**3.6 Physical health measures**

Participant’s physical health status will be evaluated according to current metabolic monitoring guidelines, as part of routine clinical care (see Table 1 below). In addition, a comprehensive medical history will be conducted, and will be augmented by data in the participant’s electronic medical record (EMR). For purposes of study participation these routine clinically-indicated measures will be aligned with other study-related measures (without incurring additional costs). We will also use EMR data to look at prescription patterns of antidiabetic, antihypertensive, and lipid lowering medications. Tobacco use will be assessed via the CAMH Tobacco Screening Tool. The Obesity Adjustment Survey (OAS) Short Form is a validated brief self-report measure and will be used to assess distress and psychological impact of weight and obesity [(Butler et al., 1999)](https://www.zotero.org/google-docs/?Rkkmbp). Health related quality of life will be captured by the WHOQOL-BREF. At study entry, a tube of serum will be collected and stored for future metabolomics analysis.

**Table 1**: Routine physical care measures

|  | Routine measurement based care (MBC) data collected | **BL** | **3 Mo** | **6 Mo** | **1 Yr** |
| --- | --- | --- | --- | --- | --- |
| **Metabolic, Activity, QofL Assessments** | Personal/Family History of CVD/Lifestyle review (diet, activity, smoking) | X |  |  | X |
|  | Weight and waist circumference. BMI is calculated (weight/height ^2^ ). | X | X | X | X |
|  | Vitals, Blood Pressure | X | X^ | X | X |
|  | Fasting Blood Glucose and insulin, HbA1c, CRP  (HOMA-IR, and index of insulin resistance is calculated from fasting glucose and insulin levels) | X | X^ | X | X |
|  | Lipid Profile (fasting if possible), liver, kidney function, Thyroid (TSH) | X |  | X* | X |
|  | Simple Physical Activity Questionnaire (SIMPAQ)  Tobacco Use (CAMH tobacco screening tool)  Obesity Adjustment Survey-Short Form (OAS-SF)  WHO Quality of Life Brief Version (WHOQOL-BREF) | X |  | X | X |

*^ Repeated at 3 months, if starting or switching new antipsychotic medication, and/or initiating adjunctive pharmacological intervention to target metabolic comorbidity.*

** Repeated yearly unless abnormal*

*BL: Baseline*

**3.7 Biological measures**

In addition to the above laboratory assessments related to physical health, study participants will be offered the opportunity to also participate in the following biological assessments, through this study as well as parallel studies at CAMH where these assessments are included and results shared.

**3.7.1 Genetics**

Genomic DNA will be purified from blood samples collected at baseline (at follow-up visits if additional fundings become available) using a chemagic^TM^ MSM I DNA extractor (Perkin-Elmer, Waltham, MA) following manufacturer protocol. If blood samples are not available, saliva samples (Oragene DNA, DNA Genotek) will be collected and used for DNA extraction. DNA will be quantified and assessed for quality using Cupid and diluted to a final concentration of 50 ng/µL. DNA samples will be genotyped on a genome-wide genotyping array (e.g., the Infinium Global Screening Array) at CAMH and quality-controlled using standard procedures using the Specialized Computing Cluster (PLINK and R; e.g.,  [Zai et al., in press; Zai et al., 2015)](https://www.zotero.org/google-docs/?HWEcwV). Genetic analyses include individual-marker association tests (PLINK), polygenic risk score and genetic correlation analyses (e.g., PRSice2, LDpred, LDSC), and gene-based and gene-set analyses (e.g., MAGMA/FUMA). With additional funding, other molecular genetic experiments of interest may include whole-genome sequencing, transcriptomic assays, epigenomic arrays, and pharmacogenetic test panels (e.g., [Herbert et al., 2018](https://www.zotero.org/google-docs/?G7CnsQ)). In collaboration with an existing NIMH study on schizophrenia genetics (site PI: Jim Kennedy; co-Is: Victoria Marshe, Arun Tiwari, Clement Zai), we will aim to collect blood samples from schizophrenia patients of African ancestry, leveraging funding support from that existing study to expand the collection of blood samples and genetic analyses as part of the PREDICTS study.

**3.7.2 Imaging**

MRI will be conducted at baseline at CAMH on a GE Discovery 750. Our proposed brain acquisitions have been rigorously tested as part of the Adolescent Brain Cognitive Development (ABCD) study. These acquisition parameters (published by ABCD) have been optimized for high-quality T1-weighted, diffusion MRI (dMRI) and R-fMRI data ensuring key parameters consistent across sites, (voxel size, number of diffusion-weighted directions, flip angle, etc.), and notably include multi-band acquisitions for echo-planar imaging (BOLD and dMRI).

The “ABCD Package” is GE Healthcare’s name for a set of pulse sequences designed to match those being used by the ABCD study. They include methods for acquiring data for structural and functional MRI at higher spatial and/or temporal resolution. These sequences are considered investigational and CAMH has obtained them as a "Work in Progress" (WIP) through a contract with GE. To comply with the contract, the consents include language indicating that investigational methods are being used, that there is no additional risk, and that anonymized data may be shared with collaborators. When these sequences become part of the “stock” sequences available on the scanner, we will update the consents. In this study, we will be using these sequences in order to better match data with other sites that have Siemens scanners.

ABCD-harmonized T1-weighted, multi-shell diffusion MRI, and resting state fMRI (R-fMRI) data will be collected. Given the importance of visceral fat and its impact on functional outcomes, we will include abdominal MRI. We have chosen the ABCD (as opposed to Human Connectome Project, HCP) acquisitions, as ABCD acquisitions are currently being proposed for local studies (for example, in the Toronto Adolescent and Youth (TAY) cohort which will allow for longitudinal analyses on a subsample of patients) and employed in active CAMH studies that include international collaborations. T1-weighted brain images are collected for anatomical measures. For abdominal imaging, aT1-weighted abdominal image is collected to improve segmentation and better characterize adipose tissue [(Garnov et al., 2014; Hu et al., 2016)](https://www.zotero.org/google-docs/?JYAgrc). A state-of-the-art water-triglyceride fat separation method (IDEAL-IQ) is then employed for measuring visceral adiposity. This method uses a chemical-shift-based, three-dimensional, volumetric pulse sequence, providing whole-liver coverage in a single breath-hold and enables accurate estimation of relative triglyceride fat fraction maps by correcting for T2* and performing multi-peak fat spectrum modeling [(Yu et al., 2008, 2011)](https://www.zotero.org/google-docs/?ZHszYi). Total scan time, including brain and abdominal imaging, is approximately 50 minutes, including the time to remove the radiofrequency head coil and replace it with a body coil.

*MRI data analysis*

Structural and Resting State Functional MRI Analysis: These scans will be preprocessed together using the CAMH-developed and publicly released Ciftify pipeline [(Dickie et al., 2019)](https://www.zotero.org/google-docs/?ls6777). This pipeline incorporates workflows from FreeSurfer ([Fischl, 2012](https://www.zotero.org/google-docs/?nTXnek)) for structural preprocessing and analysis and fMRIPrep for functional preprocessing [(Esteban et al., 2019)](https://www.zotero.org/google-docs/?cPNiLw). Notably, the Ciftify pipeline is a lead implementer of the Human Connectome Project’s newly introduced (Connectivity Informatics Technology Initiative) file format (CIFTI) for multimodal neuroimaging data.

Diffusion MRI Analysis**:** After preprocessing (including eddy current correction, nonlinear EPI distortion correction filtering, and tensor estimation), we will calculate diffusion measures (fractional anisotropy, axial diffusivity, radial diffusivity, and mean diffusivity) to investigate white matter connectivity and integrity. Furthermore, researchers at CAMH have recently shown multi-shell diffusion combined with novel non-tensor models can reliably generate measures of free water, diffusion kurtosis, the orientation dispersion index (ODI), and neuritic density index (NDI) in gray matter [(Nazeri et al., 2015)](https://www.zotero.org/google-docs/?4p16yc)*.* Moreover, ODI has been associated with cognitive performance in aging and changes in NDI in psychiatric disorders [(Nazeri et al., 2017)](https://www.zotero.org/google-docs/?EDuef9). These measures will be combined with measures from the structural pipeline to drive multi-modal profiles of “brain-age” trajectories.

Extracting fMRI Features with High Reliability: In terms of reliability, we are encouraged by the concept of generalized connectivity posed by Elliot et al (2019) and championed by Zuo and Milham (2019) where all BOLD fMRI can be concatenated together to increase the reliability of the BOLD connectivity signals. Under this framework, we anticipate that we may see r > 0.4 for > 50% of individual edges [(Elliott et al., 2019)](https://www.zotero.org/google-docs/?OnLYND).

Abdominal imaging: In house scripts will be used to obtain liver fat percentage. Selective thresholding along with automated and supervised contouring will be utilized to estimate subcutaneous (SAT) and visceral fat (VAT). SAT and VAT for each participant will be measured by segmenting the appropriate fat pixels on each of the acquired slices and then measuring the total volume of the segmented pixels in mL.

**3.8 Social, environmental, and health system measures**

Building on the basic demographic data collected on immigration history and ethnicity the 20 item Vancouver Index of Acculturation – Short (VIA) will be used to measure acculturation [(Ryder et al., 2000)](https://www.zotero.org/google-docs/?Nn4E0c), the Multi-Group Ethnic Identity-Revised (MIEM-R) a 12 item measure will further characterize cultural affiliation [(Brown et al., 2014)](https://www.zotero.org/google-docs/?XHBTx5) and the 8 item PhenX Acculturation Survey will be used to characterize language preferences [(Stover et al., 2010)](https://www.zotero.org/google-docs/?zXRTd1).

Lifetime discriminatory experiences across a wide range of domains will be measured using a Canadian adaptation of the Everyday Discrimination Scale (EDS) [(Canadian Community Health Survey, 2013; Williams et al., 1997)](https://www.zotero.org/google-docs/?wr4dM8). The Internalized Stigma of Mental Illness (ISMI), a 29 item questionnaire, will be used to measure self-stigma.

Life events and stressors will be measured using the 17 item Life Events Checklist (LEC) [(Gray et al., 2004)](https://www.zotero.org/google-docs/?ApQf55) and the Childhood Trauma Questionnaire – Short Form (CTQ-SF) will be used to identify early childhood traumas [(Bernstein et al., 1997)](https://www.zotero.org/google-docs/?zaGlxo).

Resilience in the face of stress will be measured using a brief two item version of the Connor-Davidson Resilience Scale [(Connor & Davidson, 2003)](https://www.zotero.org/google-docs/?OJBTPE) and self-esteem will be measured using the 20 item Self-Esteem Rating Scale-Short Form (SERS-SF) [(Lecomte et al., 2006)](https://www.zotero.org/google-docs/?oZVLHX). Perceived social support from family and other sources will be measured using the Multidimensional Scale of Perceived Social Support (MSPSS) [(Zimet et al., 1990)](https://www.zotero.org/google-docs/?negRev).

*Neighbourhood Social Environment*

The Ontario Marginalization index will be used to obtain a measure of area-level marginalization that will enable each participant's neighbourhood to be characterized by the level of social and economic marginalization as well as ethnic concentration [(Matheson et al., 2012)](https://www.zotero.org/google-docs/?qlS2i5). Postal codes of participants which will be collected as part of routine demographic data will be linked to this census-based index.

Participant’s perceptions of their proximal social environment will be further characterized using the three item Neighbourhood Safety and Crime Scale to ascertain a level of perceived neighbourhood safety [(Mujahid et al., 2007)](https://www.zotero.org/google-docs/?RSbwgm) as well as the 9-item, short version of the Adapted Social Capital Assessment Tool (A-SCAT) which characterizes both structural and cognitive social capital [(DeSilva, et al., 2006). )](https://www.zotero.org/google-docs/?znmbtB).

*Employment History*

Vocational Time-Line Follow-Back (VTLFB) will be used to obtain details on previous work, work-related training and income. This measure was created and used in the Canadian RCT of which showed the generalizability of the Individual Placement and Support (IPS) model of supported employment in the Canadian context [(Latimer et al., 2006)](https://www.zotero.org/google-docs/?0IWiqv). This measure was subsequently used in the At Home/Chez Soi RCT to characterize work history [(Goering et al., 2011)](https://www.zotero.org/google-docs/?kIPHrR).

*Housing Stability*

The Residential Timeline Follow-Back (RTLFB) will be used to capture key information about a person’s housing status including number of moves, reasons for moves, type of residence and household composition. Similar to other time follow-back measures, it uses a calendar to prompt participants to collect details for specific time periods. The RTLFB is a valid and reliable measure to assess residential stability across different populations [(Tsemberis et al., 2007)](https://www.zotero.org/google-docs/?6q87Be). Considering the lookback period is 6 months, we aim to use this measure at key intervals throughout the follow-up period. This measure has also been adapted for use in the Canadian context and was used in the At Home/Chez Soi RCT to characterize housing stability [(Goering et al., 2011)](https://www.zotero.org/google-docs/?qkcjz2).

*Service Use*

The Health, Social and Justice Service Use Inventory (HSJSU) was developed to assess service use and estimate services costs as part of the large multi-site At Home/Chez Soi RCT of a Housing First intervention [(Goering et al., 2011)](https://www.zotero.org/google-docs/?YGRSvY). This tool was initially used to evaluate service use in a sample of Canadians with serious mental illness experiencing homelessness and developed based on a number of prior tools and measures with varying psychometric properties [(Guerriere et al., 2006; Kashner et al., 2009; Sirey et al., 2005)](https://www.zotero.org/google-docs/?HJgPWD). Through the proposed linkage with administrative health data that will be undertaken as part of this study validation of this Canadian designed measure will be undertaken.

**3.9 Health administrative data**

All participants in the study will have the opportunity to consent to have their data linked with administrative health data held at ICES. Linkage between administrative data holdings at ICES are conducted using a unique identifier for all people eligible for OHIP funded services in the province of Ontario. Data linkage will allow for the use of additional contextual data that can help further identify trajectories as well as real-world outcomes that may occur outside of the context of care at CAMH. For example, hospitalizations at other mental health and medical facilities, ER contacts, contact with primary care physicians, as well as data on mortality and morbidity. All linked data will be deidentified and held and analyzed at ICES. ICES is a prescribed entity under section 45 Ontario's Personal Health Information Privacy Act and all practices and procedures relating to data use are regularly reviewed and approved by the Office of the Information Privacy Commissioner/Ontario.

The proposed data linkage will allow for 1) the evaluation of real world outcomes and services use in cohort participants for a longer duration during a passive follow-up period and 2) comparison to other people with SSDs who may be receiving similar services in the community or have a SSD but are not connected with services. The proposed methodology has recently been used to evaluate the real-world effectiveness of early psychosis intervention services in London, Ontario [(Anderson et al., 2018)](https://www.zotero.org/google-docs/?UFkg3e) and is increasingly being used in other jurisdictions to evaluate real-world outcomes of mental health services where health administrative data are available [(Harris et al., 2019; Mötteli et al., 2018; Stroup et al., 2015; Yu et al., 2009)](https://www.zotero.org/google-docs/?mseosv). Moreover, this methodology and use of health administrative data can contribute to building evidence in settings where randomized trials may not be feasible and can be limited by small sample sizes (which precludes the study of rare events), strict inclusion criteria (which may limit real-world applicability), short duration of follow-up and other ethical considerations.

**3.9.1 Study population and design**

Participants who consent to have their data linked with administrative health data will be eligible to have both historical and future services use linked from the time of cohort entry. Participants who have a OHIP card will be able to consent for direct linkage, and participants who consent but have any uncertainty with their OHIP number will be linked using a deterministic process.

Further use of administrative data will enable comparison of service use and outcome between:

1. Participants that are part of the cohort at CAMH (accessing specialized mental health services at CAMH),
2. People with a SSD who are accessing specialized mental health services in the community (including ACT, ICM and case management services but not at CAMH), and
3. People with a SSD who reside in Toronto but are not currently receiving specialized mental health services

Diagnosis of a SSD in the comparison groups in the health administrative data will be ascertained using a validated algorithm that captures all incident cases of SSD [(Kurdyak et al., 2015)](https://www.zotero.org/google-docs/?SkNgwy). The comparison groups will be restricted to people who reside in areas of Toronto where outpatient services at CAMH are offered and restricted to people who have not received regular outpatient services at CAMH.

**3.9.2 Propensity score matching**

Propensity score matching is proposed to identify people that will be comparable to the cohort participants. By definition, a propensity score is the probability of treatment assignment conditional on observed baseline covariates. Therefore, among people with the same propensity score, the distribution of observed baseline covariates will be the same between treated and untreated groups. An estimated propensity score can be obtained by regressing treatment status on observed baseline covariates. To reduce observed confounding participants can be matched based on the propensity score. Estimated treatment effects can then be obtained by directly comparing outcomes between matched groups using appropriate methods for matched samples. Propensity score matching mimics some aspects of randomization of a traditional trial but uses observational data. However, unlike a randomized trial, propensity score matching can only account for measurable confounding and not unmeasurable confounding.

For the purpose of this study a propensity score will be calculated based on all covariates that can impact treatment assignment and outcomes. Linked cohort participants will be matched to people in the health administrative data based on 1:1:1 ratio, without replacement, on the propensity score to ascertain two additional exposure groups. This will enable the comparison of three exposures (CAMH services, community services, eligible but no service).

**3.9.3 Data sources**

Linkages will occur across the following ICES data holdings: the Registered Persons Database (RPDB) which is a central population registry that contains basic demographic data on all people insured by OHIP; the Ontario Mental Health Reporting System (OMHRS) which contains data on all inpatient hospitalizations to adult mental health beds; the Canadian Institute for Health Information Discharge Abstract Database (CIHI-DAD) containing data on all acute care hospitalization and mental health hospitalizations before 2005; the National Ambulatory Care Reporting System (NACRS) which contains data on emergency department visits; outpatient physician billings from OHIP; the Ontario Drug Benefit (ODB) claims which contains data on medications dispensed through publicly funded program for eligible people; the Ontario Laboratories Information System (OLIS) containing laboratory data across the province, the Ontario Registrar General (ORG) for information on deaths; and the Ontario Marginalization Index (ON-Marg) which is an area-level deprivation index based on census data

**3.9.4 Outcomes**

The primary outcome will be the number of mental health hospitalization following the cohort entry period. Secondary outcomes will include the number of medical hospitalizations, days spent in hospital (both in mental health and medical beds), ED visits, suicide and self-harm attempts and mortality.

**3.9.5 Covariates**

Covariates that will be accounted for will include: age, sex, immigration status, neighbourhood-level marginalization (consider neighbourhood as well), diagnosis, history of substance use related health care contacts, enrollment with a family physician, ODB eligibility, long-acting antipsychotic medication claims, claims for medications for specific comorbid health issues (i.e., metformin, statins), regular metabolic monitoring, regular CBC monitoring for clozapine use (as at the present time clozapine claims are not regularly reported).

**3.9.6 Data Analysis**

Descriptive statistics will be used to characterize outcomes and covariates. Balance between groups will be assessed. Based on the type and distribution of the outcome data appropriate regression models will be fit accounting for the matched nature of these data.

*Cost analysis*

A standard ICES costing methodology and algorithm [(Wodchis et al., 2013, 2016)](https://www.zotero.org/google-docs/?IQrY21) will be used to estimate all health care costs incurred within the publicly funded provincial health care system. Costs will be identified for all hospitalizations (psychiatric and non-psychiatric), ED visits, physician visits (across all specialties), diagnostic tests, prescription drugs of those covered under via ODB, as well as other publicly funded services. Costs will be compared between comparison groups as well as between trajectories identified within the cohort.

**3.10 EMR-based Natural Language Processing (NLP) and Machine Learning (ML) for the characterization of longitudinal clinical course and outcomes**

Data will be extracted from the CAMH EMR and the Enterprise Data Warehouse for EMR-based retrospective characterization of the longitudinal clinical course and outcomes for study participants. Structured data elements that overlap with measures utilized for prospective cohort characterization (described above) will include sociodemographic data, administrative data related to treatment encounters, as well as clinical data (including diagnoses, medical problem lists, prescribed medications, laboratory data, anthropometric measures, and structured assessments of psychopathology (e.g., BPRS, CGI, BCATS) and functioning (e.g., WHO-DAS, PSP/SOFAS). In addition, free text clinical notes pertaining to consenting study participants will be also extracted.

To evaluate various approaches for preprocessing and extracting features from the free text clinical notes of study participants, we will first analyze any available structured data (i.e., demographics, visit frequency, outcome measures) and free text clinical notes pertaining to patients with schizophrenia spectrum disorders at CAMH (Phase 1). This will be followed by our second analyses (Phase 2) that comprise the analytic approaches developed in Phase 1 applied and validated with the free text clinical notes for consenting participants that are enrolled in this study. For Phase 1, however, because these notes are analyzed without patient consent, we will use REB-approved protocols developed in other studies (e.g., 157-2018, 156-2020), which ensure that the study team only access numerical features derived from the notes. Accessing and analyzing these numerical features, instead of the notes in their raw form, reduces privacy risks of accessing sensitive or identifiable information contained within the notes.

Before any clinical notes from the CAMH medical records are accessed by the study team (i.e., for study participants and CAMH patients), they will be de-identified. Chris Wakefield, Integration Manager with IMG Clinical Applications at CAMH, will extract a copy of the clinical notes from I-CARE and securely transfer this copy of the raw data to the enterprise data warehouse on secure clinical servers, where their access is restricted to IMG Clinical Applications personnel authorized to support this study. At this point, clinical applications will configure CRATE, an anonymization software [(Cardinal, 2017)](https://www.zotero.org/google-docs/?jYkokH), to remove direct identifiers from the clinical notes. The software’s configuration will be refined to achieve the highest possible recall, with an estimated 89-100% of the direct identifiers being removed from the clinical notes. These direct identifiers include names, dates of birth, medical record numbers (MRNs), address components, telephone numbers, email addresses, aliases and third-party information (i.e., names of family members or other non-professional contacts). IMG accomplishes this configuration by securely populating the CRATE data dictionary with direct identifiers (such as, but not limited to, names, phone numbers, MRNs) associated with existing CAMH clients from I-CARE, with a large database of names, surnames, and addresses (independent of those added from I-CARE), and regular expressions, which are characters that represent patterns of text (e.g., phone number patterns, medical number patterns), similar to wildcards. Once the data dictionary is generated, CRATE works by comparing words in the clinical notes against those in the data dictionary. If there is a match, it removes those words from the clinical notes. Once CRATE is configured, clinical applications will perform unit tests, which involve passing texts with only potential identifiers in them (such as names and sets of dummy phone numbers, MRNs, e-mail addresses) through CRATE. If CRATE purges 100% of the document (i.e., no text remains), it will be deemed successful enough to be used on the clinical notes. At this point, CRATE will be used to de-identify the clinical notes.

For Phase 1, involving analyses on retrospective clinical documentation pertaining to CAMH patients with schizophrenia spectrum disorders, IMG applications personnel will implement NLP methods (similar to those described below for Phase 2 analyses) to convert the clinical notes into features, which will then be shared with the study team. This process ensures that the study team do not access raw clinical notes for retrospective patients who have not formally provided their consent to participate in the study. For these patients, only IMG personnel will have access to the clinical notes in their raw form, no one (not even IMG personnel) will see or review individual notes, and what is extracted and accessed by the research team will not be directly or indirectly identifiable. The study team will also access the following structured data pertaining to the CAMH clients represented in the clinical notes: sociodemographic information from the Health Equity Form, CAMH visit frequency, type, and location, diagnoses, medical problem lists, prescribed medications, laboratory data, anthropometric measures, as well as any available outcomes measured with the WHO-DAS, BPRS, CGI, PSP, B-CATS, AIMS, BARS, and SARS. In Phase 1, we aim to develop and refine methods for extracting meaningful patterns ad trends from the clinical note features, which involves evaluating various text preprocessing and modelling strategies. The structured data will be used as part of this evaluation to validate patterns and trends identified within the notes, based on the various preprocessing and modelling strategies being investigated. We will also use the structured data to gauge the sociodemographic and clinical representativeness of the Phase 1 sample.

For Phase 2 analyses involving consenting study participants, once direct identifiers have been removed, the raw clinical notes for study participants will be shared with the study team, who will implement NLP methods in the R programming language to automatically convert the clinical notes into features. Converting the notes into features disrupts the order of the words and their context, or it reduces the text to numerical data representing various characteristics (e.g., polarity, semantics). For instance, the study team will generate a document-term matrix, containing words from the clinical notes as columns and their frequencies in each note as rows. This matrix will be utilized for topic modeling, an unsupervised ML algorithm to discover themes and generate temporal patterns and trends to investigate trajectories of outcomes and clinical phenomena in the study population. These temporal patterns will be linked with structured data for that participant available both from the EMR (including their age, sex, ethnicity, socioeconomic status, clinical and outcome measures where available (e.g., BPRS, CGI, WHO-DAS, PSP, B-CATS, AIMS, BARS, SARS), medication prescriptions, treatment encounters), as well as with the prospectively collected outcome, clinical, physical health, socioenvironmental, and biological data as part of this study.

We will store all study data (including data from the CAMH EMR, such as the structured data and de-identified clinical notes) on the CAMH Neuroinformatics platform for the entire duration of the study, and its availability will be restricted to the study team. The study team will use the remote server to access and analyze the data. If the study team wishes to use the data for purposes that were not outlined in the current version of the protocol, or if other individuals wish to access the data, the research team will submit an amendment or a new ethics application to the CAMH REB. The data will remain on the server, and it will never be downloaded on any computers or devices. The CAMH Neuroinformatics platform automatically tracks the activity of all its users; it keeps a record of individuals who log into the platform, the software they use, the data they access, and what they do with the data. This will allow for audits of who has accessed the document-term matrices and for what purpose these data were accessed.

**3.11 Needs assessment of interventions to improve outcomes and reduce disability in SSDs**

Within the cohort there is a unique opportunity to conduct a needs assessment of the currently available and accessible evidence-based interventions and promising practices that may improve outcomes and reduce disability in clinical populations with SSDs. We aim to obtain input from study participants on early trajectory data and predictors to identify specific gaps in care and needs that may not yet be met by evidence-based services offered at CAMH, or by CAMH partners, or can be improved upon.

Data collected during the 1st year of the study period will be analyzed with the goal of identifying preliminary trajectories. The distribution of predictor variables measured during this period will also be described (e.g., measures of physical health, cognitive functioning, medical comorbidities). Although it is possible that data from these early time points may not map exactly onto the final trajectories identified at the end of the cohort, it is anticipated these early data points will provide information on specific areas of need and or service gaps within the cohort. For example, differential service use patterns associated with comorbidities, baseline cognitive functioning, as well as metabolic and physical health profiles may be elucidated from these early data and may be important predictors of both preliminary categorization of function and recovery and final trajectories.

Following identification of preliminary trajectories and key predictors, participants will be invited to participate in a needs assessment in the 2nd year of the study. The focus of this needs assessment will be to identify specific gaps in functioning, care and services that are directly related to the initial trajectories. Information on service user experience with current services and treatment in conjunction with the presentation of initial trajectories will be used to determine whether gaps exist due to missing and/or misaligned services, process issues, or contextual issues. This process will occur in both individual and group settings using a semi-structured format. An inductive thematic analysis will be undertaken to identify and report emergent themes and patterns.

The outcomes of this process will also identify potential targets for future co-design treatment interventions that will be the focus of an adjunct to this study (and to be submitted at the time for REB review).

**4.0 DATA MANAGEMENT PROTOCOLS**

Demographic, clinical assessment, cognitive, health, socio-environmental, and outcome data via CAMH’s Research Electronic Data Capture (REDCap) platform. All MRI data will be uploaded to XNAT and automated pipelines used for standardized processing and QA/QC procedures. Labkey will be used to support tracking and management of biological samples and genetic analysis data. All data will be linked via each participant’s unique study ID. Data access controls will be utilized to restrict access to each tool to study team members requiring access to this data. Central data management will utilize the CAMH Neuroinformatics Platform. With informed consent from study participants, data from participants with an Ontario health card number will be linked with health care utilization data through the Institute of Clinical Evaluative Sciences (ICES).

Data collection will take place either in-person, remotely via REDCap survey link for self-report measures where applicable, or virtually through the use of the Cisco Webex videoconferencing platform, depending on participant preference, availability, and suitability for in-person study visits based on pandemic-related hospital guidelines for clinical research. Data will be collected on electronic or paper-based forms, which are identified by a unique participant ID number, protocol ID number, and protocol phase and visit, and are entered into a secured REDCap database housed at CAMH which will be managed by the study team. REDCap is a secure, web-based platform, which is used world-wide to collect all types of data ranging from research, to clinical, to operations and has features to support best practices for data confidentiality, security, data validation, auditing, and data backup.

A protocol data collection schedule will be used to monitor participant progress, missing assessments, and other protocol deviations during the study. Data entry screens will incorporate range checks or lists of valid responses for each item to ensure accurate data entry. Forms with missing or invalid data in key identifying fields will be referred back to raters for correction before entry. Other missing or invalid data will not prevent the form from being entered, but will be flagged for correction. Queries will be built around data entry and reports to review adverse events. Participant confidentiality will be maintained by restricting study data access to specified study personnel. Once data has been entered, an electronic audit trail will record all database changes. The database will reside on a central server, and all server data is backed up on a regular basis. Outside access to the server is restricted by a firewall. At the conclusion of the study, all study data will be archived and retained for the full period required by regulations and according to CAMH SOPs.

Health administrative data linkage will be done through ICES, a prescribed entity under Ontario’s Personal Health Information Protection Act that follows the policies and procedures for privacy protection and data security approved by Ontario’s Information and Privacy Commissioner. Participants’ primary data collected through this study will be securely transferred to ICES from the CAMH server via secure data transfer to be linked with administrative data. This secure, encrypted channel is established by ICES and managed by authorized ICES individuals. After assignment of a unique ICES identifier all identifiers will be removed and only de-identified data will be used to conduct analyses. Only ICES-approved and trained analysts are entitled to conduct primary data analysis in secure zones within ICES facilities. Data will only be accessed internally, using a password-protected monitored user account. Only aggregate results may be removed from this secure server. ICES practice is to destroy any data transferred for linkage at 180 days and to retain the coded data used for research for 10 years. The lookback period within the ICES health administrative data examined for individuals who have provided consent will either be their date of birth or when they first became eligible for OHIP coverage (e.g., if they moved to Ontario from another province or country). As a prescribed entity under PHIPA, ICES is permitted to hold and analyze data that are identifiable, but to only report publicly in a non-identifiable format.

*Data sharing permission from participants to reduce participant burden*

We will acquire permission from study participants to use data already captured in their electronic medical record. Many of the clinical services at CAMH where participants will recruited for this study already utilize some of the rating scales and collect some amount of sociodemographic, educational, and cognitive information as part of routine clinical care. For study participants where this information has already been collected as part of their routine clinical care, we will endeavour to utilize this already collected information rather than repeating these assessments. This will reduce participant burden by shortening the research assessment duration for study visits. In addition, we will acquire permission from study participants to share data collected as part of this study with other research studies they may take part in at CAMH, as well as permission for results of those studies (e.g., imaging, genetics, and laboratory assessments) to be shared with this study. This will serve to also reduce participant burden by shortening research assessment durations, as well as leverage other concurrent research studies to augment the biomarker collection plan for this study. Finally, we will also acquire permission from study participants to share the results of their research assessments from this study with their clinical team. Many of the clinical services at CAMH where participants will recruited for this study already utilize some of the rating scales (i.e., BPRS, CGI, PSP, SAS, BARS, AIMS, as well as self-report assessments including the WHO-DAS, AUDIT-C, NIDA Modified ASSIST, CAMH Tobacco Screener) and collect some amount of sociodemographic, educational, and cognitive information (i.e., CAMH Health Equity Form, BCATS), as well as physical health assessments (i.e., height, weight, BMI, waist circumference, metabolic laboratory assessments) as part of routine measurement-based clinical care. Other measures from this study not routinely collected may also be useful to their clinical team in supporting the participant’s clinical care.

*Open Science*

The PREDICTS Study will implement open science by utilizing processes and data governance being established by the BrainHealth Databank to enable and promote data sharing and reuse with researchers at CAMH and around the world. This includes implementation of study data management processes to ensure that study data adhere to the FAIR data principles – Findable, Accessible, Interoperable, and Reusable, collection of common data elements to facilitate data standardization and harmonization as appropriate, and inclusion of standardized informed consent to enable future data sharing and secondary use of data.

To support data sharing, a PREDICTS cohort explorer dashboard will be created to allow users to explore available data and formulate data access requests. Initially this dashboard will be used by PREDICTS researchers across core projects and data modalities to answer primary study questions. In the future, the PREDICTS study cohort explorer dashboard will be made available to other researchers at CAMH and around the world to enable secondary research studies. All information entered into these databases will be free of identifying information. Data access to researchers conducting secondary studies will be granted once appropriate approval, as defined in the BrainHealth Databank Data Access Policy, has been obtained.

*Confidentiality*

There is a potential risk of breach of confidentiality that is inherent in all research protocols. Breach of confidentiality will be minimized by the staff who will maintain research data (identified only by participant code number not related to name, or date of birth) in separate charts and a dedicated password protected electronic database. A list of participant names, their ID numbers, and information about how they can be reached will be kept in a separate locked cabinet with access only to study personnel authorized by the PIs. Procedures have been established, and will be followed, to minimize the risk of breach of confidentiality. Procedures to maintain confidentiality include: (1) formal training sessions for all research staff emphasizing the importance of confidentiality; (2) specific procedures developed to protect participants’ confidentiality, and (3) formal mechanisms limiting access to information that can link data to individual participants. All information obtained from participants will be kept as confidential as possible. Computer based files/data will be entered into password-secured databases and paper-based files will be stored in a secure location. These data will only be accessible to personnel involved in the study and they will abide by confidentiality regulations of the REB. The ethics committee will be granted direct access to the study participants’ original medical records for verification of clinical trial procedures and/or data, without violating the confidentiality of the participants, to the extent permitted by the law and regulations.

**5.0 DATA ANALYSIS PLAN AND POWER ANALYSIS**

Descriptive statistics will be used to summarize the data on all participants to understand the uni- and multi-dimensional characteristics of their distribution. Clustering of observations at multiple levels will be evaluated to provide guidance for selecting bias correction method and multilevel modeling approaches. We will further evaluate missing data patterns and potential bias that could be incurred by participants’ dropout.

For Objective 1, latent growth mixture modeling [(Ram & Grimm, 2009)](https://www.zotero.org/google-docs/?broken=pb8heR) will be primarily applied to permit a person-centered approach for identification of homogeneous subgroups of individuals with distinct longitudinal outcome trajectories across our three co-primary outcome measures respectively. Fit indices (e.g. AIC and BIC), Lo-Mendell-Rubin likelihood ratio test, theoretical considerations, and clinical relevance will guide the decision of the optimum number of classes to represent the growth trajectories of personal recovery, disability, and community functioning. Given the heterogeneity of our sample particularly on age range and illness stages, it is important to assess measurement invariance and utilize methods (e.g., moderated nonlinear factor model [(Curran et al., 2014)](https://www.zotero.org/google-docs/?broken=wDzov8) that accommodate time-varying constructs over subsamples.

Further, we will adopt two multivariate longitudinal approaches, cross-lagged panel [(Hays et al., 1994)](https://www.zotero.org/google-docs/?broken=Ndb4YR) and parallel processes [(Wright et al., 2013)](https://www.zotero.org/google-docs/?broken=LLqNhv) models, to model the longitudinal outcome measures simultaneously. The former is a more theory based approach that can test directional effects that one variable has on another at different points in time, for example, earlier measures of functioning may predict later measures of disability. The latter could be used to associate multiple longitudinal trajectories with respect to their growth characteristics, for example, the slopes of growth trajectories of different outcomes may be associated with each other. In conjunction with growth mixture modeling under the accelerated longitudinal design, these methods could provide a more comprehensive view of the outcome measures of interest.

For Objective 2, we will associate the membership of the subgroups and thus the characteristics of these trajectories with sociodemographic and treatment factors, psychopathology, physical health, and biological measures at baseline by treating probabilistic membership as the dependent variable in the predictive models. We will apply structural equation modeling that combines the latent growth mixture model that determines the membership of the subgroups, measurement model that reduces the dimension of the predictors, and predictive model that links them into one step using simultaneous equations.

For objective 3, data analysis and outcomes are articulated in section 3.11, with further delineation taking place as part of the co-design process and pilot intervention designs.

For our exploratory objective, we will utilize correlational analyses to investigate relationships between temporal patterns emerging from topic modeling with study variables collected prospectively from study participants. We will also use general linear model analyses as appropriate to investigate differential characteristics between longitudinal outcome trajectory subtypes. These temporal patterns will also be examined as potential predictors for trajectory subtype membership in line with the methods employed for objective 2.

Overall retention rate is expected to fall within the acceptable range for statistical correction. Every effort will be made to prevent dropouts/missing data, and to complete relevant assessments for participants who drop out. Two statistical approaches, full information maximum likelihood estimation [(Arbuckle, 1996)](https://www.zotero.org/google-docs/?gONPAh) and multiple imputation methods [(Schafer, 1997)](https://www.zotero.org/google-docs/?Uh3flg) will be utilized when appropriate. As is typical in longitudinal research, we anticipate non-random missing data. Sensitivity analysis will be conducted to evaluate the impact of non-random missing data.

Sex and Gender Based Analysis

For the first two primary objectives, we will conduct additional analyses based on biological sex and gender respectively, based on data captured through the PhenX toolkit demographics measure. For Objective 1, we will determine the longitudinal functional trajectories within sex/gender groups across three domains and examine if there are substantial differences between sex/gender groups and if necessary, test Hypotheses 1a and 1b within sex/gender groups as a supplement analysis. For Objective 2, in addition to examining sex/gender as a main effect in predicting functional trajectories at the person level, we will further assess if it interacts with other sociodemographic, treatment factors, psychopathology, physical health, and biological measures in the predictive models.

Statistical Power and Sample Size

We have assessed statistical power for the following targeted analyses aimed to, a) identify distinct longitudinal functional trajectories of individuals with SSDs; b) establish predictive models to associate personal factors with growth trajectories; and c) provide feasibility indices for future interventions. Assuming that we will have approximately four distinct longitudinal trajectories, through a Monte-Carlo study we concluded that with a sample of n=1000 we have sufficient power (0.80) to correctly enumerate the classes when they are evenly distributed and reasonable power (0.68) when they are not. In the latter, we assumed that the smallest class only takes up 15% of the total sample. Further, we concluded that we have abundant power (0.91) to detect linear trend of small change over time (effect size of 0.20 between two consecutive waves) even for the smallest class (n=112). To identify associations between predictors (e.g., social determinants) and group membership of the different trajectories, we also anticipate to have sufficient power (0.82 to 0.85) for detecting a small odds ratio (OR = 1.68) depending on the distribution of the predictors. In addition, this study will provide reliable estimates for feasibility indices for future interventions. Assuming that only one fifth of the sample participate in a planned co-designed intervention, we will be able to attain a small margin of error of 6.9% for the recruitment rate, 6.0% for attrition and 6.4% for response rate. It also yields a power of 0.86 to detect a moderate effect size on a 1:1 design. For all aforementioned power estimation, we assumed 25% overall attrition and used .05 as the significance level.

Recruitment and Sampling

Recruitment of participants for this study will take place across all outpatient and inpatient clinical services at CAMH where individuals with SSDs may be receiving care, and include both new and existing patients receiving clinical services. Sequential cohorts of participants will be recruited based on duration of illness, enabling an accelerated longitudinal design for this study that will serve to maximize coverage of longitudinal functional trajectories across illness stages. Through regular monitoring of participant recruitment, we will balance recruitment across these multiple cohorts. Core research assessments will mirror measurement-based care assessments and metabolic monitoring being implemented across these clinical services, with sharing of assessment results with the patient’s clinical team. All participants will be offered the opportunity to participate in the neuroimaging and additional laboratory/biological components of this study either as part of this study, or ideally through shared participation in existing complementary studies already taking place in the Division. Harmonization of study assessments and data sharing with existing clinical research studies will enable integration and collaboration across clinical research studies in the Division, maximize recruitment efforts, and leverage existing funded research studies for data collection. With regards to the potential participant pool from which individuals could be recruited, current patient volumes across Schizophrenia Division outpatient clinical services at CAMH, for example, indicate that over 4000 unique patients were seen over the past year, with an average of over 1900 patients seen per month. Based on these volumes, and the integration of study assessments for this proposed work within the context of routine clinical care for such patients within existing clinical services at CAMH, this study will aim to recruit 500 participants per year for 2 years, yielding a total sample of 1000 participants enrolled in this study (i.e., approximately 10 participants per week from over 15 outpatient and inpatient clinical services).

**6.0 RESEARCH TEAM**

**Research Team**

This proposed study involves the collaborative effort of many scientists at CAMH, ranging from senior investigators who are international leaders in their field through to junior scientists and trainees, with complementary expertise across study domains and methodologies, and patient and family advisors. The study team members include Drs. George Foussias (study PI), Margaret Hahn, Mahavir Agarwal, Ofer Agid, Chris Bowie, Colin Hawco, David Castle, Daniel Mueller, Araba Chintoh, Vincenzo Deluca, Andreea Diaconescu, Philip Gerretsen, Ariel Graff, Yarissa Herman, Sean Hill, Omair Husain, James Kennedy, Michael Kiang, Sean Kidd, Nicole Kozloff, Marta Maslej, Farooq Naeem, Nicholas Neufeld, Gary Remington, Martin Rotenberg, Peter Selby, Ishraq Siddiqui, Arun Tiwari, Wei Wang, Joanna Yu, Clement Zai, and Robert Zipursky. We are also fortunate to have Caroline Walker and Chris Summerville as patient and family advisors, respectively, as part of our study team and study steering committee, who have contributed to the study design and selection of outcome measures for this project. At the start of this study we will also recruit additional patient and family members to join our study team in order to provide additional guidance on study implementation, and support knowledge translation activities.

Steering Committee:

Margaret Hahn, George Foussias, Chris Summerville, Caroline Walker, Mahavir Agarwal, Araba Chintoh, Nicholas Neufeld, Martin Rotenberg, Arun Tiwari, Clement Zai, Sean Kidd, Joanna Yu, Wei Wang.

Study Sections:

*Clinical symptoms and functional outcomes:*

Araba Chintoh and George Foussias (co-leads), Yarissa Herman, Chris Bowie, Philip Gerretsen, Ofer Agid, Omair Husain, Sean Kidd, Gary Remington, Peter Selby, Ishraq Siddiqui, Robert Zipursky.

*Physical health and disability:*

Mahavir Agarwal and Margaret Hahn (co-leads), Peter Selby, Robert Zipursky, Gary Remington

*Biological assessments:*

Arun Tiwari and Clement Zai (co-leads), James Kennedy, Vincenzo Deluca, Mahavir Agarwal, Margaret Hahn, Daniel Mueller

*Imaging:*

Nicholas Neufeld and Mahavir Agarwal (co-leads), Michael Kiang, Andreea Diaconescu, Colin Hawco, Ariel Graff, Philip Gerretsen

*Socio-environmental factors and health system utilization:*

Sean Kidd and Martin Rotenberg (co-leads), Nicole Kozloff, Nicholas Neufeld

*Treatment needs assessment and Pragmatic intervention co-design (future):*

Sean Kidd and Martin Rotenberg (co-leads), Mahavir Agarwal, Farooq Naeem, David Castle

*Data analysis:*

Wei Wang (lead), Joanna Yu, Marta Maslej

*EMR-based NLP-ML methodology:*

Joanna Yu and George Foussias (co-leads), Andreea Diaconescu, Marta Maslej, Sean Hill, Nicholas Neufeld, Peter Selby, Ishraq Siddiqui, Wei Wang

Research Team Qualifications:

Dr. Andreea Diaconescu is a cognitive and computational neuroscientist, with particular interest in mathematical modelling of delusions in schizophrenia. For this project, Dr. Diaconescu proposes a computational framework examining key pathologies in dopaminergic and cholinergic systems during reward and social learning, in order to predict treatment response in first episode psychosis.

Dr. Araba Chintoh is a psychiatrist and Clinician-Scientist in the Schizophrenia Division at CAMH. Her research focuses on psychopharmacology, treatment refractory schizophrenia, and psychotropic medication side effects. In addition, she has clinical expertise in treatment refractory populations with schizophrenia spectrum disorders across inpatient and outpatient clinical services.

Dr. Ariel Graff-Guerrero, MD, PhD, Clinician-scientist at CAMH and Associate Professor of Psychiatry at the University of Toronto. He has ample experience in the imaging of patients with severe neuropsychiatric disorders, including persons during prodromal psychotic symptoms, treatment-resistant schizophrenia and schizophrenia during older life. He has conducted multiple PET, MRI and MRS studies with difficult to scan patient populations. He will provide support for implementation of the study and analysis of the imaging data.

Dr. Arun Tiwari is an Assistant Professor in the Department of Psychiatry at the University of Toronto, and a Project Scientist at the Tanenbaum Centre at CAMH. His research focuses on leveraging publicly available datasets to identify genetic factors that influence response to antidepressant and antipsychotic medications, including experience of adverse side effects. He has considerable experience employing genome-wide methods that integrate genetics, epigenetics and transcriptomics for identifying predictors of treatment response. Dr. Tiwari is an author on more than 125 peer-reviewed publications and is an investigator on several provincial and federal operating grants.

Caroline Walker had her first episode of psychosis in 2008 and another followed in 2009, spending ten months in those two years as an inpatient at CAMH, often leaving psychiatrists stumped as to how to treat her. Caroline's mental health advocacy started with being invited to speak by the Dean of Students in the first inaugural Minding Our Minds conference on student mental health, which was inspired in part by watching Caroline, a bright student, succumb to an illness that left her incoherent for a long time, interrupting her academic progress through no fault of her own. This speaking role was followed by others and work as a spokesperson for CAMH in many capacities after her discharge as an inpatient in April 2010, starting with being the face of Gifts of Light the following holiday season. More recently, Caroline earned her Master of Information degree from the University of Toronto in 2018 and has embarked on a career as a Librarian & Information Professional with the eventual goal of applying her professional skills to mental health information.

Chris Summerville has been involved in many mental health initiatives as an advocate for nearly 30 years. Both as a person with lived experience with mental illness and as a family member, he has been a passionate supporter of recovery-oriented mental health services. Since 2007 Chris has been the CEO of the Schizophrenia Society of Canada and served as CEO of the Manitoba Schizophrenia Society 25 years (1993-2020).

Dr. Christopher Bowie is a Professor and the Director of Clinical Training in the Department of Psychology, and a member of the Psychiatry Department and Centre for Neuroscience Studies, at Queen’s University in Kingston, Ontario. He is the Head Consulting Psychologist at the Early Psychosis Intervention Program in Kingston and a Clinician Scientist at the Centre for Addiction and Mental Health in Toronto. His research interests focus on determining the causes and correlates, and developing treatments for, cognitive deficits in mental disorders such as schizophrenia and mood disorders.

Dr. Clement Zai completed his MSc in Medical Biophysics generating a transgenic mouse model of colorectal cancer. He completed his PhD in Medical Science conducting genetics and pharmacogenetics of schizophrenia. He became a project scientist at the Centre for Addiction and Mental Health (CAMH) and Assistant Professor at the University of Toronto in 2015. His main research goal is to elucidate the mechanism of psychiatric disorders and suicidal behaviour. His multidisciplinary research strategies (from high-throughput genetic analyses, animal behavioral models, to interactions with social and environmental factors) will yield findings that can potentially be translated to suicide prevention, reduced medication side effects, and better outcome for psychiatric patients.

Dr Colin Hawco is a cognitive neuroscientist specializing in functional brain imaging in schizophrenia. He is an Independent Scientist at CAMH and Assistant Professor at the University of Toronto. He is principal or co-investigator on several large scale neuroimaging studies on clinical populations. He will oversee the development of Imaging SOPs, staff training, and implementing standardized neuroimaging processing pipelines. Dr. Farooq Naeem is a Clinician Scientist in the Schizophrenia Division at CAMH, and Professor of Psychiatry at the University of Toronto. Dr. Naeem is also a Cognitive Behavioural Therapist. In addition to cognitive-behavioural therapy (CBT) for common mental health disorders, he received training in CBT for psychosis from Professor David Kingdon while working as his lecturer during training. He has pioneered techniques for culturally adapting CBT. He will support the psycho social intervention part of the project.

Dr. Daniel Mueller is a Clinician Scientist and a Professor at the Department of Psychiatry at the University of Toronto. He is a leading expert in psychiatric genetics and pharmacogenetics aiming to achieve better symptom remission and prevent adverse events from psychiatric medications. He has published more than 250 articles with focus on personalized medicine and has received several prestigious awards in this emerging and important field. His publications also address clinical implementation of pharmacogenetic testing including expert guidelines in collaboration with the Clinical Pharmacogenomics Implementation Consortium (CPIC) and the International Society of Psychiatric Genetics (ISPG).

Dr. Gary Remington is Senior Scientist in the Campbell Family Mental Health Research Institute and the Schizophrenia Division at CAMH. He is also Professor of Psychiatry at the University of Toronto. Dr. Remington’s research focus is in the pharmacotherapy of schizophrenia, particularly as it applies to the pharmacology and clinical profile of new antipsychotics and decision-making in antipsychotic therapy. His contributions to this project are schizophrenia; psychopharmacology; treatment response and outcome.

Dr. George Foussias is a Clinician-Scientist, Chief of the Schizophrenia Division, and Director of the Slaight Family Centre for Youth in Transition at CAMH. He is also Associate Professor in the Department of Psychiatry and the Institute of Medical Science at the University of Toronto. He heads the Virtual Reality and Behavioural Neuroscience Research Laboratory at CAMH. Dr. Foussias' research focuses on advancing the understanding of motivational deficits, and related negative and cognitive symptoms, that contribute to functional disability for individuals with schizophrenia spectrum disorders, and the development of novel digital assessment and intervention strategies to improve functional outcomes for affected individuals.

Dr. James L. Kennedy is Head of Molecular Science and Head of the Tanenbaum Centre for Pharmacogenetics in the Campbell Family Mental Health Research Institute at CAMH. He is Professor in the Department of Psychiatry and Institute of Medical Science at the University of Toronto. Dr. Kennedy's research is dedicated to finding genes involved in the cause of mental illness. He has published pioneering findings relating gene variants in the dopamine, serotonin, and neurodevelopment systems to psychiatric disorders and to treatment response.

Dr. Joanna Yu (PhD) is the Senior Portfolio Manager for the CAMH BrainHealth Databank Initiative led by the Krembil Centre for Neuroinformatics. Dr. Yu leads the team working to integrate research and care by providing advanced informatics tools to monitor patient outcomes, track trajectories, and provide personalized clinical decision support. She is experienced in data management best practices essential to ensuring collection of high quality data that can be reused for years to come. She will be contributing to this study’s multimodal data collection, integration, management, QA/QC, visualization, and sharing plans.

Dr. Mahavir Agarwal is a Clinician-Scientist and Medical Head of Clinical Research in the Schizophrenia Division and Assistant Professor in the Department of Psychiatry at the University of Toronto. His research interest lies in understanding the mechanisms underlying the interaction between schizophrenia pathophysiology, antipsychotics, cognition, and metabolic abnormalities with specific focus on the insulin signaling in the brain. He is a part of the Mental Health and Metabolism Clinic and the Medication Assessment Program for Schizophrenia (MAPS)/Clozapine Clinic at CAMH. He has received grant funding from CIHR, PSI Foundation, Ontario, and the Department of Science and Technology, Govt. of India.

Dr. Margaret Hahn is a clinician-scientist at CAMH, and an Associate Professor at the UofT, in the Department of Psychiatry. Her research interests lie in translational work focused on the complex interplay between severe mental illness (SMI), antipsychotic treatments, illness psychopathology and cardiometabolic risk, with a special interest in type 2 diabetes. She is the director of the Mental Health and Metabolic Clinic at the CAMH, which specializes in metabolic monitoring, and interventions for metabolic risk factors in individuals with serious mental illness. Dr. Hahn holds the Meighen Family Research Chair in Psychosis Prevention at UofT/CAMH, and the Cardy Schizophrenia Research Chair at CAMH. Dr. Hahn has also contributed to development of national clinical treatment guidelines in patients with comorbid SMI and obesity.

Dr. Martin Rotenberg is a Research Fellow at CAMH and the Department of Psychiatry, University of Toronto. Dr. Rotenberg is currently a psychiatrist in the Complex Care and Recovery Program at CAMH. His research focuses on the role of social and environmental factors as risk factors for developing schizophrenia spectrum disorders and how these factors impact access to care and outcomes. He also has an interest in the application and development of rehabilitation and recovery-oriented interventions. As a Co-Investigator in this study he will contribute to the measurement and examination of social determinants of health and health service use in the cohort, and the implementation and evaluation of co-design interventions.

Dr. Michael Kiang is a Clinician-Scientist who has received grant support from CIHR, SSHRC, NARSAD, the Ontario Mental Health Foundation and the American Psychiatric Institute for Research and Education, He is an expert in using neuroimaging methods, including electroencephalographic event-related potentials, to elucidate the neurocognitive mechanisms of symptoms in schizophrenia and related disorders.

Dr. Nicholas Neufeld is a practicing psychiatrist and specialist in imaging in the context of clinical research. He completed his research fellowship with Dr. Aristotle Voineskos at CAMH and is a new Assistant Professor at the University of Toronto. Dr. Neufeld co-leads the Imaging Section of PREDICTS. He will also contribute to the EMR-based NLP-ML methodology, socio-environmental factors and health system utilization in PREDICTS.

Dr. Nicole Kozloff, MD, SM, FRCPC is a psychiatrist and clinician-scientist and Associate Director of the Slaight Family Centre for Youth in Transition. Her research aims to 1) identify patterns of health service use among young people with serious mental illness in order to identify gaps in access and engagement, 2) improve the quality and delivery of mental health services to young people with serious mental illness, including through the leveraging of digital tools. She will contribute to the examination of health service utilization in PREDICTS and the development and testing of interventions to improve functional outcomes in this population.

Dr. Ofer Agid is staff psychiatrist and a clinician scientist in the Schizophrenia Program at the Centre for Addiction and Mental Health in Toronto. Dr. Agid is the Medical Head, Ambulatory Services and the Lead Psychiatrist, Partial Hospital Program at the Schizophrenia Program. He is also Associate Professor in the Department of Psychiatry, Faculty of Medicine, University of Toronto and Associate Member, Institute of Medical Science (IMS), University of Toronto. Dr. Agid’s research focus is psychopharmacology in schizophrenia, specifically early response to antipsychotic medications, early predictors of response and treatment resistant schizophrenia. His work has uncovered a new “early onset of action” hypothesis of antipsychotics, which has impacted current practice guidelines for the treatment of schizophrenia.

Dr. Peter Selby is a Clinician Scientist and Senior Medical Consultant at CAMH. He is a Professor in the Departments of Family and Community Medicine, Psychiatry, and the Dalla Lana School of Public Health at the University of Toronto. He is also a Clinician Scientist in the Department of Family and Community Medicine. Dr. Selby's research focus is on innovative methods to understand and treat addictive behaviours and their comorbidities. He also uses technology to combine clinical medicine and public health methods to scale up and test health interventions. His cohort of 240,000 treated smokers in Ontario is an example.

Dr. Philip Gerretsen is a Clinician Scientist in the Campbell Family Mental Health Research Institute and Geriatric Mental Health Services at CAMH. He is an Assistant Professor in the Department of Psychiatry in the Faculty of Medicine at the University of Toronto. His expertise is in insight into illness or subjective illness awareness. Impaired illness awareness is a well-recognized, but understudied phenomenon that contributes to minimization and denial of the need for treatment, with devastating clinical and social consequences, particularly in schizophrenia spectrum disorders. Dr. Gerretsen’s research has focused on the identification of the neural correlates of impaired illness awareness in schizophrenia, primarily through structural and functional neuroimaging. The results have provided prospective biomarkers and regional brain targets for intervention psychotherapeutically and with non-invasive neurostimulation, such as transcranial direct current, magnetic and vestibular stimulation. The relevance of this work lies in the potential to alter individuals’ attitude toward their illness and treatment. This ultimately would lead to an improvement in individuals’ capacity for illness recognition and engagement in treatment, which would have a significant impact on disease management. Dr. Gerretsen will bring his expertise in this area to the project.

Dr. Robert Zipursky is a psychiatrist and clinician-scientist, and Professor of Psychiatry with expertise in the study of early intervention for schizophrenia. His research contributions have been in the areas of structural and functional brain imaging, antipsychotic treatment response, characterization of clinical and functional outcomes, and in the assessment of patient preferences.

Dr. Sean Hill is the Director of the Krembil Centre for Neuroinformatics, Senior Scientist at the Centre for Addiction and Mental Health (CAMH), and Professor at the University of Toronto. Dr. Hill is a computational neuroscientist and applies large-scale data integration, neuroinformatics, multiscale brain modeling and machine learning to improve our understanding and treatment of mental health disorders.

Dr. Sean Kidd is a Clinical Psychologist, Senior Scientist, and the Division Chief of Psychology at the Centre for Addiction and Mental Health and an Associate Professor in the University of Toronto Department of Psychiatry. Dr. Kidd's research has included the study of models of peer support, cognitive interventions for schizophrenia, and complex interventions for homeless youth, and mobile health strategies for psychosis. As a Co-I on this study he will contribute to both social determinants aspects of the cohort study as well as pilot clinical trial development.

Dr. Vincenzo De Luca is a Psychiatrist and neuroscience researcher interested in: 1) the contribution of genetics to the risk of developing psychiatric conditions, particularly schizophrenia; 2) the identification of predictors of antipsychotic treatment response (clinical, genetic, epigenetic and their interaction); and 3) Statistical approaches in the epigenetic analysis of neuropsychiatric diseases.

Dr. Wei Wang, independent scientist/Biostatistician at CAMH, is an expert on complex trial design, longitudinal modelling and integrative data analysis. He works closely with the study PIs and section leads on study design, data analysis and dissemination of study findings.

Dr. Yarissa Herman is a clinical psychologist registered with the College of Psychologists of Ontario. Her doctoral research explored the relationship between cognitive functioning and outcomes in addiction treatment. Dr. Herman is currently a psychologist in the Complex Care and Recovery Program at the Centre for Addiction and Mental Health (CAMH). The focus of her clinical and research work is the development, implementation, and evaluation of evidence-based interventions for individuals with severe mental illness and concurrent substance use disorders. Dr. Herman provides evidence-based interventions for a variety of difficulties including addiction, psychosis, anxiety, and depression. She works with both adults and adolescents.

**7.0 Risks and Benefits**

**7.1 Potential Risks and Benefits to the Participants**

The clinical assessments utilized in this study are physically non-invasive assessments associated with minimal risk. All measures have been used in prior studies with participants with mental illness and have been found to elicit minimal distress or discomfort. That said, given that some of the assessment questions are of personal nature, some individuals may be uncomfortable answering them. Patients are informed that they may refuse to answer any questions and may ask to stop at any time. Further, some participants may find the cognitive measures to be long, tiresome and frustrating at times. Measures, such as scheduled breaks, are taken to encourage participants to remain engaged. Participants are also made aware that they are free to withdraw from the study at any time, with no consequences. Lastly, the utilization of data gathered in the context of routine clinical care is intended to reduce the repetition of assessments for participants, decrease study visit duration, and minimize participant burden.

There will be minimal direct benefits to participants enrolling in this study.

With participant consent/assent, a summary of study results can be shared with the participant’s clinicians, providing additional information which may be of value to future clinical encounters and which will be available in the CAMH medical record.

**7.2 Risks Associated with the MRI examination**

There are four categories of risks to human subjects directly associated with MRI examination, including:

1. Acoustic Noise Levels: The risk associated with acoustic noise levels are related to the noise generated by the pulsing of the gradients. The risk to the participant may be temporary loss of hearing. The easiest and most reliable means of preventing hearing loss is to use disposable earplugs. Disposable earplugs or non-magnetic headphone sets will be used for all participants in this study.

1. Static Magnetic Fields: There are no established risks of exposure to magnetic fields of 3T, other than the incidental risks (listed below). Like other clinical MRI centers, our facilities incorporate a complete range of procedures to assure security of the restricted access area, and careful screening of potential participants, before they enter the restricted access area.

1. Gradient or Time-Varying Magnetic Fields: Risks associated with gradient magnetic fields are related to dB/dt (change in magnetic field over time), which can induce electric fields and currents in conductive media, including biological tissue. The MRI systems will be operated within limits already determined not to pose significant risk to humans.

1. Specific Absorption Rate (SAR): The risks associated with Specific Absorption Rate (SAR) are related to the fact that given a large enough SAR, heating of tissue may occur. Proper and routine monitoring of all RF electronics (e.g. coils, transmitters, system security, etc.) will be performed on a regular basis. There are three categories of incidental risks to all human subjects in a restricted access area. The three categories of concern are related to: (1) malfunctioning or movement of implanted metal objects (i.e., aneurysm clips, pacemakers, etc.); (2) injury from a projectile (i.e., ferromagnetic objects being attracted into the magnet); and (3) asphyxiation due to large amounts of cryogenic gasses generated during a quench (i.e., the event that occurs when a magnet makes the sudden transition from superconducting to resistively conducting). These categories of incidental risk are unlikely given the extensive set of safety checks employed by our facilities prior to scanning

Risks During Pregnancy: The risks during pregnancy are unknown. Several studies of prenatal MRI have now been published and the MRI techniques have not been demonstrated thus far to have risks. There have been no reports of adverse effects to the developing human fetus. However, because the risks have not been adequately assessed over a long period of time, women who have a confirmed pregnancy or a reason to believe they may be pregnant, as documented on the MRI Safety Screening Form, will be excluded from the MRI scan.

Risks of Psychological Distress: Some participants can experience claustrophobia while in the scanner. Participants will have the opportunity to examine this space before the scanning starts and participate in a session with a mock scanner. This study will not offer alternative procedures for participants who choose not to participate in the MRI scan or who are excluded. In our experience of scanning a large number of participants with severe mental disorders across the lifespan (e.g. autism, schizophrenia, bipolar disorder, major depressive disorder), less than 2% of these participants have been unable to complete the scanning session. However, mild psychological distress is likely to occur during the course of the conduct of the neuroimaging studies.

Risk of Incidental Findings: The possibility of unexpected or incidental findings carries with it some risks. Research scans are not designed to be used for diagnosis. In the unlikely event an atypical finding is seen on a participant’s MRI scan, a study investigator may ask a radiologist or other qualified health professional to look at the participant’s scan.

**7.3 Risks Associated with Blood Collection**

Risk of Bruising and Discomfort: Blood collection may cause some bruising and discomfort at the site of needle stick, and rarely, a small infection at the skin puncture site. These risks are minimized by using proper techniques.

Risk of Fainting: As with any blood draw, some participants may suddenly begin to feel faint or lose consciousness at the sight of a needle or blood. To avoid any adverse effects or injury, researchers will ensure that all participants are comfortably seated during the blood draw. Each participant will be asked to report any history of fainting during previous injections or blood draws. Those with a known history of fainting will be offered the option to lie down during the blood draw. All blood draws will be conducted by trained professionals (nurses, phlebotomists), who will monitor for signs of discomfort in the participant (e.g., sudden paleness, sweating excessively, distress) in order to avoid full loss of consciousness whenever possible. In the event that the participant does not feel well, the blood draw will be terminated immediately and the team will ensure the participant rests comfortably until they are ready to leave the lab.

**7.4 Risks Associated with Virtual Assessments**

There are some privacy and security risks associated with videoconferencing, as the information could be intercepted by unauthorized people or otherwise shared by accident. The strict precautions that will be taken by research staff to protect privacy include using only CAMH-approved video conferencing tools (i.e. Cisco WebEx), ensuring all assessment administrators are in a private space and ensuring that no private or confidential information is displayed on the administrator’s device or within their workspace prior to initiating a remote assessment. Prior to the video conference session, the participant will be advised of the ways that they can assist in maintaining privacy including making sure they are in a quiet, private place for the duration of the assessment, using a personal device instead of shared or work device, ensuring they are on a password-protected private WiFi network, and using earbuds or headphones. If the administration of remote assessments requires the study staff to maintain paper records outside of CAMH, all requirements of CAMH SOP GR106: Working Remotely on Research Projects will be satisfied.

**7.5 Risks associated with accessing clinical notes from the CAMH EMR**

Analyzing clinical notes from the CAMH EMR presents some risks to patient privacy. For retrospective CAMH patients who are not formal participants in the study, free text clinical notes will be converted to features prior to being accessed by the study team, which reduces privacy risks. For study participants, although their notes will be de-identified with CRATE prior to being accessed by the study team, these notes may still contain indirect identifiers and other sensitive or personal information. However, there are safeguards in place to minimize risks related to accessing these notes, such as storing and analyzing the data via the secure CAMH Neuroinformatics platform and restricting its access to trained study team members and authorized personnel. Furthermore, study participants are informed that data from the CAMH EMR will be accessed as part of the study, and this information will only be accessed for consenting participants. Having access to the full, de-identified notes is necessary for human validation of any themes or patterns emerging from topic modelling and other automated analyses, and it is useful for developing NLP methods.

**7.6 Importance of Knowledge to Be Gained**

The risks associated with clinical assessments and the procedures for this study are similar to the risks associated with other clinical studies. The primary aim of the study is to identify the longitudinal functional trajectories for individuals with SSDs, and the clinical and biological predictors of these trajectories. Through this, the study has the potential to identify novel treatment targets for treatment intervention for a population of individuals who experience, to varying degrees, longstanding disability. Overall, we believe that the knowledge to be gained from this study is great with respect to the risks and discomforts potentially associated with the procedures proposed herein. Given the potential significance of the proposal the risk/benefit ratio appears to be reasonable.

**8.0 IMPACT AND INNOVATION**

At a population and health systems level, our work has the potential to identify and predict individual recovery and functional trajectories at the individual patient level, and develop and study real-world interventions to improve functional outcomes, recovery, and community functioning. This is predicted to enhance the precision and effectiveness of interventions to address disability for people with SSDs, support the development of more cost-effective service designs, and reduce the economic costs associated with SSDs.

From a neuroscience and biological perspective, we will be able to identify biological and metabolic profiles, physical health parameters, and structural and functional properties of brain circuits that are associated with, and which may predict different illness and functional trajectories.

We foresee that this work will create a very unique, multidisciplinary training platform which will attract the best and brightest trainees from diverse backgrounds. Principles of data sharing and multi-level data-driven techniques will build a spirit of collaboration and altruism across the Centre. By training the nex47t generation of investigators, we will increase the number of highly qualified personnel committed to improving the mental health of individuals across the nation and beyond.

The impact for clinical care is also immense. Research offers increased monitoring and more intensive follow-up as compared to routine clinical care. Use of measurement-based approaches, and family and patient engagement to co-design care, will provide templates for future models of care and best practice. Furthermore, this initiative will address the huge gaps in physical care that drive morbidity and premature mortality in this population. We anticipate that these efforts will open up the potential for system level change to ensure individuals are treated from all health-related (i.e. physical and mental) perspectives, also bridging medical and mental health care for individuals with SSDs.

**9.0 SUMMARY**

Despite advances in the understanding and treatment of SSDs, the hallmark of these serious and persistent mental illnesses continues to be one of enduring disability. The proposed work is anticipated to advance the early identification and prediction of functional and recovery trajectories at the individual level for people experiencing a SSD, the core contributors to these trajectories, and set the stage for targeted interventions to alter one’s illness trajectory to promote functional recovery. To this last point, the proposed study offers a unique opportunity to develop and study real-world interventions. Using participatory and co-design approaches, we will pilot pragmatic interventions which target physical health and psychosocial outcomes, and which can be moved through to randomized trials within and beyond the cohort. By establishing a core platform of clinical research in SSDs at CAMH, this work will also enable and support other focused studies in this population, and serve as a springboard for training and development of early career scientists.

**Appendix 1: Schedule of Assessments**

| **Measure** | **Informant (S**elf **R**eport**; Ca**regiver**; R**ater **A**dministered**)** | **Admin Time (min)** | **Time point (months)** | | | | | | |
| --- | --- | --- | --- | --- | --- | --- | --- | --- | --- |
|  |  |  | **0** | **6** | **12** | **18** | **24** | **30** | **36** |
| **PERSONAL RECOVERY, DISABILITY, AND FUNCTIONING** | | | | | | | | | |
| RAS | SR | 5 | • | • | • | • | • | • | • |
| WHO-DAS (36-item) | SR/Ca | 10 | • | • | • | • | • | • | • |
| PSP | RA | 5 | • | • | • | • | • | • | • |
| C-PROM | SR | 10 | • |  | • |  | • |  | • |
| WHOQOL-BREF | SR | 5 | • |  | • |  | • |  | • |
| **DEMOGRAPHICS** | | | | | | | | | |
| CAMH Health Equity Form | RA | 2 | • |  | • |  | • |  | • |
| PhenX Demographics | RA | 3 | • |  | • |  | • |  | • |
| **DIAGNOSTIC, CLINICAL, AND COGNITIVE** | | | | | | | | | |
| SCID-5 | RA | 60 | • |  |  |  |  |  |  |
| BPRS | RA | 20 | • |  | • |  | • |  | • |
| mCSI | SR | 5 | • | • | • | • | • | • | • |
| SANS | RA | 20 | • |  | • |  | • |  | • |
| CGI | RA | 5 | • |  | • |  | • |  | • |
| VAGUS | SR | 5 | • |  | • |  | • |  | • |
| DAI | SR | 3 | • |  | • |  | • |  | • |
| CSSRS | RA | 10 | • |  | • |  | • |  | • |
| AUDIT-C | SR | 3 | • |  | • |  | • |  | • |
| NIDA Quick Screen/mAssist | SR | 3 | • |  | • |  | • |  | • |
| DFAQ Inventory | SR | 10 | • |  | • |  | • |  | • |
| CAMH Tobacco Screening | SR | 2 | • |  | • |  | • |  | • |
| BACS | RA | 30 | • |  | • |  | • |  | • |
| ER-40 | RA | 10 | • |  | • |  | • |  | • |
| TASIT | RA | 15 | • |  | • |  | • |  | • |
| Medication Inventory | RA | 5 | • |  | • |  | • |  | • |
| AIMS | RA | 5 | • |  | • |  | • |  | • |
| SARS | RA | 5 | • |  | • |  | • |  | • |
| BARS | RA | 3 | • |  | • |  | • |  | • |
| S-NES | RA | 5 | • |  | • |  | • |  | • |
| SWN | SR | 5 | • |  | • |  | • |  | • |
| UKU | SR | 5 | • |  | • |  | • |  | • |
| **PHYSICAL HEALTH** | | | | | | | | | |
| Height, Weight, BMI, and Waist Circumference | RA | 5 | • | • | • | • | • | • | • |
| Laboratory Assessments^ | RA | 5 | • |  | • |  | • |  | • |
| Metabolomics Sample^ (collected as part of laboratory draw) | RA |  | • |  |  |  |  |  |  |
| Medical history | RA | 5 | • |  | • |  | • |  | • |
| SIMPAQ | SR | 5 | • |  | • |  | • |  | • |
| OAS-SF | SR | 2 | • |  | • |  | • |  | • |
| **SOCIAL, ENVIRONMENTAL, AND HEALTH SYSTEM** | | | | | | | | | |
| VIA | SR | 5 |  | • |  |  |  |  |  |
| MIEM-R | SR | 2 |  | • |  |  |  |  |  |
| PhenX Acculturation Survey | SR | 1 |  | • |  |  |  |  |  |
| EDS | SR | 5 | • |  | • |  | • |  | • |
| ISMI | SR | 5 | • |  | • |  | • |  | • |
| LEC | SR | 2 |  | • |  | • |  | • |  |
| CTQ-SF | SR | 5 |  | • |  |  |  |  |  |
| CD-RISC | SR | 2 |  | • |  | • |  | • |  |
| SERS-SF | SR | 5 | • |  | • |  | • |  | • |
| MSPSS | SR | 3 | • |  | • |  | • |  | • |
| Postal Code | SR | - | • |  | • |  | • |  | • |
| SASCAT | RA | 15 | • |  |  |  |  |  |  |
| VTLFB | RA | 10 | • |  | • |  | • |  | • |
| RTLFB | RA | 15 | • |  | • |  | • |  | • |
| HSJSU | RA | 10 | • |  | • |  | • |  | • |
| **BIOLOGICAL** | | | | | | | | | |
| Genetics samples | RA | 2 | • |  | • |  | • |  | • |
| MRI | RA | 60 | • |  |  |  |  |  |  |

**Legend***: Recovery Assessment Scale (RAS); World Health Organization Disability Assessment Schedule 2.0 (WHO-DAS); Personal and Social Performance (PSP); Canadian Personal Recovery Outcome Measure (C-PROM); WHO Quality Of Life - Brief Version (WHOQOL-BREF); Structured Clinical Interview for DSM-5 (SCID-5); Brief Psychiatric Rating Scale (BPRS); Modified Colorado Symptom Index (mCSI); Scale for the Assessment of Negative Symptoms (SANS); Clinical Global Impression Scale (CGI); VAGUS Insight Into Psychosis Scale (VAGUS); Drug Attitude Inventory (DAI); Columbia Suicide Severity Rating Scale (CSSRS); Alcohol Use Disorders Identification Test - Consumption (AUDIT-C); Brief Assessment of Cognition in Schizophrenia (BACS); DFAQ-CU; Daily Session, Frequency, Age of Onset and Quantity of Cannabis Use Inventory, Penn Emotion Recognition task (ER-40); The Awareness of Social Inference Test (TASIT); Abnormal Involuntary Movement Scale (AIMS); Simpson Angus Rating Scale (SARS); Barnes Akathisia Rating Scale (BARS); Short Neurological Assessment Scale (S-NES); The self-report Subjective Well-being under Neuroleptics Scale (SWN); Side Effect Rating Scale (UKU); Simple Physical Activity Questionnaire (SIMPAQ); Obesity Adjustment Survey Short Form (OAS-SF); Vancouver Index of Acculturation (VIA); Multi-Group Ethnic Identity-Revised (MIEM-R); Everyday Discrimination Scale (EDS); Internalized Stigma of Mental Illness (ISMI); Life Events Checklist (LEC); Childhood Trauma Questionnaire – Short Form (CTQ-SF); Connor-Davidson Resilience Scale (CD-RISC); Self-Esteem Rating Scale-Short Form (SERS-SF); Multidimensional Scale of Perceived Social Support (MSPSS); Short Adapted Social Capital Assessment Tool (SASCAT); Vocational Time-Line Follow-Back (VTLFB);* Residential Timeline Follow-Back (RTLFB); *The Health, Social and Justice Service Use Inventory (HSJSU)*

**Reference List**

[Addington, J, & Addington, D. (1993). Premorbid functioning, cognitive functioning, symptoms and outcome in schizophrenia. *Journal of Psychiatry and Neuroscience*, *18*(1), 18–23.](https://www.zotero.org/google-docs/?SJrRhI)

[Addington, J, & Addington, D. (1999). Neurocognitive and social functioning in schizophrenia. *Schizophrenia Bulletin*, *25*(1), 173–182.](https://www.zotero.org/google-docs/?SJrRhI)

[Addington, J, & Addington, D. (2000). Neurocognitive and social functioning in schizophrenia: A 2.5 year follow-up study. *Schizophrenia Research*, *44*(1), 47–56.](https://www.zotero.org/google-docs/?SJrRhI)

[Addington, Jean, & Addington, D. (2008). Social and cognitive functioning in psychosis. *Schizophrenia Research*, *99*(1–3), 176–181. https://doi.org/10.1016/j.schres.2007.07.004](https://www.zotero.org/google-docs/?SJrRhI)

[Addington, Jean, Girard, T. A., Christensen, B. K., & Addington, D. (2010). Social cognition mediates illness-related and cognitive influences on social function in patients with schizophrenia-spectrum disorders. *Journal of Psychiatry & Neuroscience: JPN*, *35*(1), 49–54. https://doi.org/10.1503/jpn.080039](https://www.zotero.org/google-docs/?SJrRhI)

[Adekkanattu, P., Sholle, E. T., DeFerio, J., Pathak, J., Johnson, S. B., & Campion, T. R. (2018). Ascertaining Depression Severity by Extracting Patient Health Questionnaire-9 (PHQ-9) Scores from Clinical Notes. *AMIA Annu Symp Proc*, *2018*, 147–156.](https://www.zotero.org/google-docs/?SJrRhI)

[Adriano, F., Caltagirone, C., & Spalletta, G. (2012). Hippocampal volume reduction in first-episode and chronic schizophrenia: A review and meta-analysis. *Neuroscientist*, *18*(2), 180–200. https://doi.org/10.1177/1073858410395147](https://www.zotero.org/google-docs/?SJrRhI)

[Agid, O., Arenovich, T., Sajeev, G., Zipursky, R. B., Kapur, S., Foussias, G., & Remington, G. (2011). An algorithm-based approach to first-episode schizophrenia: Response rates over 3 prospective antipsychotic trials with a retrospective data analysis. *The Journal of Clinical Psychiatry*, *72*(11), 1439–1444. https://doi.org/10.4088/JCP.09m05785yel](https://www.zotero.org/google-docs/?SJrRhI)

[Agid, O., McDonald, K., Siu, C., Tsoutsoulas, C., Wass, C., Zipursky, R. B., Foussias, G., & Remington, G. (2012). Happiness in first-episode schizophrenia. *Schizophrenia Research*, *141*(1), 98–103. https://doi.org/10.1016/j.schres.2012.07.012](https://www.zotero.org/google-docs/?SJrRhI)

[Ahmed, A. O., Strauss, G. P., Buchanan, R. W., Kirkpatrick, B., & Carpenter, W. T. (2018). Schizophrenia heterogeneity revisited: Clinical, cognitive, and psychosocial correlates of statistically-derived negative symptoms subgroups. *Journal of Psychiatric Research*, *97*, 8–15. https://doi.org/10.1016/j.jpsychires.2017.11.004](https://www.zotero.org/google-docs/?SJrRhI)

[Alameda, L., Ferrari, C., Baumann, P., Gholam, M., Do, K., & Conus, P. (2015). Childhood sexual and physical abuse: Age at exposure modulates impact on functional outcome in early psychosis patients. *Psychological Medicine*, *45*, 2727–2736. https://doi.org/10.1017/S0033291715000690](https://www.zotero.org/google-docs/?SJrRhI)

[Albert, N., Bertelsen, M., Thorup, A., Petersen, L., Jeppesen, P., Le Quack, P., Krarup, G., Jørgensen, P., & Nordentoft, M. (2011). Predictors of recovery from psychosis: Analyses of clinical and social factors associated with recovery among patients with first-episode psychosis after 5years. *Schizophrenia Research*, *125*(2), 257–266. https://doi.org/10.1016/j.schres.2010.10.013](https://www.zotero.org/google-docs/?SJrRhI)

[Alessandrini, M., Lançon, C., Fond, G., Faget-Agius, C., Richieri, R., Faugere, M., Metairie, E., Boucekine, M., Llorca, P.-M., Auquier, P., & Boyer, L. (2016). A structural equation modelling approach to explore the determinants of quality of life in schizophrenia. *Schizophrenia Research*, *171*(1), 27–34. https://doi.org/10.1016/j.schres.2016.01.012](https://www.zotero.org/google-docs/?SJrRhI)

[Ali, S. A., Mathur, N., Malhotra, A. K., & Braga, R. J. (2019). Electroconvulsive Therapy and Schizophrenia: A Systematic Review. *Molecular Neuropsychiatry*, *5*(2), 75–83. https://doi.org/10.1159/000497376](https://www.zotero.org/google-docs/?SJrRhI)

[Alley, D. E., & Chang, V. W. (2007). The changing relationship of obesity and disability, 1988-2004. *Journal of the American Medical Association*, *298*(17). https://doi.org/10.1001/jama.298.17.2020](https://www.zotero.org/google-docs/?SJrRhI)

[Allison, D. B., Mackell, J. A., & McDonnell, D. D. (2003). The impact of weight gain on quality of life among persons with schizophrenia. *Psychiatric Services*, *54*(4). https://doi.org/10.1176/appi.ps.54.4.565](https://www.zotero.org/google-docs/?SJrRhI)

[Anderson, K. K., Cheng, J., Susser, E., McKenzie, K. J., & Kurdyak, P. (2015). Incidence of psychotic disorders among first-generation immigrants and refugees in Ontario. *CMAJ*. https://doi.org/10.1503/cmaj.141420](https://www.zotero.org/google-docs/?SJrRhI)

[Anderson, K. K., Norman, R., MacDougall, A., Edwards, J., Palaniyappan, L., Lau, C., & Kurdyak, P. (2018). Effectiveness of Early Psychosis Intervention: Comparison of Service Users and Nonusers in Population-Based Health Administrative Data. *American Journal of Psychiatry*, *175*(5), 443–452. https://doi.org/10.1176/appi.ajp.2017.17050480](https://www.zotero.org/google-docs/?SJrRhI)

[Andreasen, N. C. (1982). Negative symptoms in schizophrenia. Definition and reliability. *Archives of General Psychiatry*, *39*(7), 784–788.](https://www.zotero.org/google-docs/?SJrRhI)

[Andresen, R., Caputi, P., & Oades, L. G. (2010). Do clinical outcome measures assess consumer-defined recovery? *Psychiatry Research*, *177*(3), 309–317. https://doi.org/10.1016/j.psychres.2010.02.013](https://www.zotero.org/google-docs/?SJrRhI)

[Arango, C., Garibaldi, G., & Marder, S. R. (2013). Pharmacological approaches to treating negative symptoms: A review of clinical trials. *Schizophrenia Research*, *150*(2–3), 346–352. https://doi.org/10.1016/j.schres.2013.07.026](https://www.zotero.org/google-docs/?SJrRhI)

[Arbuckle, J. (1996). Full information estimation in the presence of incomplete data. In *Full information estimation in the presence of incomplete data*. Lawrence Erlbaum Associates, Inc.](https://www.zotero.org/google-docs/?SJrRhI)

[Attkisson, C. C., & Greenfield, T. K. (1994). Client Satisfaction Questionnaire-8 and Service Satisfaction Scale-30. In *The use of psychological testing for treatment planning and outcome assessment* (pp. 402–420). Lawrence Erlbaum Associates, Inc.](https://www.zotero.org/google-docs/?SJrRhI)

[Austin, S. F., Mors, O., Secher, R. G., Hjorthøj, C. R., Albert, N., Bertelsen, M., Jensen, H., Jeppesen, P., Petersen, L., Randers, L., Thorup, A., & Nordentoft, M. (2013). Predictors of recovery in first episode psychosis: The OPUS cohort at 10 year follow-up. *Schizophrenia Research*, *150*(1), 163–168. https://doi.org/10.1016/j.schres.2013.07.031](https://www.zotero.org/google-docs/?SJrRhI)

[Awad, A. G., & Voruganti, L. N. (2008). The burden of schizophrenia on caregivers: A review. *PharmacoEconomics*, *26*(2), 149–162. https://doi.org/10.2165/00019053-200826020-00005](https://www.zotero.org/google-docs/?SJrRhI)

[Bae, S.-M., Lee, S.-H., Park, Y.-M., Hyun, M.-H., & Yoon, H. (2010). Predictive Factors of Social Functioning in Patients with Schizophrenia: Exploration for the Best Combination of Variables Using Data Mining. *Psychiatry Investigation*, *7*(2), 93–101. https://doi.org/10.4306/pi.2010.7.2.93](https://www.zotero.org/google-docs/?SJrRhI)

[Ballon, J. S., Pajvani, U., Freyberg, Z., Leibel, R. L., & Lieberman, J. A. (2014). Molecular pathophysiology of metabolic effects of antipsychotic medications. *Trends Endocrinol Metab*, *25*(11), 593–600. https://doi.org/10.1016/j.tem.2014.07.004](https://www.zotero.org/google-docs/?SJrRhI)

[Barbic, S. P., Kidd, S. A., Durisko, Z. T., Yachouh, R., Rathitharan, G., & McKenzie, K. (2018). What Are the Personal Recovery Needs of Community-Dwelling Individuals with Mental Illness? Preliminary Findings from the Canadian Personal Recovery Outcome Measurement (C-PROM) Study. *Canadian Journal of Community Mental Health*. https://doi.org/10.7870/cjcmh-2018-005](https://www.zotero.org/google-docs/?SJrRhI)

[Barkhuizen, W., Cullen, A. E., Shetty, H., Pritchard, M., Stewart, R., McGuire, P., & Patel, R. (2020). Community treatment orders and associations with readmission rates and duration of psychiatric hospital admission: A controlled electronic case register study. *BMJ Open*, *10*(3), e035121. https://doi.org/10.1136/bmjopen-2019-035121](https://www.zotero.org/google-docs/?SJrRhI)

[Barnes, T. R. (1989). A rating scale for drug-induced akathisia. *Br J Psychiatry*, *154*, 672–676.](https://www.zotero.org/google-docs/?SJrRhI)

[Barrett, N. M., Gill, K. J., Pratt, C. W., & Roberts, M. M. (2013). *Psychiatric Rehabilitation*. Academic Press.](https://www.zotero.org/google-docs/?SJrRhI)

[Bartoli, F., Crocamo, C., Di Brita, C., Esposito, G., Tabacchi, T. I., Verrengia, E., Clerici, M., & Carrà, G. (2019). Adjunctive second-generation antipsychotics for specific symptom domains of schizophrenia resistant to clozapine: A meta-analysis. *Journal of Psychiatric Research*, *108*, 24–33. https://doi.org/10.1016/j.jpsychires.2018.11.005](https://www.zotero.org/google-docs/?SJrRhI)

[Bate, P., & Robert, G. (2006). Experience-based design: From redesigning the system around the patient to co-designing services with the patient. *Quality & Safety in Health Care*, *15*(5), 307–310. https://doi.org/10.1136/qshc.2005.016527](https://www.zotero.org/google-docs/?SJrRhI)

[Baumann, P., Hiemke, C., Ulrich, S., Eckermann, G., Gaertner, I., Gerlach, M., Kuss, H.-J., Laux, G., Müller-Oerlinghausen, B., Rao, M. L., Riederer, P., Zernig, G., & Arbeitsge-meinschaft fur neuropsychopharmakologie und pharmakopsychiatrie. (2004). The AGNP-TDM expert group consensus guidelines: Therapeutic drug monitoring in psychiatry. *Pharmacopsychiatry*, *37*(6), 243–265. https://doi.org/10.1055/s-2004-832687](https://www.zotero.org/google-docs/?SJrRhI)

[Behdinan, T., Foussias, G., Wheeler, A. L., Stefanik, L., Felsky, D., Remington, G., Rajji, T. K., Mallar Chakravarty, M., & Voineskos, A. N. (2015). Neuroimaging predictors of functional outcomes in schizophrenia at baseline and 6-month follow-up. *Schizophrenia Research*, *169*(1–3), 69–75. https://doi.org/10.1016/j.schres.2015.10.023](https://www.zotero.org/google-docs/?SJrRhI)

[Ben-Zeev, D., Razzano, L. A., Pashka, N. J., & Levin, C. E. (2021). Cost of mHealth Versus Clinic-Based Care for Serious Mental Illness: Same Effects, Half the Price Tag. *Psychiatr Serv*, appips202000349. https://doi.org/10.1176/appi.ps.202000349](https://www.zotero.org/google-docs/?SJrRhI)

[Bernstein, D. P., Ahluvalia, T., Pogge, D., & Handelsman, L. (1997). Validity of the Childhood Trauma Questionnaire in an adolescent psychiatric population. *Journal of the American Academy of Child & Adolescent Psychiatry*, *36*(3), 340–348. https://doi.org/10.1097/00004583-199703000-00012](https://www.zotero.org/google-docs/?SJrRhI)

[Best, M. W. (2020). *CIHR KNOWLEDGE SYNTHESIS: EXAMINING THE EFFICACY OF EVIDENCE-BASED PSYCHOSOCIAL INTERVENTIONS FOR SCHIZOPHRENIA-SPECTRUM DISORDERS DELIVERED THROUGH VIRTUAL CARE*. 31.](https://www.zotero.org/google-docs/?SJrRhI)

[Best, M. W., Law, H., Pyle, M., & Morrison, A. P. (2020). Relationships between psychiatric symptoms, functioning and personal recovery in psychosis. *Schizophr Res*, *223*, 112–118. https://doi.org/10.1016/j.schres.2020.06.026](https://www.zotero.org/google-docs/?SJrRhI)

[Bhagyavathi, H. D., Mehta, U. M., Thirthalli, J., Kumar, C. N., Kumar, J. K., Subbakrishna, D. K., & Gangadhar, B. N. (2015). Cascading and combined effects of cognitive deficits and residual symptoms on functional outcome in schizophrenia – A path-analytical approach. *Psychiatry Research*, *229*(1), 264–271. https://doi.org/10.1016/j.psychres.2015.07.022](https://www.zotero.org/google-docs/?SJrRhI)

[Biessels, G. J., Staekenborg, S., Brunner, E., Brayne, C., & Scheltens, P. (2006). *Risk of dementia in diabetes mellitus: A systematic review*.](https://www.zotero.org/google-docs/?SJrRhI)

[Bighelli, I., Huhn, M., Schneider-Thoma, J., Krause, M., Reitmeir, C., Wallis, S., Schwermann, F., Pitschel-Walz, G., Barbui, C., Furukawa, T. A., & Leucht, S. (2018). Response rates in patients with schizophrenia and positive symptoms receiving cognitive behavioural therapy: A systematic review and single-group meta-analysis. *BMC Psychiatry*, *18*(1), 380. https://doi.org/10.1186/s12888-018-1964-8](https://www.zotero.org/google-docs/?SJrRhI)

[Bilic, P., Jukic, V., Vilibic, M., Savic, A., & Bozina, N. (2014). Treatment-resistant schizophrenia and DAT and SERT polymorphisms. *Gene*, *543*(1), 125–132. https://doi.org/10.1016/j.gene.2014.03.050](https://www.zotero.org/google-docs/?SJrRhI)

[Birchwood, M., Smith, J., Cochrane, R., Wetton, S., & Copestake, S. (1990). The Social Functioning Scale. The development and validation of a new scale of social adjustment for use in family intervention programmes with schizophrenic patients. *The British Journal of Psychiatry : The Journal of Mental Science*, *157*, 853–859.](https://www.zotero.org/google-docs/?SJrRhI)

[Blanchard, J. J., Bradshaw, K. R., Garcia, C. P., Nasrallah, H. A., Harvey, P. D., Casey, D., Csoboth, C. T., Hudson, J. I., Julian, L., Lentz, E., Nuechterlein, K. H., Perkins, D. O., Skale, T. G., Snowden, L. R., Tandon, R., Tek, C., Velligan, D., Vinogradov, S., & O’Gorman, C. (2017). Examining the reliability and validity of the Clinical Assessment Interview for Negative Symptoms within the Management of Schizophrenia in Clinical Practice (MOSAIC) multisite national study. *Schizophrenia Research*, *185*, 137–143. https://doi.org/10.1016/j.schres.2017.01.011](https://www.zotero.org/google-docs/?SJrRhI)

[Blanchard, J. J., Horan, W. P., & Collins, L. M. (2005). Examining the latent structure of negative symptoms: Is there a distinct subtype of negative symptom schizophrenia? *Schizophrenia Research*, *77*(2), 151–165. https://doi.org/10.1016/j.schres.2005.03.022](https://www.zotero.org/google-docs/?SJrRhI)

[Bobes, J., Arango, C., Garcia-Garcia, M., & Rejas, J. (2009). Prevalence of Negative Symptoms in Outpatients With Schizophrenia Spectrum Disorders Treated With Antipsychotics in Routine Clinical Practice: Findings From the CLAMORS Study. *The Journal of Clinical Psychiatry*, *71*(3), 280–286. https://doi.org/10.4088/JCP.08m04250yel](https://www.zotero.org/google-docs/?SJrRhI)

[Bond, G. R., & Drake, R. E. (2008). Predictors of competitive employment among patients with schizophrenia. *Current Opinion in Psychiatry*, *21*(4), 362–369. https://doi.org/10.1097/YCO.0b013e328300eb0e](https://www.zotero.org/google-docs/?SJrRhI)

[Bond, G. R., Resnick, S. G., Drake, R. E., Xie, H., McHugo, G. J., & Bebout, R. R. (2001). Does competitive employment improve nonvocational outcomes for people with severe mental illness? *Journal of Consulting and Clinical Psychology*, *69*(3), 489–501. https://doi.org/10.1037/0022-006X.69.3.489](https://www.zotero.org/google-docs/?SJrRhI)

[Bora, E., Akdede, B. B., & Alptekin, K. (2017). The relationship between cognitive impairment in schizophrenia and metabolic syndrome: A systematic review and meta-analysis. *Psychol Med*, *47*(6), 1030–1040. https://doi.org/10.1017/S0033291716003366](https://www.zotero.org/google-docs/?SJrRhI)

[Bora, Emre, Eryavuz, A., Kayahan, B., Sungu, G., & Veznedaroglu, B. (2006). Social functioning, theory of mind and neurocognition in outpatients with schizophrenia; mental state decoding may be a better predictor of social functioning than mental state reasoning. *Psychiatry Research*, *145*(2–3), 95–103. https://doi.org/10.1016/j.psychres.2005.11.003](https://www.zotero.org/google-docs/?SJrRhI)

[Bourne, P., Meddings, S., & Whittington, A. (2018). An evaluation of service use outcomes in a Recovery College. *Journal of Mental Health*, *27*(4), 359–366. https://doi.org/10.1080/09638237.2017.1417557](https://www.zotero.org/google-docs/?SJrRhI)

[Bowie, C. R., Bell, M. D., Fiszdon, J. M., Johannesen, J. K., Lindenmayer, J.-P., McGurk, S. R., Medalia, A. A., Penadés, R., Saperstein, A. M., Twamley, E. W., Ueland, T., & Wykes, T. (2020). Cognitive remediation for schizophrenia: An expert working group white paper on core techniques. *Schizophrenia Research*, *215*, 49–53. https://doi.org/10.1016/j.schres.2019.10.047](https://www.zotero.org/google-docs/?SJrRhI)

[Bowie, C. R., Depp, C., McGrath, J. A., Wolyniec, P., Mausbach, B. T., Thornquist, M. H., Luke, J., Patterson, T. L., Harvey, P. D., & Pulver, A. E. (2010). Prediction of real-world functional disability in chronic mental disorders: A comparison of schizophrenia and bipolar disorder. *The American Journal of Psychiatry*, *167*(9), 1116–1124. https://doi.org/10.1176/appi.ajp.2010.09101406](https://www.zotero.org/google-docs/?SJrRhI)

[Bowie, C. R., Leung, W. W., Reichenberg, A., McClure, M. M., Patterson, T. L., Heaton, R. K., & Harvey, P. D. (2008). Predicting schizophrenia patients’ real-world behavior with specific neuropsychological and functional capacity measures. *Biological Psychiatry*, *63*(5), 505–511. https://doi.org/10.1016/j.biopsych.2007.05.022](https://www.zotero.org/google-docs/?SJrRhI)

[Bowie, C. R., Reichenberg, A., Patterson, T. L., Heaton, R. K., & Harvey, P. D. (2006). Determinants of Real-World Functional Performance  in Schizophrenia Subjects: Correlations With Cognition, Functional Capacity, and Symptoms. *American Journal of Psychiatry*, *163*(3), 418–425. https://doi.org/10.1176/appi.ajp.163.3.418](https://www.zotero.org/google-docs/?SJrRhI)

[Bow-Thomas, C. C., Velligan, D. I., Miller, A. L., & Olsen, J. (1999). Predicting quality of life from symptomatology in schizophrenia at exacerbation and stabilization. *Psychiatry Research*, *86*(2), 131–142. https://doi.org/10.1016/S0165-1781(99)00023-2](https://www.zotero.org/google-docs/?SJrRhI)

[Boyko, E. J., Fujimoto, W. Y., Leonetti, D. L., & Newell-Morris, L. (2000). Visceral adiposity and risk of type 2 diabetes: A prospective study among Japanese Americans. *Diabetes Care*, *23*(4), 465–471. https://doi.org/10.2337/diacare.23.4.465](https://www.zotero.org/google-docs/?SJrRhI)

[Brekke, J., Kay, D. D., Lee, K. S., & Green, M. F. (2005). Biosocial pathways to functional outcome in schizophrenia. *Schizophrenia Research*, *80*(2–3), 213–225. https://doi.org/10.1016/j.schres.2005.07.008](https://www.zotero.org/google-docs/?SJrRhI)

[Brekke, J. S., DeBonis, J. A., & Graham, J. W. (1994). A latent structure analysis of the positive and negative symptoms in schizophrenia. *Comprehensive Psychiatry*, *35*(4), 252–259. https://doi.org/10.1016/0010-440X(94)90016-7](https://www.zotero.org/google-docs/?SJrRhI)

[Brekke, J. S., Hoe, M., Long, J., & Green, M. F. (2007). How neurocognition and social cognition influence functional change during community-based psychosocial rehabilitation for individuals with schizophrenia. *Schizophrenia Bulletin*, *33*(5), 1247–1256. https://doi.org/10.1093/schbul/sbl072](https://www.zotero.org/google-docs/?SJrRhI)

[Brown, S. D., Unger Hu, K. A., Mevi, A. A., Hedderson, M. M., Shan, J., Quesenberry, C. P., & Ferrara, A. (2014). The multigroup ethnic identity measure-revised: Measurement invariance across racial and ethnic groups. *Journal of Counseling Psychology*, *61*(1), 154–161. https://doi.org/10.1037/a0034749](https://www.zotero.org/google-docs/?SJrRhI)

[Brunelin, J., Mondino, M., Gassab, L., Haesebaert, F., Gaha, L., Suaud-Chagny, M.-F., Saoud, M., Mechri, A., & Poulet, E. (2012). Examining transcranial direct-current stimulation (tDCS) as a treatment for hallucinations in schizophrenia. *The American Journal of Psychiatry*, *169*(7), 719–724. https://doi.org/10.1176/appi.ajp.2012.11071091](https://www.zotero.org/google-docs/?SJrRhI)

[Bulik-Sullivan, B., Finucane, H. K., Anttila, V., Gusev, A., Day, F. R., Loh, P.-R., ReproGen Consortium, Psychiatric Genomics Consortium, Genetic Consortium for Anorexia Nervosa of the Wellcome Trust Case Control Consortium 3, Duncan, L., Perry, J. R. B., Patterson, N., Robinson, E. B., Daly, M. J., Price, A. L., & Neale, B. M. (2015). An atlas of genetic correlations across human diseases and traits. *Nature Genetics*, *47*(11), 1236–1241. https://doi.org/10.1038/ng.3406](https://www.zotero.org/google-docs/?SJrRhI)

[Burns, A. M., Erickson, D. H., & Brenner, C. A. (2014). Cognitive-behavioral therapy for medication-resistant psychosis: A meta-analytic review. *Psychiatr Serv*, *65*(7), 874–880. https://doi.org/10.1176/appi.ps.201300213](https://www.zotero.org/google-docs/?SJrRhI)

[Bush, K., Kivlahan, D. R., McDonell, M. B., Fihn, S. D., & Bradley, K. A. (1998). The AUDIT alcohol consumption questions (AUDIT-C): An effective brief screening test for problem drinking. Ambulatory Care Quality Improvement Project (ACQUIP). Alcohol Use Disorders Identification Test. *Arch Intern Med*, *158*(16), 1789–1795. https://doi.org/10.1001/archinte.158.16.1789](https://www.zotero.org/google-docs/?SJrRhI)

[Butler, G. S., Vallis, T. M., Perey, B., Veldhuyzen Van Zanten, S. J. O., MacDonald, A. S., & Konok, G. (1999). The Obesity Adjustment Survey: Development of a scale to assess psychological adjustment to morbid obesity. *International Journal of Obesity*, *23*(5). https://doi.org/10.1038/sj.ijo.0800850](https://www.zotero.org/google-docs/?SJrRhI)

[Caemmerer, J., Correll, C. U., & Maayan, L. (2012). Acute and maintenance effects of non-pharmacologic interventions for antipsychotic associated weight gain and metabolic abnormalities: A meta-analytic comparison of randomized controlled trials. *Schizophrenia Research*, *140*(1–3). https://doi.org/10.1016/j.schres.2012.03.017](https://www.zotero.org/google-docs/?SJrRhI)

[Calkin, C. V., Ruzickova, M., Uher, R., Hajek, T., Slaney, C. M., Garnham, J. S., O’Donovan, M. C., & Alda, M. (2015). Insulin resistance and outcome in bipolar disorder. *British Journal of Psychiatry*, *206*(1). https://doi.org/10.1192/bjp.bp.114.152850](https://www.zotero.org/google-docs/?SJrRhI)

[Canadian Community Health Survey. (2013). *Canadian Community Health Survey—Annual Component (CCHS)—2013 Questionnaire: Everyday Discrimination Scale / Component: Rapid Response—EDS*. Canadian Community Health Survey - Annual Component (CCHS) - 2013 Questionnaire: Everyday Discrimination Scale / Component: Rapid Response - EDS. https://www.statcan.gc.ca/eng/statistical-programs/instrument/3226_Q6_V1](https://www.zotero.org/google-docs/?SJrRhI)

[Cardinal, R. N. (2017). Clinical records anonymisation and text extraction (CRATE): An open-source software system. *BMC Medical Informatics and Decision Making*, *17*(1), 50. https://doi.org/10.1186/s12911-017-0437-1](https://www.zotero.org/google-docs/?SJrRhI)

[Caron, J., Mercier, C., Diaz, P., & Martin, A. (2005). Socio-demographic and clinical predictors of quality of life in patients with schizophrenia or schizo-affective disorder. *Psychiatry Research*, *137*(3), 203–213. https://doi.org/10.1016/j.psychres.2005.07.002](https://www.zotero.org/google-docs/?SJrRhI)

[Carpenter, W. T., & Kirkpatrick, B. (1988). The heterogeneity of the long-term course of schizophrenia. *Schizophrenia Bulletin*, *14*(4), 645–652.](https://www.zotero.org/google-docs/?SJrRhI)

[Carton, L., Cottencin, O., Lapeyre-Mestre, M., Geoffroy, P. A., Favre, J., Simon, N., Bordet, R., & Rolland, B. (2015). Off-Label Prescribing of Antipsychotics in Adults, Children and Elderly Individuals: A Systematic Review of Recent Prescription Trends. *Curr Pharm Des*, *21*(23), 3280–3297. https://doi.org/10.2174/1381612821666150619092903](https://www.zotero.org/google-docs/?SJrRhI)

[Cerimele, J. M., & Katon, W. J. (2013). Associations between health risk behaviors and symptoms of schizophrenia and bipolar disorder: A systematic review. *General Hospital Psychiatry*, *35*(1). https://doi.org/10.1016/j.genhosppsych.2012.08.001](https://www.zotero.org/google-docs/?SJrRhI)

[Chakos, M., Lieberman, J., Hoffman, E., Bradford, D., & Sheitman, B. (2001). Effectiveness of second-generation antipsychotics in patients with treatment-resistant schizophrenia: A review and meta-analysis of randomized trials. *The American Journal of Psychiatry*, *158*(4), 518–526.](https://www.zotero.org/google-docs/?SJrRhI)

[Chang, W. C., Chu, A. O. K., Kwong, V. W. Y., Wong, C. S. M., Hui, C. L.-M., Chan, S. K. W., Lee, E. H. M., & Chen, E. Y.-H. (2018). Patterns and predictors of trajectories for social and occupational functioning in patients presenting with first-episode non-affective psychosis: A three-year follow-up study. *Schizophrenia Research*, *197*(C), 131–137. https://doi.org/10.1016/j.schres.2018.01.021](https://www.zotero.org/google-docs/?SJrRhI)

[Chong, H. Y., Teoh, S. L., Wu, D. B., Kotirum, S., Chiou, C. F., & Chaiyakunapruk, N. (2016). Global economic burden of schizophrenia: A systematic review. *Neuropsychiatr Dis Treat*, *12*, 357–373. https://doi.org/10.2147/NDT.S96649](https://www.zotero.org/google-docs/?SJrRhI)

[Chorlton, E., McKenzie, K., Morgan, C., & Doody, G. (2012). Course and outcome of psychosis in black Caribbean populations and other ethnic groups living in the UK: A systematic review. *International Journal of Social Psychiatry*, *58*(4), 400–408. https://doi.org/10.1177/0020764011403070](https://www.zotero.org/google-docs/?SJrRhI)

[Chumakov, I., Blumenfeld, M., Guerassimenko, O., Cavarec, L., Palicio, M., Abderrahim, H., Bougueleret, L., Barry, C., Tanaka, H., La Rosa, P., Puech, A., Tahri, N., Cohen-Akenine, A., Delabrosse, S., Lissarrague, S., Picard, F. P., Maurice, K., Essioux, L., Millasseau, P., … Cohen, D. (2002). Genetic and physiological data implicating the new human gene G72 and the gene for D-amino acid oxidase in schizophrenia. *Proc Natl Acad Sci U S A*, *99*(21), 13675–13680. https://doi.org/10.1073/pnas.182412499](https://www.zotero.org/google-docs/?SJrRhI)

[Citrome, L. (2013). New second-generation long-acting injectable antipsychotics for the treatment of schizophrenia. *Expert Review of Neurotherapeutics*, *13*(7), 767–783. https://doi.org/10.1586/14737175.2013.811984](https://www.zotero.org/google-docs/?SJrRhI)

[Cloutier, M., Aigbogun, M. S., Guerin, A., Nitulescu, R., Ramanakumar, A. V., Kamat, S. A., DeLucia, M., Duffy, R., Legacy, S. N., Henderson, C., Francois, C., & Wu, E. (2016). The Economic Burden of Schizophrenia in the United States in 2013. *J Clin Psychiatry*, *77*(6), 764–771. https://doi.org/10.4088/JCP.15m10278](https://www.zotero.org/google-docs/?SJrRhI)

[Cloutier, M., Aigbogun, M. S., Guerin, A., Nitulescu, R., Ramanakumar, A. V., Kamat, S. A., DeLucia, M., Duffy, R., Legacy, S. N., Henderson, C., Francois, C., & Wu, E. (2016). The Economic Burden of Schizophrenia in the United States in 2013. *J Clin Psychiatry*, *77*(6), 764–771. https://doi.org/10.4088/JCP.15m10278](https://www.zotero.org/google-docs/?SJrRhI)

[Connell, M., King, R., & Crowe, T. (2011). Can employment positively affect the recovery of people with psychiatric disabilities? *Psychiatric Rehabilitation Journal*, *35*(1), 59–63. https://doi.org/10.2975/35.1.2011.59.63](https://www.zotero.org/google-docs/?SJrRhI)

[Connor, K. M., & Davidson, J. R. T. (2003). Development of a new resilience scale: The Connor-Davidson Resilience Scale (CD-RISC). *Depression and Anxiety*, *18*(2), 76–82. https://doi.org/10.1002/da.10113](https://www.zotero.org/google-docs/?SJrRhI)

Conrad, K. J., Yagelka, J. R., Matters, M. D., Rich, A. R., Williams, V., & Buchanan, M. (2001). Reliability and validity of a modified Colorado Symptom Index in a national homeless sample*. Mental Health Services Research, 3*(3), 141-153

[Cook, E. A., Liu, N. H., Tarasenko, M., Davidson, C. A., & Spaulding, W. D. (2013). Longitudinal relationships between neurocognition, theory of mind, and community functioning in outpatients with serious mental illness. *The Journal of Nervous and Mental Disease*, *201*(9), 786–794. https://doi.org/10.1097/NMD.0b013e3182a2140b](https://www.zotero.org/google-docs/?SJrRhI)

[Cook, J. A., Burke-Miller, J. K., & Roessel, E. (2016). Long-Term Effects of Evidence-Based Supported Employment on Earnings and on SSI and SSDI Participation Among Individuals With Psychiatric Disabilities. *American Journal of Psychiatry*, *173*(10), 1007–1014. https://doi.org/10.1176/appi.ajp.2016.15101359](https://www.zotero.org/google-docs/?SJrRhI)

[Cooper, S. J., Reynolds, G. P., Barnes, T. R. E., England, E., Haddad, P. M., Heald, A., Holt, R. I. G., Lingford-Hughes, A., Osborn, D., & McGowan, O. (2016). BAP guidelines on the management of weight gain, metabolic disturbances and cardiovascular risk associated with psychosis and antipsychotic drug treatment. *Journal of Psychopharmacology*, *30*(8), 717–748.](https://www.zotero.org/google-docs/?SJrRhI)

[Correll, C. U., Robinson, D. G., Schooler, N. R., Brunette, M. F., Mueser, K. T., Rosenheck, R. A., Marcy, P., Addington, J., Estroff, S. E., Robinson, J., Penn, D. L., Azrin, S., Goldstein, A., Severe, J., Heinssen, R., & Kane, J. M. (2014). Cardiometabolic risk in patients with first-episode schizophrenia spectrum disorders: Baseline results from the RAISE-ETP study. *JAMA Psychiatry*, *71*(12), 1350–1363. https://doi.org/10.1001/jamapsychiatry.2014.1314](https://www.zotero.org/google-docs/?SJrRhI)

[Correll, C. U., Solmi, M., Veronese, N., Bortolato, B., Rosson, S., Santonastaso, P., Thapa-Chhetri, N., Fornaro, M., Gallicchio, D., Collantoni, E., Pigato, G., Favaro, A., Monaco, F., Kohler, C., Vancampfort, D., Ward, P. B., Gaughran, F., Carvalho, A. F., & Stubbs, B. (2017). Prevalence, incidence and mortality from cardiovascular disease in patients with pooled and specific severe mental illness: A large-scale meta-analysis of 3,211,768 patients and 113,383,368 controls. *World Psychiatry*, *16*(2), 163–180. https://doi.org/10.1002/wps.20420](https://www.zotero.org/google-docs/?SJrRhI)

[Corrigan, P. W., Salzer, M., Ralph, R. O., Sangster, Y., & Keck, L. (2004). Examining the factor structure of the recovery assessment scale. *Schizophrenia Bulletin*, *30*(4), 1035–1041. https://doi.org/10.1093/oxfordjournals.schbul.a007118](https://www.zotero.org/google-docs/?SJrRhI)

[Couture, S. M., Granholm, E. L., & Fish, S. C. (2011). A path model investigation of neurocognition, theory of mind, social competence, negative symptoms and real-world functioning in schizophrenia. *Schizophrenia Research*, *125*(2–3), 152–160. https://doi.org/10.1016/j.schres.2010.09.020](https://www.zotero.org/google-docs/?SJrRhI)

Cuttler, C., & Spradlin, A. (2017). Measuring cannabis consumption: Psychometric properties of the Daily Sessions, Frequency, Age of Onset, and Quantity of Cannabis Use Inventory (DFAQ-CU). *PLOS ONE 12*(5): e0178194. <https://doi.org/10.1371/journal.pone.0178194>

[D’Arcey, J., Collaton, J., Kozloff, N., Voineskos, A. N., Kidd, S. A., & Foussias, G. (2020). The Use of Text Messaging to Improve Clinical Engagement for Individuals With Psychosis: Systematic Review. *JMIR Ment Health*, *7*(4), e16993. https://doi.org/10.2196/16993](https://www.zotero.org/google-docs/?SJrRhI)

[Dauwan, M., Begemann, M. J. H., Heringa, S. M., & Sommer, I. E. (2016). Exercise improves clinical symptoms, quality of life, global functioning, and depression in schizophrenia: A systematic review and meta-analysis. *Schizophrenia Bulletin*, *42*(3). https://doi.org/10.1093/schbul/sbv164](https://www.zotero.org/google-docs/?SJrRhI)

[Davidson, L., & Stern, E. (2013). Psychiatric/Psychosocial Rehabilitation (PSR) in Relation to Social and Leisure Environments: Friends and Recreation. *Current Psychiatry Reviews*, *9*(3), 207–213.](https://www.zotero.org/google-docs/?SJrRhI)

[Davison, J., O’Gorman, A., Brennan, L., & Cotter, D. R. (2018). A systematic review of metabolite biomarkers of schizophrenia. *Schizophrenia Research*, *195*(Complete), 32–50. https://doi.org/10.1016/j.schres.2017.09.021](https://www.zotero.org/google-docs/?SJrRhI)

[De Hert, M. A., Van Winkel, R., Van Eyck, D., Hanssens, L., Wampers, M., Scheen, A., & Peuskens, J. (2006a). Prevalence of diabetes, metabolic syndrome and metabolic abnormalities in schizophrenia over the course of the illness: A cross-sectional study. *Clin Pract Epidemiol Ment Health*, *2*, 14. https://doi.org/1745-0179-2-14 [pii] 10.1186/1745-0179-2-14](https://www.zotero.org/google-docs/?SJrRhI)

[De Hert, M. A., Van Winkel, R., Van Eyck, D., Hanssens, L., Wampers, M., Scheen, A., & Peuskens, J. (2006b). Prevalence of the metabolic syndrome in patients with schizophrenia treated with antipsychotic medication. *Schizophrenia Research*, *83*(1). https://doi.org/10.1016/j.schres.2005.12.855](https://www.zotero.org/google-docs/?SJrRhI)

[De Hert, M., Detraux, J., van Winkel, R., Yu, W., & Correll, C. U. (2011). Metabolic and cardiovascular adverse effects associated with antipsychotic drugs. *Nat Rev Endocrinol*, *8*(2), 114–126. https://doi.org/10.1038/nrendo.2011.156](https://www.zotero.org/google-docs/?SJrRhI)

[De Hert, Marc, Sermon, J., Geerts, P., Vansteelandt, K., Peuskens, J., & Detraux, J. (2015). The Use of Continuous Treatment Versus Placebo or Intermittent Treatment Strategies in Stabilized Patients with Schizophrenia: A Systematic Review and Meta-Analysis of Randomized Controlled Trials with First- and Second-Generation Antipsychotics. *CNS Drugs*, *29*(8), 637–658. https://doi.org/10.1007/s40263-015-0269-4](https://www.zotero.org/google-docs/?SJrRhI)

[De Mare, A., Cantarella, M., & Galeoto, G. (2018). Effectiveness of Integrated Neurocognitive Therapy on Cognitive Impairment and Functional Outcome for Schizophrenia Outpatients. *Schizophrenia Research and Treatment*, *2018*, 2360697. https://doi.org/10.1155/2018/2360697](https://www.zotero.org/google-docs/?SJrRhI)

DeSilva, M.J., Harpham, T., Tuan, T., Bartolini, R., Penny, M.E., & Huttly, S.R. (2006). Psychometric and cognitive validation of a social capital measurement tool in Peru and Vietnam. *Social Science & Medicine 62,* 941-953. https://doi.org/ doi:10.1016/j.socscimed.2005.06.050

[de Silva, V. A., Suraweera, C., Ratnatunga, S. S., Dayabandara, M., Wanniarachchi, N., & Hanwella, R. (2016). Metformin in prevention and treatment of antipsychotic induced weight gain: A systematic review and meta-analysis. *BMC Psychiatry*, *16*(1), 341. https://doi.org/10.1186/s12888-016-1049-5](https://www.zotero.org/google-docs/?SJrRhI)

[Dekkers, I. A., Jansen, P. R., & Lamb, H. J. (2019). Obesity, Brain Volume, and White Matter Microstructure at MRI: A Cross-sectional UK Biobank Study. *Radiology*, *292*(1), 270. https://doi.org/10.1148/radiol.2019194010](https://www.zotero.org/google-docs/?SJrRhI)

[DeTore, N. R., Mueser, K. T., & McGurk, S. R. (2018). What does the Managing Emotions branch of the MSCEIT add to the MATRICS consensus cognitive battery? *Schizophrenia Research*, *197*, 414–420. https://doi.org/10.1016/j.schres.2018.02.018](https://www.zotero.org/google-docs/?SJrRhI)

[Dickerson, F., Boronow, J. J., Ringel, N., & Parente, F. (1999). Social functioning and neurocognitive deficits in outpatients with schizophrenia: A 2-year follow-up1This work was presented in part at the Winter Workshop on Schizophrenia, Davos, Switzerland, 7–13 February 1998.1. *Schizophrenia Research*, *37*(1), 13–20. https://doi.org/10.1016/S0920-9964(98)00134-0](https://www.zotero.org/google-docs/?SJrRhI)

[Dickie, E. W., Anticevic, A., Smith, D. E., Coalson, T. S., Manogaran, M., Calarco, N., Viviano, J. D., Glasser, M. F., Van Essen, D. C., & Voineskos, A. N. (2019). Ciftify: A framework for surface-based analysis of legacy MR acquisitions. *NeuroImage*, *197*, 818–826. https://doi.org/10.1016/j.neuroimage.2019.04.078](https://www.zotero.org/google-docs/?SJrRhI)

[Dixon, L., Weiden, P., Delahanty, J., Goldberg, R., Postrado, L., Lucksted, A., & Lehman, A. (2000). Prevalence and correlates of diabetes in national schizophrenia samples. *Schizophr Bull*, *26*(4), 903–912.](https://www.zotero.org/google-docs/?SJrRhI)

[Dixon, Lisa, Goldberg, R., Lehman, A., & McNary, S. (2001). The impact of health status on work, symptoms, and functional outcomes in severe mental illness. *Journal of Nervous and Mental Disease*, *189*(1). https://doi.org/10.1097/00005053-200101000-00004](https://www.zotero.org/google-docs/?SJrRhI)

[Donetto, S., Tsianakas, V., & Robert, G. (2014). Using Experience-based Co-design (EBCD) to improve the quality of healthcare: Mapping where we are now and establishing future directions. *Undefined*. /paper/Using-Experience-based-Co-design-(EBCD)-to-improve-Donetto-Tsianakas/259c2ebc8e3e39c42c4a1ac50df8ccd2f1e04994](https://www.zotero.org/google-docs/?SJrRhI)

[Donetto, Sara, Pierri, P., Tsianakas, V., & Robert, G. (2015). Experience-based Co-design and Healthcare Improvement: Realizing Participatory Design in the Public Sector. *The Design Journal*, *18*(2), 227–248. https://doi.org/10.2752/175630615X14212498964312](https://www.zotero.org/google-docs/?SJrRhI)

[Dong, M., Lu, L., Zhang, L., Zhang, Y. S., Ng, C. H., Ungvari, G. S., Li, G., Meng, X., Wang, G., & Xiang, Y. T. (2019). Quality of Life in Schizophrenia: A Meta-Analysis of Comparative Studies. *Psychiatr Q*, *90*(3), 519–532. https://doi.org/10.1007/s11126-019-09633-4](https://www.zotero.org/google-docs/?SJrRhI)

[Doyle, A., Lanoil, J., & Dudek, K. (2013). *Fountain House: Creating Community in Mental Health Practice*. Columbia University Press.](https://www.zotero.org/google-docs/?SJrRhI)

[Drake, R. E., & Whitley, R. (2014). Recovery and Severe Mental Illness: Description and Analysis. *The Canadian Journal of Psychiatry*, *59*(5), 236–242. https://doi.org/10.1177/070674371405900502](https://www.zotero.org/google-docs/?SJrRhI)

[Dunn, J. R., Hayes, M. V., Hulchanski, J. D., Hwang, S. W., & Potvin, L. (2006). Housing as a Socio-Economic Determinant of Health: Findings of a National Needs, Gaps and Opportunities Assessment. *Canadian Journal of Public Health / Revue Canadienne de Sante’e Publique*, *97*, S11–S15. JSTOR.](https://www.zotero.org/google-docs/?SJrRhI)

[Eack, S. M., & Newhill, C. E. (2007). Psychiatric symptoms and quality of life in schizophrenia: A meta-analysis. *Schizophr Bull*, *33*(5), 1225–1237. https://doi.org/10.1093/schbul/sbl071](https://www.zotero.org/google-docs/?SJrRhI)

[Eack, Shaun M., & Keshavan, M. S. (2020). Cognition, negative symptoms, and functional outcome in psychosis. *Schizophrenia Research*, *224*, 22–23. https://doi.org/10.1016/j.schres.2020.06.029](https://www.zotero.org/google-docs/?SJrRhI)

[Elliott, M. L., Knodt, A. R., Cooke, M., Kim, M. J., Melzer, T. R., Keenan, R., Ireland, D., Ramrakha, S., Poulton, R., Caspi, A., Moffitt, T. E., & Hariri, A. R. (2019). General functional connectivity: Shared features of resting-state and task fMRI drive reliable and heritable individual differences in functional brain networks. *NeuroImage*, *189*, 516–532. https://doi.org/10.1016/j.neuroimage.2019.01.068](https://www.zotero.org/google-docs/?SJrRhI)

[Emerging Risk Factors Collaboration, Wormser, D., Kaptoge, S., Di Angelantonio, E., Wood, A. M., Pennells, L., Thompson, A., Sarwar, N., Kizer, J. R., Lawlor, D. A., Nordestgaard, B. G., Ridker, P., Salomaa, V., Stevens, J., Woodward, M., Sattar, N., Collins, R., Thompson, S. G., Whitlock, G., & Danesh, J. (2011). Separate and combined associations of body-mass index and abdominal adiposity with cardiovascular disease: Collaborative analysis of 58 prospective studies. *Lancet (London, England)*, *377*(9771), 1085–1095. https://doi.org/10.1016/S0140-6736(11)60105-0](https://www.zotero.org/google-docs/?SJrRhI)

[Emsley, R., Oosthuizen, P., Koen, L., Niehaus, D. J. H., Medori, R., & Rabinowitz, J. (2008). Oral versus injectable antipsychotic treatment in early psychosis: Post hoc comparison of two studies. *Clinical Therapeutics*, *30*(12), 2378–2386. https://doi.org/10.1016/j.clinthera.2008.12.020](https://www.zotero.org/google-docs/?SJrRhI)

[Esch, P., Bocquet, V., Pull, C., Couffignal, S., Lehnert, T., Graas, M., Fond-Harmant, L., & Ansseau, M. (2014). The downward spiral of mental disorders and educational attainment: A systematic review on early school leaving. *BMC Psychiatry*, *14*(1), 237. https://doi.org/10.1186/s12888-014-0237-4](https://www.zotero.org/google-docs/?SJrRhI)

[Esteban, O., Markiewicz, C. J., Blair, R. W., Moodie, C. A., Isik, A. I., Erramuzpe, A., Kent, J. D., Goncalves, M., DuPre, E., Snyder, M., Oya, H., Ghosh, S. S., Wright, J., Durnez, J., Poldrack, R. A., & Gorgolewski, K. J. (2019). fMRIPrep: A robust preprocessing pipeline for functional MRI. *Nature Methods*, *16*(1), 111–116. https://doi.org/10.1038/s41592-018-0235-4](https://www.zotero.org/google-docs/?SJrRhI)

[Euesden, J., Lewis, C. M., & O’Reilly, P. F. (2015). PRSice: Polygenic Risk Score software. *Bioinformatics (Oxford, England)*, *31*(9), 1466–1468. https://doi.org/10.1093/bioinformatics/btu848](https://www.zotero.org/google-docs/?SJrRhI)

[Evensen, S., Wisløff, T., Lystad, J. U., Bull, H., Ueland, T., & Falkum, E. (2016). Prevalence, Employment Rate, and Cost of Schizophrenia in a High-Income Welfare Society: A Population-Based Study Using Comprehensive Health and Welfare Registers. *Schizophrenia Bulletin*, *42*(2), 476–483. https://doi.org/10.1093/schbul/sbv141](https://www.zotero.org/google-docs/?SJrRhI)

[Faulkner, G., Cohn, T., Remington, G., & Irving, H. (2007). Body mass index, waist circumference and quality of life in individuals with schizophrenia. *Schizophrenia Research*, *90*(1), 174–178.](https://www.zotero.org/google-docs/?SJrRhI)

[Fenton, W. S., & McGlashan, T. H. (1994). Antecedents, symptom progression, and long-term outcome of the deficit syndrome in schizophrenia. *The American Journal of Psychiatry*, *151*(3), 351–356. https://doi.org/10.1176/ajp.151.3.351](https://www.zotero.org/google-docs/?SJrRhI)

[Fenton, Wayne S., & McGlashan, T. H. (1991). Natural History of Schizophrenia Subtypes: II. Positive and Negative Symptoms and Long-term Course. *Archives of General Psychiatry*, *48*(11), 978–986. https://doi.org/10.1001/archpsyc.1991.01810350018003](https://www.zotero.org/google-docs/?SJrRhI)

[Fervaha, G., Agid, O., Takeuchi, H., Foussias, G., & Remington, G. (2013). Life satisfaction among individuals with schizophrenia in the Clinical Antipsychotic Trial of Intervention Effectiveness (CATIE) study. *The American Journal of Psychiatry*, *170*(9), 1061–1062. https://doi.org/10.1176/appi.ajp.2013.13010060](https://www.zotero.org/google-docs/?SJrRhI)

[Fervaha, G., Agid, O., Takeuchi, H., Foussias, G., & Remington, G. (2016). Life satisfaction and happiness among young adults with schizophrenia. *Psychiatry Research*, *242*, 174–179. https://doi.org/10.1016/j.psychres.2016.05.046](https://www.zotero.org/google-docs/?SJrRhI)

[Fervaha, G., Foussias, G., Agid, O., & Remington, G. (2014). Impact of primary negative symptoms on functional outcomes in schizophrenia. *European Psychiatry*, *29*(7), 449–455. https://doi.org/10.1016/j.eurpsy.2014.01.007](https://www.zotero.org/google-docs/?SJrRhI)

[Fett, A.-K. J., Lemmers-Jansen, I. L. J., & Krabbendam, L. (2019). Psychosis and urbanicity: A review of the recent literature from epidemiology to neurourbanism. *Current Opinion in Psychiatry*, *32*(3), 232–241. https://doi.org/10.1097/YCO.0000000000000486](https://www.zotero.org/google-docs/?SJrRhI)

[Fett, A.-K. J., Viechtbauer, W., Dominguez, M.-G., Penn, D. L., van Os, J., & Krabbendam, L. (2011). The relationship between neurocognition and social cognition with functional outcomes in schizophrenia: A meta-analysis. *Neuroscience and Biobehavioral Reviews*, *35*(3), 573–588. https://doi.org/10.1016/j.neubiorev.2010.07.001](https://www.zotero.org/google-docs/?SJrRhI)

[First, M. B., Williams, J. B. W., Karg, R. S., & Spitzer, R. L. (2015). *Structured Clinical Interview for DSM-5—Research Version (SCID-5 for DSM-5, Research Version; SCID-5-RV)*. American Psychiatric Association.](https://www.zotero.org/google-docs/?SJrRhI)

[Firth, J., Carney, R., Pownall, M., French, P., Elliott, R., Cotter, J., & Yung, A. R. (2017). Challenges in implementing an exercise intervention within residential psychiatric care: A mixed methods study. *Ment Health Phys Act*, *12*, 141–146. https://doi.org/10.1016/j.mhpa.2017.04.004](https://www.zotero.org/google-docs/?SJrRhI)

[Firth, J., Cotter, J., Elliott, R., French, P., & Yung, A. R. (2015). *A systematic review and meta-Analysis of exercise interventions in schizophrenia patients*.](https://www.zotero.org/google-docs/?SJrRhI)

[Firth, J., Stubbs, B., Rosenbaum, S., Vancampfort, D., Malchow, B., Schuch, F., Elliott, R., Nuechterlein, K. H., & Yung, A. R. (2017). Aerobic Exercise Improves Cognitive Functioning in People With Schizophrenia: A Systematic Review and Meta-Analysis. *Schizophr Bull*, *43*(3), 546–556. https://doi.org/10.1093/schbul/sbw115](https://www.zotero.org/google-docs/?SJrRhI)

[Fischl, B. (2012). FreeSurfer. *NeuroImage*, *62*(2), 774–781. https://doi.org/10.1016/j.neuroimage.2012.01.021](https://www.zotero.org/google-docs/?SJrRhI)

[Fitzgerald, P. B., McQueen, S., Daskalakis, Z. J., & Hoy, K. E. (2014). A Negative Pilot Study of Daily Bimodal Transcranial Direct Current Stimulation in Schizophrenia. *Brain Stimulation*, *7*(6), 813–816. https://doi.org/10.1016/j.brs.2014.08.002](https://www.zotero.org/google-docs/?SJrRhI)

[Fohner, A. E., Greene, J. D., Lawson, B. L., Chen, J. H., Kipnis, P., Escobar, G. J., & Liu, V. X. (2019). Assessing clinical heterogeneity in sepsis through treatment patterns and machine learning. *J Am Med Inform Assoc*, *26*(12), 1466–1477. https://doi.org/10.1093/jamia/ocz106](https://www.zotero.org/google-docs/?SJrRhI)

[Foldemo, A., Wärdig, R., Bachrach-Lindström, M., Edman, G., Holmberg, T., Lindström, T., Valter, L., & Ösby, U. (2014). Health-related quality of life and metabolic risk in patients with psychosis. *Schizophrenia Research*, *152*(1). https://doi.org/10.1016/j.schres.2013.11.029](https://www.zotero.org/google-docs/?SJrRhI)

[Ford, E., Carroll, J. A., Smith, H. E., Scott, D., & Cassell, J. A. (2016). Extracting information from the text of electronic medical records to improve case detection: A systematic review. *J Am Med Inform Assoc*, *23*(5), 1007–1015. https://doi.org/10.1093/jamia/ocv180](https://www.zotero.org/google-docs/?SJrRhI)

[Forti, M. D., Quattrone, D., Freeman, T. P., Tripoli, G., Gayer-Anderson, C., Quigley, H., Rodriguez, V., Jongsma, H. E., Ferraro, L., Cascia, C. L., Barbera, D. L., Tarricone, I., Berardi, D., Szöke, A., Arango, C., Tortelli, A., Velthorst, E., Bernardo, M., Del-Ben, C. M., … Ven, E. van der. (2019). The contribution of cannabis use to variation in the incidence of psychotic disorder across Europe (EU-GEI): A multicentre case-control study. *The Lancet Psychiatry*, *6*(5), 427–436. https://doi.org/10.1016/S2215-0366(19)30048-3](https://www.zotero.org/google-docs/?SJrRhI)

[Frank, J., Lang, M., Witt, S. H., Strohmaier, J., Rujescu, D., Cichon, S., Degenhardt, F., Nothen, M. M., Collier, D. A., Ripke, S., Naber, D., & Rietschel, M. (2015). Identification of increased genetic risk scores for schizophrenia in treatment-resistant patients. *Mol Psychiatry*, *20*(2), 150–151. https://doi.org/10.1038/mp.2014.56](https://www.zotero.org/google-docs/?SJrRhI)

[Frederick, D. E., & VanderWeele, T. J. (2019). Supported employment: Meta-analysis and review of randomized controlled trials of individual placement and support. *PLoS ONE*, *14*(2). https://doi.org/10.1371/journal.pone.0212208](https://www.zotero.org/google-docs/?SJrRhI)

[Fröhlich, F., Burrello, T. N., Mellin, J. M., Cordle, A. L., Lustenberger, C. M., Gilmore, J. H., & Jarskog, L. F. (2016). Exploratory study of once-daily transcranial direct current stimulation (tDCS) as a treatment for auditory hallucinations in schizophrenia. *European Psychiatry*, *33*, 54–60. https://doi.org/10.1016/j.eurpsy.2015.11.005](https://www.zotero.org/google-docs/?SJrRhI)

[Fujino, H., Sumiyoshi, C., Sumiyoshi, T., Yasuda, Y., Yamamori, H., Ohi, K., Fujimoto, M., Hashimoto, R., Takeda, M., & Imura, O. (2016). Predicting employment status and subjective quality of life in patients with schizophrenia. *Schizophrenia Research. Cognition*, *3*, 20–25. https://doi.org/10.1016/j.scog.2015.10.005](https://www.zotero.org/google-docs/?SJrRhI)

[Fulford, D., Niendam, T. A., Floyd, E. G., Carter, C. S., Mathalon, D. H., Vinogradov, S., Stuart, B. K., & Loewy, R. L. (2013). Symptom dimensions and functional impairment in early psychosis: More to the story than just negative symptoms. *Schizophrenia Research*, *147*(1), 125–131. https://doi.org/10.1016/j.schres.2013.03.024](https://www.zotero.org/google-docs/?SJrRhI)

[Fusar-Poli, P., De Micheli, A., Patel, R., Signorini, L., Miah, S., Spencer, T., & McGuire, P. (2020). Real-World Clinical Outcomes Two Years After Transition to Psychosis in Individuals at Clinical High Risk: Electronic Health Record Cohort Study. *Schizophr Bull*. https://doi.org/10.1093/schbul/sbaa040](https://www.zotero.org/google-docs/?SJrRhI)

[Fusar-Poli, Paolo, Papanastasiou, E., Stahl, D., Rocchetti, M., Carpenter, W., Shergill, S., & McGuire, P. (2015). Treatments of Negative Symptoms in Schizophrenia: Meta-Analysis of 168 Randomized Placebo-Controlled Trials. *Schizophrenia Bulletin*, *41*(4), 892–899. https://doi.org/10.1093/schbul/sbu170](https://www.zotero.org/google-docs/?SJrRhI)

[Galderisi, S., Bucci, P., Mucci, A., Kirkpatrick, B., Pini, S., Rossi, A., Vita, A., & Maj, M. (2013). Categorical and dimensional approaches to negative symptoms of schizophrenia: Focus on long-term stability and functional outcome. *Schizophrenia Research*, *147*(1), 157–162. https://doi.org/10.1016/j.schres.2013.03.020](https://www.zotero.org/google-docs/?SJrRhI)

[Galderisi, S., Maj, M., Mucci, A., Cassano, G. B., Invernizzi, G., Rossi, A., Vita, A., Dell’Osso, L., Daneluzzo, E., & Pini, S. (2002). Historical, Psychopathological, Neurological, and Neuropsychological Aspects of Deficit Schizophrenia: A Multicenter Study. *American Journal of Psychiatry*, *159*(6), 983–990. https://doi.org/10.1176/appi.ajp.159.6.983](https://www.zotero.org/google-docs/?SJrRhI)

[Galderisi, S., Rucci, P., Kirkpatrick, B., Mucci, A., Gibertoni, D., Rocca, P., Rossi, A., Bertolino, A., Strauss, G. P., Aguglia, E., Bellomo, A., Murri, M. B., Bucci, P., Carpiniello, B., Comparelli, A., Cuomo, A., De Berardis, D., Dell’Osso, L., Di Fabio, F., … Italian Network for Research on Psychoses. (2018). Interplay Among Psychopathologic Variables, Personal Resources, Context-Related Factors, and Real-life Functioning in Individuals With Schizophrenia: A Network Analysis. *JAMA Psychiatry*, *75*(4), 396–404. https://doi.org/10.1001/jamapsychiatry.2017.4607](https://www.zotero.org/google-docs/?SJrRhI)

[Galling, B., Vernon, J. A., Pagsberg, A. K., Wadhwa, A., Grudnikoff, E., Seidman, A. J., Tsoy-Podosenin, M., Poyurovsky, M., Kane, J. M., & Correll, C. U. (2018). Efficacy and safety of antidepressant augmentation of continued antipsychotic treatment in patients with schizophrenia. *Acta Psychiatrica Scandinavica*, *137*(3), 187–205. https://doi.org/10.1111/acps.12854](https://www.zotero.org/google-docs/?SJrRhI)

[Galling, Britta, Roldán, A., Hagi, K., Rietschel, L., Walyzada, F., Zheng, W., Cao, X.-L., Xiang, Y.-T., Zink, M., Kane, J. M., Nielsen, J., Leucht, S., & Correll, C. U. (2017). Antipsychotic augmentation vs. monotherapy in schizophrenia: Systematic review, meta-analysis and meta-regression analysis. *World Psychiatry: Official Journal of the World Psychiatric Association (WPA)*, *16*(1), 77–89. https://doi.org/10.1002/wps.20387](https://www.zotero.org/google-docs/?SJrRhI)

[García-García, I., Michaud, A., Dadar, M., Zeighami, Y., Neseliler, S., Collins, D. L., Evans, A. C., & Dagher, A. (2019). Neuroanatomical differences in obesity: Meta-analytic findings and their validation in an independent dataset. *International Journal of Obesity (2005)*, *43*(5), 943–951. https://doi.org/10.1038/s41366-018-0164-4](https://www.zotero.org/google-docs/?SJrRhI)

[Gard, D. E., Fisher, M., Garrett, C., Genevsky, A., & Vinogradov, S. (2009). Motivation and its relationship to neurocognition, social cognition, and functional outcome in schizophrenia. *Schizophrenia Research*, *115*(1), 74–81. https://doi.org/10.1016/j.schres.2009.08.015](https://www.zotero.org/google-docs/?SJrRhI)

[Garnov, N., Linder, N., Schaudinn, A., Blüher, M., Karlas, T., Schütz, T., Dietrich, A., Kahn, T., & Busse, H. (2014). Comparison of T1 relaxation times in adipose tissue of severely obese patients and healthy lean subjects measured by 1.5 T MRI. *NMR in Biomedicine*, *27*(9), 1123–1128. https://doi.org/10.1002/nbm.3166](https://www.zotero.org/google-docs/?SJrRhI)

[Gasse, C., Wimberley, T., Wang, Y., Mors, O., Borglum, A., Als, T. D., Werge, T., Nordentoft, M., Hougaard, D. M., & Horsdal, H. T. (2019). Schizophrenia polygenic risk scores, urbanicity and treatment-resistant schizophrenia. *Schizophr Res*, *212*, 79–85. https://doi.org/10.1016/j.schres.2019.08.008](https://www.zotero.org/google-docs/?SJrRhI)

[Gault, V. A., Porter, W. D., Flatt, P. R., & Holscher, C. (2010). Actions of exendin-4 therapy on cognitive function and hippocampal synaptic plasticity in mice fed a high-fat diet. *Int J Obes (Lond)*, *34*(8), 1341–1344. https://doi.org/ijo201059 [pii] 10.1038/ijo.2010.59](https://www.zotero.org/google-docs/?SJrRhI)

[Geraci, J., Wilansky, P., de Luca, V., Roy, A., Kennedy, J. L., & Strauss, J. (2017). Applying deep neural networks to unstructured text notes in electronic medical records for phenotyping youth depression. *Evid Based Ment Health*, *20*(3), 83–87. https://doi.org/10.1136/eb-2017-102688](https://www.zotero.org/google-docs/?SJrRhI)

[Gerretsen, P., Remington, G., Borlido, C., Quilty, L., Hassan, S., Polsinelli, G., Teo, C., Mar, W., Simon, R., Menon, M., Pothier, D. D., Nakajima, S., Caravaggio, F., Mamo, D. C., Rajji, T. K., Mulsant, B. H., Deluca, V., Ganguli, R., Pollock, B. G., & Graff-Guerrero, A. (2014). The VAGUS insight into psychosis scale—Self-report and clinician-rated versions. *Psychiatry Res*, *220*(3), 1084–1089. https://doi.org/10.1016/j.psychres.2014.08.005](https://www.zotero.org/google-docs/?SJrRhI)

[Gerszten, R. E., & Wang, T. J. (2008). The search for new cardiovascular biomarkers. *Nature*, *451*(7181), 949–952. https://doi.org/10.1038/nature06802](https://www.zotero.org/google-docs/?SJrRhI)

[Gil, A., Gama, C. S., de Jesus, D. R., Lobato, M. I., Zimmer, M., & Belmonte-de-Abreu, P. (2009). The association of child abuse and neglect with adult disability in schizophrenia and the prominent role of physical neglect. *Child Abuse & Neglect*, *33*(9), 618–624. https://doi.org/10.1016/j.chiabu.2009.02.006](https://www.zotero.org/google-docs/?SJrRhI)

[Gluhoski, V. L., & Wortman, C. B. (1996). The Impact of Trauma on World Views. *Journal of Social and Clinical Psychology*, *15*(4), 417–429. https://doi.org/10.1521/jscp.1996.15.4.417](https://www.zotero.org/google-docs/?SJrRhI)

[Goeree, R., Farahati, F., Burke, N., Blackhouse, G., O&apos;Reilly, D., Pyne, J., & Tarride, J.-E. (2005). The economic burden of schizophrenia in Canada in 2004. *Current Medical Research and Opinion*, *21*(12), 2017–2028. https://doi.org/10.1185/030079905X75087](https://www.zotero.org/google-docs/?SJrRhI)

[Goering, P. N., Streiner, D. L., Adair, C., Aubry, T., Barker, J., Distasio, J., Hwang, S. W., Komaroff, J., Latimer, E., Somers, J., & Zabkiewicz, D. M. (2011). The At Home/Chez Soi trial protocol: A pragmatic, multi-site, randomised controlled trial of a Housing First intervention for homeless individuals with mental illness in five Canadian cities. *BMJ Open*, *1*(2), e000323–e000323. https://doi.org/10.1136/bmjopen-2011-000323](https://www.zotero.org/google-docs/?SJrRhI)

[Goghari, V. M., Harrow, M., Grossman, L. S., & Rosen, C. (2013). A 20-year multi-follow-up of hallucinations in schizophrenia, other psychotic, and mood disorders. *Psychological Medicine*, *43*(6), 1151–1160. https://doi.org/10.1017/S0033291712002206](https://www.zotero.org/google-docs/?SJrRhI)

[Gomes, J. S., Shiozawa, P., Dias, Á. M., Valverde Ducos, D., Akiba, H., Trevizol, A. P., Bikson, M., Aboseria, M., Gadelha, A., de Lacerda, A. L. T., & Cordeiro, Q. (2015). Left Dorsolateral Prefrontal Cortex Anodal tDCS Effects on Negative Symptoms in Schizophrenia. *Brain Stimulation*, *8*(5), 991. https://doi.org/10.1016/j.brs.2015.07.033](https://www.zotero.org/google-docs/?SJrRhI)

[Gothelf, D., Eliez, S., Thompson, T., Hinard, C., Penniman, L., Feinstein, C., Kwon, H., Jin, S., Jo, B., Antonarakis, S. E., Morris, M. A., & Reiss, A. L. (2005). COMT genotype predicts longitudinal cognitive decline and psychosis in 22q11.2 deletion syndrome. *Nat Neurosci*, *8*(11), 1500–1502. https://doi.org/10.1038/nn1572](https://www.zotero.org/google-docs/?SJrRhI)

[Goulding, S. M., Chien, V. H., & Compton, M. T. (2010). Prevalence and correlates of school drop-out prior to initial treatment of nonaffective psychosis: Further evidence suggesting a need for supported education. *Schizophrenia Research*, *116*(2), 228–233. https://doi.org/10.1016/j.schres.2009.09.006](https://www.zotero.org/google-docs/?SJrRhI)

[Government of Ontario. (2020). *Ontario Health Teams: Guidance for Health Care Providers and Organizations*. 33.](https://www.zotero.org/google-docs/?SJrRhI)

[Grant, C., Addington, J., Addington, D., & Konnert, C. (2001). Social functioning in first- and multiepisode schizophrenia. *Canadian Journal of Psychiatry*, *46*(8), 746–749.](https://www.zotero.org/google-docs/?SJrRhI)

[Gray, M. J., Litz, B. T., Hsu, J. L., & Lombardo, T. W. (2004). Psychometric Properties of the Life Events Checklist. *Assessment*, *11*(4), 330–341. https://doi.org/10.1177/1073191104269954](https://www.zotero.org/google-docs/?SJrRhI)

[Green, M. F. (1996). What are the functional consequences of neurocognitive deficits in schizophrenia? *The American Journal of Psychiatry*, *153*(3), 321–330.](https://www.zotero.org/google-docs/?SJrRhI)

[Green, M. F., Kern, R. S., Braff, D. L., & Mintz, J. (2000). Neurocognitive deficits and functional outcome in schizophrenia: Are we measuring the “right stuff”? *Schizophrenia Bulletin*, *26*(1), 119–136.](https://www.zotero.org/google-docs/?SJrRhI)

[Greenwood, K. E., Landau, S., & Wykes, T. (2005). Negative Symptoms and Specific Cognitive Impairments as Combined Targets for Improved Functional Outcome Within Cognitive Remediation Therapy. *Schizophrenia Bulletin*, *31*(4), 910–921. https://doi.org/10.1093/schbul/sbi035](https://www.zotero.org/google-docs/?SJrRhI)

[Grover, S., Chakrabarti, S., Hazari, N., & Avasthi, A. (2017). Effectiveness of electroconvulsive therapy in patients with treatment resistant schizophrenia: A retrospective study. *Psychiatry Research*, *249*, 349–353. https://doi.org/10.1016/j.psychres.2017.01.042](https://www.zotero.org/google-docs/?SJrRhI)

[Grover, S., Sahoo, S., Rabha, A., & Koirala, R. (2019). ECT in schizophrenia: A review of the evidence. *Acta Neuropsychiatrica*, *31*(3), 115–127. https://doi.org/10.1017/neu.2018.32](https://www.zotero.org/google-docs/?SJrRhI)

[Guerriere, D. N., Ungar, W. J., Corey, M., Croxford, R., Tranmer, J. E., Tullis, E., & Coyte, P. C. (2006). Evaluation of the ambulatory and home care record: Agreement between self-reports and administrative data. *International Journal of Technology Assessment in Health Care*, *22*(2), 203–210. https://doi.org/10.1017/S0266462306051026](https://www.zotero.org/google-docs/?SJrRhI)

[Gupta, S., Richard, L., & Forsythe, A. (2015). The humanistic and economic burden associated with increasing body mass index in the EU5. *Diabetes, Metabolic Syndrome and Obesity: Targets and Therapy*, *8*. https://doi.org/10.2147/DMSO.S83696](https://www.zotero.org/google-docs/?SJrRhI)

[Guy, W. (1976). Clinical Global Impression (CGI). In W. Guy (Ed.), *ECDEU Assessment Manual for Psychopharmacology*. U.S. Department of Health, Education, and Welfare.](https://www.zotero.org/google-docs/?SJrRhI)

[Habtewold, T. D., Liemburg, E. J., Islam, M. A., de Zwarte, S. M. C., Boezen, H. M., Group Investigators, Bruggeman, R., & Alizadeh, B. Z. (2020). Association of schizophrenia polygenic risk score with data-driven cognitive subtypes: A six-year longitudinal study in patients, siblings and controls. *Schizophr Res*, *223*, 135–147. https://doi.org/10.1016/j.schres.2020.05.020](https://www.zotero.org/google-docs/?SJrRhI)

[Hajek, T., Calkin, C., Blagdon, R., Slaney, C., Uher, R., & Alda, M. (2014). Insulin Resistance, Diabetes Mellitus, and Brain Structure in Bipolar Disorders. *Neuropsychopharmacology*. https://doi.org/npp2014148 [pii] 10.1038/npp.2014.148](https://www.zotero.org/google-docs/?SJrRhI)

[Hakulinen, C., McGrath, J. J., Timmerman, A., Skipper, N., Mortensen, P. B., Pedersen, C. B., & Agerbo, E. (2019). The association between early-onset schizophrenia with employment, income, education, and cohabitation status: Nationwide study with 35 years of follow-up. *Social Psychiatry and Psychiatric Epidemiology*, *54*(11), 1343–1351. https://doi.org/10.1007/s00127-019-01756-0](https://www.zotero.org/google-docs/?SJrRhI)

[Hall, M.-H., Holton, K. M., Öngür, D., Montrose, D., & Keshavan, M. S. (2019). Longitudinal trajectory of early functional recovery in patients with first episode psychosis. *Schizophrenia Research*, *209*, 234–244. https://doi.org/10.1016/j.schres.2019.02.003](https://www.zotero.org/google-docs/?SJrRhI)

[Halverson, T. F., Orleans-Pobee, M., Merritt, C., Sheeran, P., Fett, A.-K., & Penn, D. L. (2019). Pathways to functional outcomes in schizophrenia spectrum disorders: Meta-analysis of social cognitive and neurocognitive predictors. *Neuroscience & Biobehavioral Reviews*, *105*, 212–219. https://doi.org/10.1016/j.neubiorev.2019.07.020](https://www.zotero.org/google-docs/?SJrRhI)

[Hammond, C. (2004). Impacts of lifelong learning upon emotional resilience, psychological and mental health: Fieldwork evidence. *Oxford Review of Education - OXFORD REV EDUC*, *30*, 551–568. https://doi.org/10.1080/0305498042000303008](https://www.zotero.org/google-docs/?SJrRhI)

[Harding, C. M. (1988). Course types in schizophrenia: An analysis of European and American studies. *Schizophrenia Bulletin*, *14*(4), 633–643.](https://www.zotero.org/google-docs/?SJrRhI)

[Harris, A., Chen, W., Jones, S., Hulme, M., Burgess, P., & Sara, G. (2019). Community treatment orders increase community care and delay readmission while in force: Results from a large population-based study. *The Australian and New Zealand Journal of Psychiatry*, *53*(3), 228–235. https://doi.org/10.1177/0004867418758920](https://www.zotero.org/google-docs/?SJrRhI)

[Harrow, M., & Jobe, T. H. (2010). How frequent is chronic multiyear delusional activity and recovery in schizophrenia: A 20-year multi-follow-up. *Schizophrenia Bulletin*, *36*(1), 192–204. https://doi.org/10.1093/schbul/sbn074](https://www.zotero.org/google-docs/?SJrRhI)

[Harvey, P. D., Howanitz, E., Parrella, M., White, L., Davidson, M., Mohs, R. C., Hoblyn, J., & Davis, K. L. (1998). Symptoms, Cognitive Functioning, and Adaptive Skills in Geriatric Patients With Lifelong Schizophrenia: A Comparison Across Treatment Sites. *American Journal of Psychiatry*, *155*(8), 1080–1086. https://doi.org/10.1176/ajp.155.8.1080](https://www.zotero.org/google-docs/?SJrRhI)

[Hazell, C. M., Hayward, M., Cavanagh, K., & Strauss, C. (2016). A systematic review and meta-analysis of low intensity CBT for psychosis. *Clin Psychol Rev*, *45*, 183–192. https://doi.org/10.1016/j.cpr.2016.03.004](https://www.zotero.org/google-docs/?SJrRhI)

[Helfer, B., Samara, M. T., Huhn, M., Klupp, E., Leucht, C., Zhu, Y., Engel, R. R., & Leucht, S. (2016). Efficacy and Safety of Antidepressants Added to Antipsychotics for Schizophrenia: A Systematic Review and Meta-Analysis. *American Journal of Psychiatry*, *173*(9), 876–886. https://doi.org/10.1176/appi.ajp.2016.15081035](https://www.zotero.org/google-docs/?SJrRhI)

[Hennah, W., Tuulio-Henriksson, A., Paunio, T., Ekelund, J., Varilo, T., Partonen, T., Cannon, T. D., Lonnqvist, J., & Peltonen, L. (2005). A haplotype within the DISC1 gene is associated with visual memory functions in families with a high density of schizophrenia. *Mol Psychiatry*, *10*(12), 1097–1103. https://doi.org/10.1038/sj.mp.4001731](https://www.zotero.org/google-docs/?SJrRhI)

[Hennekens, C. H., Hennekens, A. R., Hollar, D., & Casey, D. E. (2005). Schizophrenia and increased risks of cardiovascular disease. *Am Heart J*, *150*(6), 1115–1121. https://doi.org/S0002-8703(05)00125-0 [pii] 10.1016/j.ahj.2005.02.007](https://www.zotero.org/google-docs/?SJrRhI)

[Herbener, E. S., & Harrow, M. (2004). Are Negative Symptoms Associated With Functioning Deficits in Both Schizophrenia and Nonschizophrenia Patients? A 10-Year Longitudinal Analysis. *Schizophrenia Bulletin*, *30*(4), 813–825. https://doi.org/10.1093/oxfordjournals.schbul.a007134](https://www.zotero.org/google-docs/?SJrRhI)

[Herbert, D., Neves-Pereira, M., Baidya, R., Cheema, S., Groleau, S., Shahmirian, A., Tiwari, A. K., Zai, C. C., King, N., Muller, D. J., & Kennedy, J. L. (2018). Genetic testing as a supporting tool in prescribing psychiatric medication: Design and protocol of the IMPACT study. *J Psychiatr Res*, *96*, 265–272. https://doi.org/10.1016/j.jpsychires.2017.09.002](https://www.zotero.org/google-docs/?SJrRhI)

Hill, S.K., Sweeney, J.A., Hamer, R.M, Keefe, R.S.E., Perkins, D.O., Gu, H., McEvoy, J.P., & Lieberman, J.A. (2008). Efficiency of the CATIE and BACS neuropsychological batteries in assessing cognitive effects of antipsychotic treatments in schizophrenia. *Journal of the International Neuropsychological Society, 14 (20),* 209-221.

[Hillborg, H., Lövgren, V., Bejerholm, U., & Rosenberg, D. (2020). Integrating Interventions That Can Support a Career-Oriented Recovery for Young Adults: Building on the Supported Education Knowledge Base. *Journal of Psychosocial Rehabilitation and Mental Health*. https://doi.org/10.1007/s40737-020-00197-2](https://www.zotero.org/google-docs/?SJrRhI)

[Ho, B. C., Nopoulos, P., Flaum, M., Arndt, S., & Andreasen, N. C. (1998). Two-year outcome in first-episode schizophrenia: Predictive value of symptoms for quality of life. *The American Journal of Psychiatry*, *155*(9), 1196–1201.](https://www.zotero.org/google-docs/?SJrRhI)

[Ho, B.-C., Andreasen, N. C., Nopoulos, P., Arndt, S., Magnotta, V., & Flaum, M. (2003). Progressive structural brain abnormalities and their relationship to clinical outcome: A longitudinal magnetic resonance imaging study early in schizophrenia. *Archives of General Psychiatry*, *60*(6), 585–594. https://doi.org/10.1001/archpsyc.60.6.585](https://www.zotero.org/google-docs/?SJrRhI)

[Hodgekins, J., Birchwood, M., Christopher, R., Marshall, M., Coker, S., Everard, L., Lester, H., Jones, P., Amos, T., Singh, S., Sharma, V., Freemantle, N., & Fowler, D. (2015). Investigating trajectories of social recovery in individuals with first-episode psychosis: A latent class growth analysis. *The British Journal of Psychiatry*, *207*(6), 536–543. https://doi.org/10.1192/bjp.bp.114.153486](https://www.zotero.org/google-docs/?SJrRhI)

[Hoffmann, H., Jäckel, D., Glauser, S., Mueser, K. T., & Kupper, Z. (2014). Long-term effectiveness of supported employment: 5-year follow-up of a randomized controlled trial. *The American Journal of Psychiatry*, *171*(11), 1183–1190. https://doi.org/10.1176/appi.ajp.2014.13070857](https://www.zotero.org/google-docs/?SJrRhI)

[Hogan, T. P., Awad, A. G., & Eastwood, R. (1983). A self-report scale predictive of drug compliance in schizophrenics: Reliability and discriminative validity. *Psychol Med*, *13*(1), 177–183. https://doi.org/10.1017/s0033291700050182](https://www.zotero.org/google-docs/?SJrRhI)

[Holt, R. I. G., Gossage-Worrall, R., Hind, D., Bradburn, M. J., McCrone, P., Morris, T., Edwardson, C., Barnard, K., Carey, M. E., Davies, M. J., Dickens, C. M., Doherty, Y., Etherington, A., French, P., Gaughran, F., Greenwood, K. E., Kalidindi, S., Khunti, K., Laugharne, R., … Wright, S. (2019). Structured lifestyle education for people with schizophrenia, schizoaffective disorder and first-episode psychosis (STEPWISE): Randomised controlled trial. *Br J Psychiatry*, *214*(2), 63–73. https://doi.org/10.1192/bjp.2018.167](https://www.zotero.org/google-docs/?SJrRhI)

Honan, C. A., McDonald, S., Sufani, C., Hine, D. W., & Kumfor, F. (2016). The awareness of social inference test: Development of a shortened version for use in adults with acquired brain injury. *The Clinical Neuropsychologist*, *30*(2), 243-264. <https://doi.org/10.1080/13854046.2015.1136691>

[Hovington, C. L., Bodnar, M., Joober, R., Malla, A. K., & Lepage, M. (2012). Identifying persistent negative symptoms in first episode psychosis. *BMC Psychiatry*, *12*, 224. https://doi.org/10.1186/1471-244X-12-224](https://www.zotero.org/google-docs/?SJrRhI)

[Howes, O. D., McCutcheon, R., Agid, O., de Bartolomeis, A., van Beveren, N. J. M., Birnbaum, M. L., Bloomfield, M. A. P., Bressan, R. A., Buchanan, R. W., Carpenter, W. T., Castle, D. J., Citrome, L., Daskalakis, Z. J., Davidson, M., Drake, R. J., Dursun, S., Ebdrup, B. H., Elkis, H., Falkai, P., … Correll, C. U. (2017, March 1). *Treatment-Resistant Schizophrenia: Treatment Response and Resistance in Psychosis (TRRIP) Working Group Consensus Guidelines on Diagnosis and Terminology.* [Consensus Development Conference]. The American journal of psychiatry.](https://www.zotero.org/google-docs/?SJrRhI)

[Hu, H. H., Chen, J., & Shen, W. (2016). Segmentation and quantification of adipose tissue by magnetic resonance imaging. *Magma (New York, N.Y.)*, *29*(2), 259–276. https://doi.org/10.1007/s10334-015-0498-z](https://www.zotero.org/google-docs/?SJrRhI)

[Huhn, M., Nikolakopoulou, A., Schneider-Thoma, J., Krause, M., Samara, M., Peter, N., Arndt, T., Bäckers, L., Rothe, P., Cipriani, A., Davis, J., Salanti, G., & Leucht, S. (2019). Comparative efficacy and tolerability of 32 oral antipsychotics for the acute treatment of adults with multi-episode schizophrenia: A systematic review and network meta-analysis. *Lancet (London, England)*, *394*(10202), 939–951. https://doi.org/10.1016/S0140-6736(19)31135-3](https://www.zotero.org/google-docs/?SJrRhI)

[Hurford, I. M., Marder, S. R., Keefe, R. S. E., Reise, S. P., & Bilder, R. M. (2011). A brief cognitive assessment tool for schizophrenia: Construction of a tool for clinicians. *Schizophrenia Bulletin*, *37*(3), 538–545. https://doi.org/10.1093/schbul/sbp095](https://www.zotero.org/google-docs/?SJrRhI)

[International Schizophrenia Consortium, Purcell, S. M., Wray, N. R., Stone, J. L., Visscher, P. M., O’Donovan, M. C., Sullivan, P. F., & Sklar, P. (2009). Common polygenic variation contributes to risk of schizophrenia and bipolar disorder. *Nature*, *460*(7256), 748–752. https://doi.org/10.1038/nature08185](https://www.zotero.org/google-docs/?SJrRhI)

[Iqbal, E., Mallah, R., Jackson, R. G., Ball, M., Ibrahim, Z. M., Broadbent, M., Dzahini, O., Stewart, R., Johnston, C., & Dobson, R. J. (2015). Identification of Adverse Drug Events from Free Text Electronic Patient Records and Information in a Large Mental Health Case Register. *PLoS ONE*, *10*(8), e0134208. https://doi.org/10.1371/journal.pone.0134208](https://www.zotero.org/google-docs/?SJrRhI)

[Iwata, Y., Nakajima, S., Suzuki, T., Keefe, R. S. E., Plitman, E., Chung, J. K., Caravaggio, F., Mimura, M., Graff-Guerrero, A., & Uchida, H. (2015). Effects of glutamate positive modulators on cognitive deficits in schizophrenia: A systematic review and meta-analysis of double-blind randomized controlled trials. *Molecular Psychiatry*, *20*(10), 1151–1160. https://doi.org/10.1038/mp.2015.68](https://www.zotero.org/google-docs/?SJrRhI)

[Jaaskelainen, E., Juola, P., Hirvonen, N., McGrath, J. J., Saha, S., Isohanni, M., Veijola, J., & Miettunen, J. (2013). A Systematic Review and Meta-Analysis of Recovery in Schizophrenia. *Schizophrenia Bulletin*, *39*(6), 1296–1306. https://doi.org/10.1093/schbul/sbs130](https://www.zotero.org/google-docs/?SJrRhI)

[Jabben, N., Arts, B., Os, J. van, & Krabbendam, L. (2010). Neurocognitive Functioning as Intermediary Phenotype and Predictor of Psychosocial Functioning Across the Psychosis Continuum: Studies in Schizophrenia and Bipolar Disorder. *The Journal of Clinical Psychiatry*, *71*(6), 764–774. https://doi.org/10.4088/JCP.08m04837yel](https://www.zotero.org/google-docs/?SJrRhI)

[Janowitz, D., Wittfeld, K., Terock, J., Freyberger, H. J., Hegenscheid, K., Völzke, H., Habes, M., Hosten, N., Friedrich, N., Nauck, M., Domanska, G., & Grabe, H. J. (2015). Association between waist circumference and gray matter volume in 2344 individuals from two adult community-based samples. *NeuroImage*, *122*. https://doi.org/10.1016/j.neuroimage.2015.07.086](https://www.zotero.org/google-docs/?SJrRhI)

[Jauhar, S., McKenna, P. J., Radua, J., Fung, E., Salvador, R., & Laws, K. R. (2014). Cognitive-behavioural therapy for the symptoms of schizophrenia: Systematic review and meta-analysis with examination of potential bias. *Br J Psychiatry*, *204*(1), 20–29. https://doi.org/10.1192/bjp.bp.112.116285](https://www.zotero.org/google-docs/?SJrRhI)

[Jemal, A., Ward, E., Hao, Y., & Thun, M. (2005). Trends in the leading causes of death in the United States, 1970-2002. *Journal of the American Medical Association*, *294*(10). https://doi.org/10.1001/jama.294.10.1255](https://www.zotero.org/google-docs/?SJrRhI)

[Johnstone, E. C., Cosway, R., & Lawrie, S. M. (2002). Distinguishing characteristics of subjects with good and poor early outcome in the Edinburgh High-Risk Study. *The British Journal of Psychiatry. Supplement*, *43*, s26-29. https://doi.org/10.1192/bjp.181.43.s26](https://www.zotero.org/google-docs/?SJrRhI)

[Jones, P. B., Barnes, T. R. E., Davies, L., Dunn, G., Lloyd, H., Hayhurst, K. P., Murray, R. M., Markwick, A., & Lewis, S. W. (2006). Randomized controlled trial of the effect on Quality of Life of second- vs first-generation antipsychotic drugs in schizophrenia: Cost Utility of the Latest Antipsychotic Drugs in Schizophrenia Study (CUtLASS 1). *Archives of General Psychiatry*, *63*(10), 1079–1087. https://doi.org/10.1001/archpsyc.63.10.1079](https://www.zotero.org/google-docs/?SJrRhI)

[Jongsma, H. E., Gayer-Anderson, C., Lasalvia, A., Quattrone, D., Mulè, A., Szöke, A., Selten, J.-P., Turner, C., Arango, C., Tarricone, I., Berardi, D., Tortelli, A., Llorca, P.-M., de Haan, L., Bobes, J., Bernardo, M., Sanjuán, J., Santos, J. L., Arrojo, M., … European Network of National Schizophrenia Networks Studying Gene-Environment Interactions Work Package 2 (EU-GEI WP2) Group. (2018). Treated Incidence of Psychotic Disorders in the Multinational EU-GEI Study. *JAMA Psychiatry*, *75*(1), 36–46. https://doi.org/10.1001/jamapsychiatry.2017.3554](https://www.zotero.org/google-docs/?SJrRhI)

[Kambeitz-Ilankovic, L., Betz, L. T., Dominke, C., Haas, S. S., Subramaniam, K., Fisher, M., Vinogradov, S., Koutsouleris, N., & Kambeitz, J. (2019). *Multi-outcome meta-analysis (MOMA) of cognitive remediation in schizophrenia: Revisiting the relevance of human coaching and elucidating interplay between multiple outcomes*.](https://www.zotero.org/google-docs/?SJrRhI)

[Kane, J., Honigfeld, G., Singer, J., & Meltzer, H. (1988). Clozapine for the Treatment-Resistant Schizophrenic: A Double-blind Comparison With Chlorpromazine. *Archives of General Psychiatry*, *45*(9), 789–796. https://doi.org/10.1001/archpsyc.1988.01800330013001](https://www.zotero.org/google-docs/?SJrRhI)

[Kashner, T. M., Stensland, M. D., Lind, L., Wicker, A., Rush, A. J., Golden, R. M., & Henley, S. S. (2009). Measuring use and cost of care for patients with mood disorders: The utilization and cost inventory. *Medical Care*, *47*(2), 184–190. https://doi.org/10.1097/MLR.0b013e31818457b8](https://www.zotero.org/google-docs/?SJrRhI)

[Kasparek, T., Prikryl, R., Schwarz, D., Kucerova, H., Marecek, R., Mikl, M., Vanicek, J., & Ceskova, E. (2009). Gray matter morphology and the level of functioning in one-year follow-up of first-episode schizophrenia patients. *Progress in Neuro-Psychopharmacology & Biological Psychiatry*, *33*(8), 1438–1446. https://doi.org/10.1016/j.pnpbp.2009.07.025](https://www.zotero.org/google-docs/?SJrRhI)

Keefe, R.S.E., Bilder, R.M., Davis, S.M., Harvey, P.D., Palmer, B.W., Gold, J.M., Meltzer, M.F., Green, M.F., Capuano, G., Stroup, T.S., McEvoy, J.P., McEvoy, M.S., Swarts, R.A., Rosenheck, D.O., Perkins, C.E., Davis, J.K., Hsiao, J.A., Lieberman (2007). Neurocognitive effects of antipsychotic medications in patients with chronic schizophrenia in the CATIE trial. *Archives of General Psychiatry, 64 (6),* 633-647.

Keefe, R.S.E., Goldberg, T. E., Harvey, P. D., Gold, J. M., Poe, M. P., & Coughenour, L. (2004). The Brief Assessment of Cognition in Schizophrenia: reliability, sensitivity, and comparison with a standard neurocognitive battery. *Schizophrenia research*, *68*(2-3), 283–297. https://doi.org/10.1016/j.schres.2003.09.011

[Keefe, R. S. E., Haig, G. M., Marder, S. R., Harvey, P. D., Dunayevich, E., Medalia, A., Davidson, M., Lombardo, I., Bowie, C. R., Buchanan, R. W., Bugarski-Kirola, D., Carpenter, W. T., Csernansky, J. T., Dago, P. L., Durand, D. M., Frese, F. J., Goff, D. C., Gold, J. M., Hooker, C. I., … Stern, R. G. (2016). Report on ISCTM Consensus Meeting on Clinical Assessment of Response to Treatment of Cognitive Impairment in Schizophrenia. *Schizophrenia Bulletin*, *42*(1). https://doi.org/10.1093/schbul/sbv111](https://www.zotero.org/google-docs/?SJrRhI)

[Keefe, R. S. E., Sweeney, J. A., Gu, H., Hamer, R. M., Perkins, D. O., McEvoy, J. P., & Lieberman, J. A. (2007). Effects of olanzapine, quetiapine, and risperidone on neurocognitive function in early psychosis: A randomized, double-blind 52-week comparison. *American Journal of Psychiatry*, *164*(7). https://doi.org/10.1176/ajp.2007.164.7.1061](https://www.zotero.org/google-docs/?SJrRhI)

[Kern, R. S., Gold, J. M., Dickinson, D., Green, M. F., Nuechterlein, K. H., Baade, L. E., Keefe, R. S. E., Mesholam-Gately, R. I., Seidman, L. J., Lee, C., Sugar, C. A., & Marder, S. R. (2011). The MCCB impairment profile for schizophrenia outpatients: Results from the MATRICS psychometric and standardization study. *Schizophrenia Research*, *126*(1–3), 124–131. https://doi.org/10.1016/j.schres.2010.11.008](https://www.zotero.org/google-docs/?SJrRhI)

[Kidd, S. A., Herman, Y., Barbic, S., Ganguli, R., George, T. P., Hassan, S., McKenzie, K., Maples, N., & Velligan, D. (2014). Testing a modification of cognitive adaptation training: Streamlining the model for broader implementation. *Schizophrenia Research*, *156*(1), 46–50. https://doi.org/10.1016/j.schres.2014.03.026](https://www.zotero.org/google-docs/?SJrRhI)

[Kidd, S. A., Kerman, N., Ernest, D., Maples, N., Arthur, C., de Souza, S., Kath, J., Herman, Y., Virdee, G., Collins, A., & Velligan, D. (2018). A pilot study of a family cognitive adaptation training guide for individuals with schizophrenia. *Psychiatric Rehabilitation Journal*, *41*(2), 109–117. https://doi.org/10.1037/prj0000204](https://www.zotero.org/google-docs/?SJrRhI)

[Kidd, S. A., & Kral, M. J. (2005). Practicing participatory action research. *Journal of Counseling Psychology*, *52*(2), 187–195. https://doi.org/10.1037/0022-0167.52.2.187](https://www.zotero.org/google-docs/?SJrRhI)

[Kim, K. R., Song, Y. Y., Park, J. Y., Lee, E. H., Lee, M., Lee, S. Y., Kang, J. I., Lee, E., Yoo, S. W., An, S. K., & Kwon, J. S. (2013). The relationship between psychosocial functioning and resilience and negative symptoms in individuals at ultra-high risk for psychosis. *Australian & New Zealand Journal of Psychiatry*, *47*(8), 762–771. https://doi.org/10.1177/0004867413488218](https://www.zotero.org/google-docs/?SJrRhI)

[Kinoshita, Y., Furukawa, T. A., Kinoshita, K., Honyashiki, M., Omori, I. M., Marshall, M., Bond, G. R., Huxley, P., Amano, N., & Kingdon, D. (2013). Supported employment for adults with severe mental illness. *The Cochrane Database of Systematic Reviews*, *9*, CD008297. https://doi.org/10.1002/14651858.CD008297.pub2](https://www.zotero.org/google-docs/?SJrRhI)

[Kirkbride, J., Coid, J. W., Morgan, C., Fearon, P., Dazzan, P., Yang, M., Lloyd, T., Harrison, G. L., Murray, R. M., & Jones, P. B. (2010). Translating the epidemiology of psychosis into public mental health: Evidence, challenges and future prospects. *Journal of Public Mental Health*, *9*(2), 4–14. https://doi.org/10.5042/jpmh.2010.0324](https://www.zotero.org/google-docs/?SJrRhI)

[Klein, P., Lawn, S., Tsourtos, G., & Agteren, J. van. (2019). Tailoring of a Smartphone Smoking Cessation App (Kick.it) for Serious Mental Illness Populations: Qualitative Study. *JMIR Human Factors*, *6*(3), e14023. https://doi.org/10.2196/14023](https://www.zotero.org/google-docs/?SJrRhI)

[Kohler, C. G., Bilker, W., Hagendoorn, M., Gur, R. E., & Gur, R. C. (2000). Emotion recognition deficit in schizophrenia: Association with symptomatology and cognition. *Biol Psychiatry*, *48*(2), 127–136. https://doi.org/10.1016/s0006-3223(00)00847-7](https://www.zotero.org/google-docs/?SJrRhI)

[Kolenic, M., Franke, K., Hlinka, J., Matejka, M., Capkova, J., Pausova, Z., Uher, R., Alda, M., Spaniel, F., & Hajek, T. (2018). Obesity, dyslipidemia and brain age in first-episode psychosis. *Journal of Psychiatric Research*, *99*. https://doi.org/10.1016/j.jpsychires.2018.02.012](https://www.zotero.org/google-docs/?SJrRhI)

[Kolotkin, R. L., Crosby, R. D., Corey-Lisle, P. K., Li, H., & Swanson, J. M. (2006). Performance of a weight-related measure of quality of life in a psychiatric sample. *Quality of Life Research*, *15*(4). https://doi.org/10.1007/s11136-005-4627-4](https://www.zotero.org/google-docs/?SJrRhI)

[Kotov, R., Fochtmann, L., Li, K., Tanenberg-Karant, M., Constantino, E. A., Rubinstein, J., Perlman, G., Velthorst, E., Fett, A.-K. J., Carlson, G., & Bromet, E. J. (2017). Declining Clinical Course of Psychotic Disorders Over the Two Decades Following First Hospitalization: Evidence From the Suffolk County Mental Health Project. *The American Journal of Psychiatry*, *174*(11), 1064–1074. https://doi.org/10.1176/appi.ajp.2017.16101191](https://www.zotero.org/google-docs/?SJrRhI)

[Koutsouleris, N., Dwyer, D. B., Degenhardt, F., Maj, C., Urquijo-Castro, M. F., Sanfelici, R., Popovic, D., Oeztuerk, O., Haas, S. S., Weiske, J., Ruef, A., Kambeitz-Ilankovic, L., Antonucci, L. A., Neufang, S., Schmidt-Kraepelin, C., Ruhrmann, S., Penzel, N., Kambeitz, J., Haidl, T. K., … Consortium, P. (2020). Multimodal Machine Learning Workflows for Prediction of Psychosis in Patients with Clinical High-Risk Syndromes and Recent-Onset Depression. *JAMA Psychiatry*. https://doi.org/10.1001/jamapsychiatry.2020.3604](https://www.zotero.org/google-docs/?SJrRhI)

[Kowalec, K., Lu, Y., Sariaslan, A., Song, J., Ploner, A., Dalman, C., Hultman, C. M., Larsson, H., Lichtenstein, P., & Sullivan, P. F. (2019). Increased schizophrenia family history burden and reduced premorbid IQ in treatment-resistant schizophrenia: A Swedish National Register and Genomic Study. *Mol Psychiatry*. https://doi.org/10.1038/s41380-019-0575-1](https://www.zotero.org/google-docs/?SJrRhI)

[Kring, A. M., Gur, R. E., Blanchard, J. J., Horan, W. P., & Reise, S. P. (2013). The Clinical Assessment Interview for Negative Symptoms (CAINS): Final development and validation. *The American Journal of Psychiatry*, *170*(2), 165–172. https://doi.org/10.1176/appi.ajp.2012.12010109](https://www.zotero.org/google-docs/?SJrRhI)

[Kuk, J. L., Church, T. S., Blair, S. N., & Ross, R. (2006). Does measurement site for visceral and abdominal subcutaneous adipose tissue alter associations with the metabolic syndrome? *Diabetes Care*, *29*(3), 679–684. https://doi.org/10.2337/diacare.29.03.06.dc05-1500](https://www.zotero.org/google-docs/?SJrRhI)

[Kukla, M., Bond, G. R., & Xie, H. (2012). A Prospective Investigation of Work and Nonvocational Outcomes in Adults With Severe Mental Illness. *The Journal of Nervous and Mental Disease*, *200*(3), 214–222. https://doi.org/10.1097/NMD.0b013e318247cb29](https://www.zotero.org/google-docs/?SJrRhI)

[Kullmann, S., Heni, M., Hallschmid, M., Fritsche, A., Preissl, H., & Häring, H. U. (2016). Brain insulin resistance at the crossroads of metabolic and cognitive disorders in humans. *Physiological Reviews*, *96*(4). https://doi.org/10.1152/physrev.00032.2015](https://www.zotero.org/google-docs/?SJrRhI)

[Kurdyak, P., Lin, E., Green, D., & Vigod, S. (2015). Validation of a Population-Based Algorithm to Detect Chronic Psychotic Illness. *Canadian Journal of Psychiatry. Revue Canadienne de Psychiatrie*, *60*(8), 362–368.](https://www.zotero.org/google-docs/?SJrRhI)

[Kurimori, M., Shiozawa, P., Bikson, M., Aboseria, M., & Cordeiro, Q. (2015). Targeting negative symptoms in schizophrenia: Results from a proof-of-concept trial assessing prefrontal anodic tDCS protocol. *Schizophrenia Research*, *166*(1–3), 362–363. https://doi.org/10.1016/j.schres.2015.05.029](https://www.zotero.org/google-docs/?SJrRhI)

[Lally, J., Tully, J., Robertson, D., Stubbs, B., Gaughran, F., & MacCabe, J. H. (2016). Augmentation of clozapine with electroconvulsive therapy in treatment resistant schizophrenia: A systematic review and meta-analysis. *Schizophrenia Research*, *171*(1–3), 215–224. https://doi.org/10.1016/j.schres.2016.01.024](https://www.zotero.org/google-docs/?SJrRhI)

[Lambert, T. J. R., Reavley, N. J., Jorm, A. F., & Oakley Browne, M. A. (2017). Royal Australian and New Zealand College of Psychiatrists expert consensus statement for the treatment, management and monitoring of the physical health of people with an enduring psychotic illness. *Australian and New Zealand Journal of Psychiatry*, *51*(4). https://doi.org/10.1177/0004867416686693](https://www.zotero.org/google-docs/?SJrRhI)

[Lane, R. D., Glazer, W. M., Hansen, T. E., Berman, W. H., & Kramer, S. I. (1985). Assessment of tardive dyskinesia using the Abnormal Involuntary Movement Scale. *The Journal of Nervous and Mental Disease*, *173*(6), 353–357.](https://www.zotero.org/google-docs/?SJrRhI)

[Latimer, E. A., Lecomte, T., Becker, D. R., Drake, R. E., Duclos, I., Piat, M., Lahaie, N., St-Pierre, M.-S., Therrien, C., & Xie, H. (2006). Generalisability of the individual placement and support model of supported employment: Results of a Canadian randomised controlled trial. *The British Journal of Psychiatry*, *189*(1), 65–73. https://doi.org/10.1192/bjp.bp.105.012641](https://www.zotero.org/google-docs/?SJrRhI)

[Law, H., & Morrison, A. P. (2014). Recovery in psychosis: A Delphi study with experts by experience. *Schizophr Bull*, *40*(6), 1347–1355. https://doi.org/10.1093/schbul/sbu047](https://www.zotero.org/google-docs/?SJrRhI)

[Law, Heather, Shryane, N., Bentall, R. P., & Morrison, A. P. (2016). Longitudinal predictors of subjective recovery in psychosis. *The British Journal of Psychiatry*, *209*(1), 48–53. https://doi.org/10.1192/bjp.bp.114.158428](https://www.zotero.org/google-docs/?SJrRhI)

[Laws, K. R., Darlington, N., Kondel, T. K., McKenna, P. J., & Jauhar, S. (2018). Cognitive Behavioural Therapy for schizophrenia—Outcomes for functioning, distress and quality of life: A meta-analysis. *BMC Psychol*, *6*(1), 32. https://doi.org/10.1186/s40359-018-0243-2](https://www.zotero.org/google-docs/?SJrRhI)

[Leamy, M., Bird, V., Le Boutillier, C., Williams, J., & Slade, M. (2011). Conceptual framework for personal recovery in mental health: Systematic review and narrative synthesis. *Br J Psychiatry*, *199*(6), 445–452. https://doi.org/10.1192/bjp.bp.110.083733](https://www.zotero.org/google-docs/?SJrRhI)

[Lecomte, T., Corbière, M., & Laisné, F. (2006). Investigating self-esteem in individuals with schizophrenia: Relevance of the Self-Esteem Rating Scale-Short Form. *Psychiatry Research*, *143*(1), 99–108. https://doi.org/10.1016/j.psychres.2005.08.019](https://www.zotero.org/google-docs/?SJrRhI)

[Leifker, F. R., Bowie, C. R., & Harvey, P. D. (2009). Determinants of everyday outcomes in schizophrenia: The influences of cognitive impairment, functional capacity, and symptoms. *Schizophrenia Research*, *115*(1), 82–87. https://doi.org/10.1016/j.schres.2009.09.004](https://www.zotero.org/google-docs/?SJrRhI)

[Lent-Schochet, D., McLaughlin, M., Ramakrishnan, N., & Jialal, I. (2019). Exploratory metabolomics of metabolic syndrome: A status report. *World Journal of Diabetes*, *10*(1), 23–36. https://doi.org/10.4239/wjd.v10.i1.23](https://www.zotero.org/google-docs/?SJrRhI)

[Leucht, C., Heres, S., Kane, J. M., Kissling, W., Davis, J. M., & Leucht, S. (2011). Oral versus depot antipsychotic drugs for schizophrenia—A critical systematic review and meta-analysis of randomised long-term trials. *Schizophrenia Research*, *127*(1), 83–92. https://doi.org/10.1016/j.schres.2010.11.020](https://www.zotero.org/google-docs/?SJrRhI)

[Leucht, S., Cipriani, A., Spineli, L., Mavridis, D., Örey, D., Richter, F., Samara, M., Barbui, C., Engel, R. R., Geddes, J. R., Kissling, W., Stapf, M. P., Lässig, B., Salanti, G., & Davis, J. M. (2013). Comparative efficacy and tolerability of 15 antipsychotic drugs in schizophrenia: A multiple-treatments meta-analysis. *The Lancet*, *382*(9896), 951–962. https://doi.org/10.1016/S0140-6736(13)60733-3](https://www.zotero.org/google-docs/?SJrRhI)

[Leucht, S., & Davis, J. M. (2017). Do antipsychotic drugs lose their efficacy for relapse prevention over time? *The British Journal of Psychiatry*, *211*(3), 127–129. https://doi.org/10.1192/bjp.bp.117.201103](https://www.zotero.org/google-docs/?SJrRhI)

[Leucht, S., Leucht, C., Huhn, M., Chaimani, A., Mavridis, D., Helfer, B., Samara, M., Rabaioli, M., Bächer, S., Cipriani, A., Geddes, J. R., Salanti, G., & Davis, J. M. (2017). Sixty Years of Placebo-Controlled Antipsychotic Drug Trials in Acute Schizophrenia: Systematic Review, Bayesian Meta-Analysis, and Meta-Regression of Efficacy Predictors. *The American Journal of Psychiatry*, *174*(10), 927–942. https://doi.org/10.1176/appi.ajp.2017.16121358](https://www.zotero.org/google-docs/?SJrRhI)

[Leucht, S., Tardy, M., Komossa, K., Heres, S., Kissling, W., & Davis, J. M. (2012). Maintenance treatment with antipsychotic drugs for schizophrenia. *The Cochrane Database of Systematic Reviews*, *5*, CD008016. https://doi.org/10.1002/14651858.CD008016.pub2](https://www.zotero.org/google-docs/?SJrRhI)

[Leucht, S., Tardy, M., Komossa, K., Heres, S., Kissling, W., Salanti, G., & Davis, J. M. (2012). Antipsychotic drugs versus placebo for relapse prevention in schizophrenia: A systematic review and meta-analysis. *The Lancet*, *379*(9831), 2063–2071. https://doi.org/10.1016/S0140-6736(12)60239-6](https://www.zotero.org/google-docs/?SJrRhI)

[Leung, W. W., Bowie, C. R., & Harvey, P. D. (2008). Functional implications of neuropsychological normality and symptom remission in older outpatients diagnosed with schizophrenia: A cross-sectional study. *Journal of the International Neuropsychological Society: JINS*, *14*(3), 479–488. https://doi.org/10.1017/S1355617708080600](https://www.zotero.org/google-docs/?SJrRhI)

[Lewis, S. W., Barnes, T. R., Davies, L., Murray, R. M., Dunn, G., Hayhurst, K. P., Markwick, A., Lloyd, H., & Jones, P. B. (2006). Randomized controlled trial of effect of prescription of clozapine versus other second-generation antipsychotic drugs in resistant schizophrenia. *Schizophrenia Bulletin*, *32*(4), 715–723.](https://www.zotero.org/google-docs/?SJrRhI)

[Lexén, A., & Bejerholm, U. (2018). Occupational engagement and cognitive functioning among persons with schizophrenia: An explorative study. *Scandinavian Journal of Occupational Therapy*, *25*(3), 172–179. https://doi.org/10.1080/11038128.2017.1290135](https://www.zotero.org/google-docs/?SJrRhI)

[Li, C., Wang, A., Wang, C., Ramamurthy, J., Zhang, E., Guadagno, E., & Trakadis, Y. (2018). Metabolomics in patients with psychosis: A systematic review. *American Journal of Medical Genetics Part B: Neuropsychiatric Genetics*, *177*(6), 580–588. https://doi.org/10.1002/ajmg.b.32662](https://www.zotero.org/google-docs/?SJrRhI)

[Li, T., Stefansson, H., Gudfinnsson, E., Cai, G., Liu, X., Murray, R. M., Steinthorsdottir, V., Januel, D., Gudnadottir, V. G., Petursson, H., Ingason, A., Gulcher, J. R., Stefansson, K., & Collier, D. A. (2004). Identification of a novel neuregulin 1 at-risk haplotype in Han schizophrenia Chinese patients, but no association with the Icelandic/Scottish risk haplotype. *Mol Psychiatry*, *9*(7), 698–704. https://doi.org/10.1038/sj.mp.4001485](https://www.zotero.org/google-docs/?SJrRhI)

[Lieberman, J. A., Stroup, T. S., McEvoy, J. P., Swartz, M. S., Rosenheck, R. A., Perkins, D. O., Keefe, R. S. E., Davis, S. M., Davis, C. E., Lebowitz, B. D., Severe, J., & Hsiao, J. K. (2005). Effectiveness of Antipsychotic Drugs in Patients with Chronic Schizophrenia. *New England Journal of Medicine*, *353*(12), 1209–1223. https://doi.org/10.1056/NEJMoa051688](https://www.zotero.org/google-docs/?SJrRhI)

[Lingjaerde, O., Ahlfors, U. G., Bech, P., Dencker, S. J., & Elgen, K. (1987). The UKU side effect rating scale. A new comprehensive rating scale for psychotropic drugs and a cross-sectional study of side effects in neuroleptic-treated patients. *Acta Psychiatrica Scandinavica Supplementum*, *334*, 1–100.](https://www.zotero.org/google-docs/?SJrRhI)

[Lloyd, C., King, R., & Moore, L. (2010). Subjective and objective indicators of recovery in severe mental illness: A cross-sectional study. *The International Journal of Social Psychiatry*, *56*(3), 220–229. https://doi.org/10.1177/0020764009105703](https://www.zotero.org/google-docs/?SJrRhI)

[Luther, L., Suor, J. H., Rosen, C., Jobe, T. H., Faull, R. N., & Harrow, M. (2020). Clarifying the direction of impact of negative symptoms and neurocognition on prospective work functioning in psychosis: A 20-year longitudinal study. *Schizophrenia Research*, *220*, 232–239. https://doi.org/10.1016/j.schres.2020.03.012](https://www.zotero.org/google-docs/?SJrRhI)

[Lysaker, P. H., Davis, L. W., Warman, D. M., Strasburger, A., & Beattie, N. (2007). Stigma, social function and symptoms in schizophrenia and schizoaffective disorder: Associations across 6 months. *Psychiatry Research*, *149*(1), 89–95. https://doi.org/10.1016/j.psychres.2006.03.007](https://www.zotero.org/google-docs/?SJrRhI)

[Maayan, L., Vakhrusheva, J., & Correll, C. U. (2010). Effectiveness of medications used to attenuate antipsychotic-related weight gain and metabolic abnormalities: A systematic review and meta-analysis. *Neuropsychopharmacology*, *35*(7), 1520–1530. https://doi.org/10.1038/npp.2010.21](https://www.zotero.org/google-docs/?SJrRhI)

[MacKenzie, N. E., Kowalchuk, C., Agarwal, S. M., Costa-Dookhan, K. A., Caravaggio, F., Gerretsen, P., Chintoh, A., Remington, G. J., Taylor, V. H., Mueller, D. J., Graff-Guerrero, A., & Hahn, M. K. (2018). Antipsychotics, Metabolic Adverse Effects, and Cognitive Function in Schizophrenia. *Front Psychiatry*, *9*, 622. https://doi.org/10.3389/fpsyt.2018.00622](https://www.zotero.org/google-docs/?SJrRhI)

[Malhotra, N., Kulhara, P., Chakrabarti, S., & Grover, S. (2016). Lifestyle related factors & impact of metabolic syndrome on quality of life, level of functioning & self-esteem in patients with bipolar disorder & schizophrenia. *Indian Journal of Medical Research*, *143*(April). https://doi.org/10.4103/0971-5916.184284](https://www.zotero.org/google-docs/?SJrRhI)

[Mansur, R. B., Ahmed, J., Cha, D. S., Woldeyohannes, H. O., Subramaniapillai, M., Lovshin, J., Lee, J. G., Lee, J. H., Brietzke, E., Reininghaus, E. Z., Sim, K., Vinberg, M., Rasgon, N., Hajek, T., & McIntyre, R. S. (2017). Liraglutide promotes improvements in objective measures of cognitive dysfunction in individuals with mood disorders: A pilot, open-label study. *J Affect Disord*, *207*, 114–120. https://doi.org/10.1016/j.jad.2016.09.056](https://www.zotero.org/google-docs/?SJrRhI)

[Maples, N. J., & Velligan, D. I. (2008). Cognitive Adaptation Training: Establishing Environmental Supports to Bypass Cognitive Deficits and Improve Functional Outcomes. *American Journal of Psychiatric Rehabilitation*, *11*(2), 164–180. https://doi.org/10.1080/15487760801963686](https://www.zotero.org/google-docs/?SJrRhI)

[Martin, A. K., & Mowry, B. (2016). Increased rare duplication burden genomewide in patients with treatment-resistant schizophrenia. *Psychol Med*, *46*(3), 469–476. https://doi.org/10.1017/S0033291715001701](https://www.zotero.org/google-docs/?SJrRhI)

[Martin, A. R., Kanai, M., Kamatani, Y., Okada, Y., Neale, B. M., & Daly, M. J. (2019). Clinical use of current polygenic risk scores may exacerbate health disparities. *Nature Genetics*, *51*(4), 584–591. https://doi.org/10.1038/s41588-019-0379-x](https://www.zotero.org/google-docs/?SJrRhI)

[Marwaha, S., & Johnson, S. (2004). Schizophrenia andemployment. *Social Psychiatry and Psychiatric Epidemiology*, *39*(5), 337–349. https://doi.org/10.1007/s00127-004-0762-4](https://www.zotero.org/google-docs/?SJrRhI)

[Mas-Expósito, L., Amador-Campos, J. A., Gómez-Benito, J., & Lalucat-Jo, L. (2011). The World Health Organization Quality of Life Scale Brief Version: A validation study in patients with schizophrenia. *Quality of Life Research*, *20*(7), 1079–1089. https://doi.org/10.1007/s11136-011-9847-1](https://www.zotero.org/google-docs/?SJrRhI)

[Matheson, F. I., Dunn, J. R., Smith, K. L. W., Moineddin, R., & Glazier, R. H. (2012). Development of the Canadian Marginalization Index: A New Tool for the Study of Inequality. *Canadian Journal of Public Health / Revue Canadienne de Sante’e Publique*, *103*, S12–S16. JSTOR.](https://www.zotero.org/google-docs/?SJrRhI)

[Matheson, S. L., Green, M. J., Loo, C., & Carr, V. J. (2010). Quality assessment and comparison of evidence for electroconvulsive therapy and repetitive transcranial magnetic stimulation for schizophrenia: A systematic meta-review. *Schizophrenia Research*, *118*(1–3), 201–210. https://doi.org/10.1016/j.schres.2010.01.002](https://www.zotero.org/google-docs/?SJrRhI)

[Matthews, E., Cowman, M., & Denieffe, S. (2017). Using experience‐based co‐design for the development of physical activity provision in rehabilitation and recovery mental health care. *Journal of Psychiatric and Mental Health Nursing*, *24*(7), 545–552. https://doi.org/10.1111/jpm.12401](https://www.zotero.org/google-docs/?SJrRhI)

[McCoy, T. H., Castro, V. M., Snapper, L., Hart, K., Januzzi, J. L., Huffman, J. C., & Perlis, R. H. (2017). Polygenic loading for major depression is associated with specific medical comorbidity. *Translational Psychiatry*, *7*(9), e1238. https://doi.org/10.1038/tp.2017.201](https://www.zotero.org/google-docs/?SJrRhI)

[McDonald, S., Flanagan, S., & Rollins, J. (2011). *The Awareness of Social Inference Test—Revised (TASIT-R).* Pearson Assessment.](https://www.zotero.org/google-docs/?SJrRhI)

[McEvoy, J. P., Lieberman, J. A., Stroup, T. S., Davis, S. M., Meltzer, H. Y., Rosenheck, R. A., Swartz, M. S., Perkins, D. O., Keefe, R. S., Davis, C. E., Severe, J., & Hsiao, J. K. (2006). Effectiveness of clozapine versus olanzapine, quetiapine, and risperidone in patients with chronic schizophrenia who did not respond to prior atypical antipsychotic treatment. *The American Journal of Psychiatry*, *163*(4), 600–610.](https://www.zotero.org/google-docs/?SJrRhI)

[McGurk, S. R., & Mueser, K. T. (2016). Sustaining the Long-Term Effects of Supported Employment for Persons With Psychiatric Disabilities. *American Journal of Psychiatry*, *173*(10), 953–955. https://doi.org/10.1176/appi.ajp.2016.16070811](https://www.zotero.org/google-docs/?SJrRhI)

[McGurk, S. R., Mueser, K. T., DeRosa, T. J., & Wolfe, R. (2009). Work, Recovery, and Comorbidity in Schizophrenia: A Randomized Controlled Trial of Cognitive Remediation. *Schizophrenia Bulletin*, *35*(2), 319–335. https://doi.org/10.1093/schbul/sbn182](https://www.zotero.org/google-docs/?SJrRhI)

[McGurk, S. R., Mueser, K. T., Harvey, P. D., LaPuglia, R., & Marder, J. (2003). Cognitive and Symptom Predictors of Work Outcomes for Clients With Schizophrenia in Supported Employment. *Psychiatric Services*, *54*(8), 1129–1135. https://doi.org/10.1176/appi.ps.54.8.1129](https://www.zotero.org/google-docs/?SJrRhI)

[McGurk, S. R., Mueser, K. T., Xie, H., Welsh, J., Kaiser, S., Drake, R. E., Becker, D. R., Bailey, E., Fraser, G., Wolfe, R., & McHugo, G. J. (2015). Cognitive Enhancement Treatment for People With Mental Illness Who Do Not Respond to Supported Employment: A Randomized Controlled Trial. *The American Journal of Psychiatry*, *172*(9), 852–861. https://doi.org/10.1176/appi.ajp.2015.14030374](https://www.zotero.org/google-docs/?SJrRhI)

[McKay, C., Nugent, K. L., Johnsen, M., Eaton, W. W., & Lidz, C. W. (2018). A systematic review of evidence for the Clubhouse Model of psychosocial rehabilitation. *Administration and Policy in Mental Health and Mental Health Services Research*, *45*(1), 28–47. https://doi.org/10.1007/s10488-016-0760-3](https://www.zotero.org/google-docs/?SJrRhI)

[Meddings, S., McGregor, J., Roeg, W., & Shepherd, G. (2015). Recovery colleges: Quality and outcomes. *Mental Health and Social Inclusion*, *19*(4), 212–221. https://doi.org/10.1108/MHSI-08-2015-0035](https://www.zotero.org/google-docs/?SJrRhI)

[Meltzer, H. Y. (1997). Treatment-resistant schizophrenia—The role of clozapine. *Curr Med Res Opin*, *14*(1), 1–20. https://doi.org/10.1185/03007999709113338](https://www.zotero.org/google-docs/?SJrRhI)

[Menezes, N. M., Malla, A. M., Norman, R. M., Archie, S., Roy, P., & Zipursky, R. B. (2009). A multi-site Canadian perspective: Examining the functional outcome from first-episode psychosis. *Acta Psychiatrica Scandinavica*, *120*(2), 138–146. https://doi.org/10.1111/j.1600-0447.2009.01346.x](https://www.zotero.org/google-docs/?SJrRhI)

[Meyer, J. M., Nasrallah, H. A., McEvoy, J. P., Goff, D. C., Davis, S. M., Chakos, M., Patel, J. K., Keefe, R. S. E., Stroup, T. S., & Lieberman, J. A. (2005). The Clinical Antipsychotic Trials of Intervention Effectiveness (CATIE) Schizophrenia Trial: Clinical comparison of subgroups with and without the metabolic syndrome. *Schizophrenia Research*, *80*(1). https://doi.org/10.1016/j.schres.2005.07.015](https://www.zotero.org/google-docs/?SJrRhI)

[Michalska da Rocha, B., Rhodes, S., Vasilopoulou, E., & Hutton, P. (2018). Loneliness in Psychosis: A Meta-analytical Review. *Schizophrenia Bulletin*, *44*(1), 114–125. https://doi.org/10.1093/schbul/sbx036](https://www.zotero.org/google-docs/?SJrRhI)

[Milev, P., Ho, B.-C., Arndt, S., & Andreasen, N. C. (2005). Predictive values of neurocognition and negative symptoms on functional outcome in schizophrenia: A longitudinal first-episode study with 7-year follow-up. *The American Journal of Psychiatry*, *162*(3), 495–506. https://doi.org/10.1176/appi.ajp.162.3.495](https://www.zotero.org/google-docs/?SJrRhI)

[Ministry of Health and Long-Term Care. (2020). *Become an Ontario Health Team—Health Care Professionals—MOHLTC*. Government of Ontario, Ministry of Health and Long-Term Care. https://health.gov.on.ca/en/pro/programs/connectedcare/oht/default.aspx](https://www.zotero.org/google-docs/?SJrRhI)

[Miotto, R., Li, L., Kidd, B. A., & Dudley, J. T. (2016). Deep Patient: An Unsupervised Representation to Predict the Future of Patients from the Electronic Health Records. *Sci Rep*, *6*, 26094. https://doi.org/10.1038/srep26094](https://www.zotero.org/google-docs/?SJrRhI)

[Mitchell, A. J., Delaffon, V., Vancampfort, D., Correll, C. U., & De Hert, M. (2012). *Guideline concordant monitoring of metabolic risk in people treated with antipsychotic medication: Systematic review and meta-analysis of screening practices*.](https://www.zotero.org/google-docs/?SJrRhI)

[Mohamed, S., Rosenheck, R., Swartz, M., Stroup, S., Lieberman, J. A., & Keefe, R. S. E. (2008). Relationship of Cognition and Psychopathology to Functional Impairment in Schizophrenia. *American Journal of Psychiatry*, *165*(8), 978–987. https://doi.org/10.1176/appi.ajp.2008.07111713](https://www.zotero.org/google-docs/?SJrRhI)

[Möller, H.-J., Jäger, M., Riedel, M., Obermeier, M., Strauss, A., & Bottlender, R. (2010). The Munich 15-year follow-up study (MUFUSSAD) on first-hospitalized patients with schizophrenic or affective disorders: Comparison of psychopathological and psychosocial course and outcome and prediction of chronicity. *European Archives of Psychiatry and Clinical Neuroscience*, *260*(5), 367–384. https://doi.org/10.1007/s00406-010-0117-y](https://www.zotero.org/google-docs/?SJrRhI)

[Moore, S., Shiers, D., Daly, B., Mitchell, A. J., & Gaughran, F. (2015). Promoting physical health for people with schizophrenia by reducing disparities in medical and dental care. *Acta Psychiatrica Scandinavica*, *132*(2). https://doi.org/10.1111/acps.12431](https://www.zotero.org/google-docs/?SJrRhI)

[Morgan, C., Fearon, P., Lappin, J., Heslin, M., Donoghue, K., Lomas, B., Reininghaus, U., Onyejiaka, A., Croudace, T., Jones, P. B., Murray, R. M., Doody, G. A., & Dazzan, P. (2017). Ethnicity and long-term course and outcome of psychotic disorders in a UK sample: The ÆSOP-10 study. *The British Journal of Psychiatry*, *211*(2), 88–94. https://doi.org/10.1192/bjp.bp.116.193342](https://www.zotero.org/google-docs/?SJrRhI)

[Morosini, P. L., Magliano, L., Brambilla, L., Ugolini, S., & Pioli, R. (2000). Development, reliability and acceptability of a new version of the DSM-IV Social and Occupational Functioning Assessment Scale (SOFAS) to assess routine social functioning. *Acta Psychiatrica Scandinavica*, *101*(4), 323–329.](https://www.zotero.org/google-docs/?SJrRhI)

[Morrison, A. P., Shryane, N., Beck, R., Heffernan, S., Law, H., McCusker, M., & Bentall, R. P. (2013). Psychosocial and neuropsychiatric predictors of subjective recovery from psychosis. *Psychiatry Res*, *208*(3), 203–209. https://doi.org/10.1016/j.psychres.2013.05.008](https://www.zotero.org/google-docs/?SJrRhI)

[Mötteli, S., Schori, D., Schmidt, H., Seifritz, E., & Jäger, M. (2018). Utilization and Effectiveness of Home Treatment for People With Acute Severe Mental Illness: A Propensity-Score Matching Analysis of 19 Months of Observation. *Frontiers in Psychiatry*, *9*. https://doi.org/10.3389/fpsyt.2018.00495](https://www.zotero.org/google-docs/?SJrRhI)

[Mouaffak, F., Tranulis, C., Gourevitch, R., Poirier, M.-F., Douki, S., Olié, J.-P., Lôo, H., & Gourion, D. (2006). Augmentation strategies of clozapine with antipsychotics in the treatment of ultraresistant schizophrenia. *Clinical Neuropharmacology*, *29*(1), 28–33. https://doi.org/10.1097/00002826-200601000-00009](https://www.zotero.org/google-docs/?SJrRhI)

[Mowbray, C. T., Collins, M. E., Bellamy, C. D., Megivern, D. A., Bybee, D., & Szilvagyi, S. (2005). Supported Education for Adults with Psychiatric Disabilities: An Innovation for Social Work and Psychosocial Rehabilitation Practice. *Social Work*, *50*(1), 7–20. JSTOR.](https://www.zotero.org/google-docs/?SJrRhI)

[Mucci, A., Galderisi, S., Gibertoni, D., Rossi, A., Rocca, P., Bertolino, A., Aguglia, E., Amore, M., Bellomo, A., Biondi, M., Blasi, G., Brasso, C., Bucci, P., Carpiniello, B., Cuomo, A., Dell’Osso, L., Giordano, G. M., Marchesi, C., Monteleone, P., … Italian Network for Research on Psychoses. (2021). Factors Associated With Real-Life Functioning in Persons With Schizophrenia in a 4-Year Follow-up Study of the Italian Network for Research on Psychoses. *JAMA Psychiatry*. https://doi.org/10.1001/jamapsychiatry.2020.4614](https://www.zotero.org/google-docs/?SJrRhI)

[Mueser, K., Becker, D. R., Torrey, W., Xie, H., Bond, G., Drake, R., & Dain, B. (1997). Work and nonvocational domains of functioning in persons with severe mental illness: A longitudinal analysis. *The Journal of Nervous and Mental Disease*. https://doi.org/10.1097/00005053-199707000-00001](https://www.zotero.org/google-docs/?SJrRhI)

[Mujahid, M. S., Diez Roux, A. V., Morenoff, J. D., & Raghunathan, T. (2007). Assessing the measurement properties of neighborhood scales: From psychometrics to ecometrics. *American Journal of Epidemiology*, *165*(8), 858–867. https://doi.org/10.1093/aje/kwm040](https://www.zotero.org/google-docs/?SJrRhI)

[Mukherjee, S., Decina, P., Bocola, V., Saraceni, F., & Scapicchio, P. L. (1996). Diabetes mellitus in schizophrenic patients. *Compr Psychiatry*, *37*(1), 68–73.](https://www.zotero.org/google-docs/?SJrRhI)

[Naber, D. (1995). A self-rating to measure subjective effects of neuroleptic drugs, relationships to objective psychopathology, quality of life, compliance and other clinical variables. *Int Clin Psychopharmacol*, *10 Suppl 3*, 133–138.](https://www.zotero.org/google-docs/?SJrRhI)

[Narvaez, J. M., Twamley, E. W., McKibbin, C. L., Heaton, R. K., & Patterson, T. L. (2008). Subjective and objective quality of life in schizophrenia. *Schizophrenia Research*, *98*(1–3), 201–208. https://doi.org/10.1016/j.schres.2007.09.001](https://www.zotero.org/google-docs/?SJrRhI)

[Naslund, J. A., Whiteman, K. L., McHugo, G. J., Aschbrenner, K. A., Marsch, L. A., & Bartels, S. J. (2017). Lifestyle interventions for weight loss among overweight and obese adults with serious mental illness: A systematic review and meta-analysis. *Gen Hosp Psychiatry*, *47*, 83–102. https://doi.org/10.1016/j.genhosppsych.2017.04.003](https://www.zotero.org/google-docs/?SJrRhI)

[Nasrallah, H., Morosini, P., & Gagnon, D. D. (2008). Reliability, validity and ability to detect change of the Personal and Social Performance scale in patients with stable schizophrenia. *Psychiatry Res*, *161*(2), 213–224. https://doi.org/10.1016/j.psychres.2007.11.012](https://www.zotero.org/google-docs/?SJrRhI)

[Nazeri, A., Chakravarty, M. M., Rotenberg, D. J., Rajji, T. K., Rathi, Y., Michailovich, O. V., & Voineskos, A. N. (2015). Functional consequences of neurite orientation dispersion and density in humans across the adult lifespan. *The Journal of Neuroscience: The Official Journal of the Society for Neuroscience*, *35*(4), 1753–1762. https://doi.org/10.1523/JNEUROSCI.3979-14.2015](https://www.zotero.org/google-docs/?SJrRhI)

[Nazeri, A., Mulsant, B. H., Rajji, T. K., Levesque, M. L., Pipitone, J., Stefanik, L., Shahab, S., Roostaei, T., Wheeler, A. L., Chavez, S., & Voineskos, A. N. (2017). Gray Matter Neuritic Microstructure Deficits in Schizophrenia and Bipolar Disorder. *Biological Psychiatry*, *82*(10), 726–736. https://doi.org/10.1016/j.biopsych.2016.12.005](https://www.zotero.org/google-docs/?SJrRhI)

[Nedic Erjavec, G., Konjevod, M., Nikolac Perkovic, M., Svob Strac, D., Tudor, L., Barbas, C., Grune, T., Zarkovic, N., & Pivac, N. (2018). Short overview on metabolomic approach and redox changes in psychiatric disorders. *Redox Biology*, *14*, 178–186. https://doi.org/10.1016/j.redox.2017.09.002](https://www.zotero.org/google-docs/?SJrRhI)

[Nevarez-Flores, A. G., Sanderson, K., Breslin, M., Carr, V. J., Morgan, V. A., & Neil, A. L. (2019). Systematic review of global functioning and quality of life in people with psychotic disorders. *Epidemiol Psychiatr Sci*, *28*(1), 31–44. https://doi.org/10.1017/S2045796018000549](https://www.zotero.org/google-docs/?SJrRhI)

[NIDA. (2012). *The NIDA Quick Screen*. https://archives.drugabuse.gov/publications/resource-guide-screening-drug-use-in-general-medical-settings/nida-quick-screen](https://www.zotero.org/google-docs/?SJrRhI)

[Nielsen, R. E., Levander, S., Kjaersdam Telléus, G., Jensen, S. O. W., Østergaard Christensen, T., & Leucht, S. (2015). Second-generation antipsychotic effect on cognition in patients with schizophrenia-a meta-analysis of randomized clinical trials. *Acta Psychiatrica Scandinavica*, *131*(3). https://doi.org/10.1111/acps.12374](https://www.zotero.org/google-docs/?SJrRhI)

[Nierop, M. van, Bak, M., Graaf, R. de, Have, M. ten, Dorsselaer, S. van, & Winkel, R. van. (2016). The functional and clinical relevance of childhood trauma-related admixture of affective, anxious and psychosis symptoms. *Acta Psychiatrica Scandinavica*, *133*(2), 91–101. https://doi.org/10.1111/acps.12437](https://www.zotero.org/google-docs/?SJrRhI)

[Norman, R., Lecomte, T., Addington, D., & Anderson, E. (2017). Canadian Treatment Guidelines on Psychosocial Treatment of Schizophrenia in Adults. *Canadian Journal of Psychiatry Revue Canadienne de Psychiatrie*, *62*(9), 617–623. https://doi.org/10.1177/0706743717719894](https://www.zotero.org/google-docs/?SJrRhI)

[Norman, R. M. G., MacDougall, A., Manchanda, R., & Harricharan, R. (2018). An examination of components of recovery after five years of treatment in an early intervention program for psychosis. *Schizophrenia Research*, *195*, 469–474. https://doi.org/10.1016/j.schres.2017.08.054](https://www.zotero.org/google-docs/?SJrRhI)

[Norman, R. M. G., Malla, A. K., Cortese, L., Cheng, S., Diaz, K., McIntosh, E., McLean, T. S., Rickwood, A., & Voruganti, L. p. (1999). Symptoms and Cognition as Predictors of Community Functioning: A Prospective Analysis. *American Journal of Psychiatry*, *156*(3), 400–405. https://doi.org/10.1176/ajp.156.3.400](https://www.zotero.org/google-docs/?SJrRhI)

[Norman, R. M. G., Windell, D., Lynch, J., & Manchanda, R. (2013). Correlates of subjective recovery in an early intervention program for psychoses. *Early Intervention in Psychiatry*, *7*(3), 278–284. https://doi.org/10.1111/j.1751-7893.2012.00371.x](https://www.zotero.org/google-docs/?SJrRhI)

[Novick, D., Haro, J. M., Suarez, D., Vieta, E., & Naber, D. (2009). Recovery in the outpatient setting: 36-month results from the Schizophrenia Outpatients Health Outcomes (SOHO) study. *Schizophrenia Research*, *108*(1), 223–230. https://doi.org/10.1016/j.schres.2008.11.007](https://www.zotero.org/google-docs/?SJrRhI)

[Nucifora, F. C., Woznica, E., Lee, B. J., Cascella, N., & Sawa, A. (2019). Treatment resistant schizophrenia: Clinical, biological, and therapeutic perspectives. *Neurobiol Dis*, *131*, 104257. https://doi.org/10.1016/j.nbd.2018.08.016](https://www.zotero.org/google-docs/?SJrRhI)

[Nuechterlein, K. H., Subotnik, K. L., Turner, L. R., Ventura, J., Becker, D. R., & Drake, R. E. (2008). Individual placement and support for individuals with recent-onset schizophrenia: Integrating supported education and supported employment. *Psychiatric Rehabilitation Journal*, *31*(4), 340–349. https://doi.org/10.2975/31.4.2008.340.349](https://www.zotero.org/google-docs/?SJrRhI)

[Nuechterlein, K. H., Subotnik, K. L., Ventura, J., Turner, L. R., Gitlin, M. J., Gretchen-Doorly, D., Becker, D. R., Drake, R. E., Wallace, C. J., & Liberman, R. P. (2020). Enhancing return to work or school after a first episode of schizophrenia: The UCLA RCT of Individual Placement and Support and Workplace Fundamentals Module training. *Psychological Medicine*, *50*(1), 20–28. https://doi.org/10.1017/S0033291718003860](https://www.zotero.org/google-docs/?SJrRhI)

[Oakley, P., Kisely, S., Baxter, A., Harris, M., Desoe, J., Dziouba, A., & Siskind, D. (2018). *Increased mortality among people with schizophrenia and other non-affective psychotic disorders in the community: A systematic review and meta-analysis*.](https://www.zotero.org/google-docs/?SJrRhI)

Ojagbemi A., Emsely, R. & Gureje, O. (2017). Proposing the short Neurological Evaluation Scale. *Act Neuropsychiatrica, 10*, 236-243. https://doi.org/10.1017/neu.2016.55

[O’Donoghue, B., Roche, E., & Lane, A. (2016). Neighbourhood level social deprivation and the risk of psychotic disorders: A systematic review. *Social Psychiatry and Psychiatric Epidemiology*, *51*(7), 941–950. https://doi.org/10.1007/s00127-016-1233-4](https://www.zotero.org/google-docs/?SJrRhI)

[O’Donovan, M. C., Craddock, N., Norton, N., Williams, H., Peirce, T., Moskvina, V., Nikolov, I., Hamshere, M., Carroll, L., Georgieva, L., Dwyer, S., Holmans, P., Marchini, J. L., Spencer, C. C., Howie, B., Leung, H. T., Hartmann, A. M., Moller, H. J., Morris, D. W., … Cloninger, C. R. (2008). Identification of loci associated with schizophrenia by genome-wide association and follow-up. *Nat Genet*, *40*(9), 1053–1055. https://doi.org/10.1038/ng.201](https://www.zotero.org/google-docs/?SJrRhI)

[Oh, H., Yang, L. H., Anglin, D. M., & DeVylder, J. E. (2014). Perceived discrimination and psychotic experiences across multiple ethnic groups in the United States. *Schizophrenia Research*, *157*(1), 259–265. https://doi.org/10.1016/j.schres.2014.04.036](https://www.zotero.org/google-docs/?SJrRhI)

[Olfson, M., Gerhard, T., Huang, C., Crystal, S., & Stroup, T. S. (2015). Premature Mortality Among Adults With Schizophrenia in the United States. *JAMA Psychiatry*, *72*(12), 1172–1181. https://doi.org/10.1001/jamapsychiatry.2015.1737](https://www.zotero.org/google-docs/?SJrRhI)

[Oliveira-Maia, A. J., Mendonça, C., Pessoa, M. J., Camacho, M., & Gago, J. (2016). The Mental Health Recovery Measure Can Be Used to Assess Aspects of Both Customer-Based and Service-Based Recovery in the Context of Severe Mental Illness. *Frontiers in Psychology*, *7*. https://doi.org/10.3389/fpsyg.2016.01679](https://www.zotero.org/google-docs/?SJrRhI)

[Oliver, L. D., Haltigan, J. D., Gold, J. M., Foussias, G., DeRosse, P., Buchanan, R. W., Malhotra, A. K., Voineskos, A. N., & Spins Group. (2019). Lower- and Higher-Level Social Cognitive Factors Across Individuals With Schizophrenia Spectrum Disorders and Healthy Controls: Relationship With Neurocognition and Functional Outcome. *Schizophr Bull*, *45*(3), 629–638. https://doi.org/10.1093/schbul/sby114](https://www.zotero.org/google-docs/?SJrRhI)

[Ontario Hospital Association. (2020). *The Importance of Co-Design in Realizing the Potential of Integrated Care*. https://www.oha.com/discovery/past-issues/2019/integrated-care/in-this-issue/the-importance-of-co-design-in-realizing-the-potential-of-integrated-care](https://www.zotero.org/google-docs/?SJrRhI)

[Ospina-Pinillos, L., Davenport, T., Diaz, A. M., Navarro-Mancilla, A., Scott, E. M., & Hickie, I. B. (2019). Using Participatory Design Methodologies to Co-Design and Culturally Adapt the Spanish Version of the Mental Health eClinic: Qualitative Study. *Journal of Medical Internet Research*, *21*(8), e14127. https://doi.org/10.2196/14127](https://www.zotero.org/google-docs/?SJrRhI)

[Palm, U., Keeser, D., Hasan, A., Kupka, M. J., Blautzik, J., Sarubin, N., Kaymakanova, F., Unger, I., Falkai, P., Meindl, T., Ertl-Wagner, B., & Padberg, F. (2016). Prefrontal Transcranial Direct Current Stimulation for Treatment of Schizophrenia With Predominant Negative Symptoms: A Double-Blind, Sham-Controlled Proof-of-Concept Study. *Schizophrenia Bulletin*, *42*(5), 1253–1261. https://doi.org/10.1093/schbul/sbw041](https://www.zotero.org/google-docs/?SJrRhI)

[Palmer, V., Chondros, P., Piper, D., Callander, R., Weavell, W., Godbee, K., Potiriadis, M., Richard, L., Densely, K., Herrman, H., Furler, J., Pierce, D., Schuster, T., Iedema, R., & Gunn, J. (2015). The CORE study protocol: A stepped wedge cluster randomised controlled trial to test a co-design technique to optimise psychosocial recovery outcomes for people affected by mental illness in the community mental health setting. *BMJ Open*, *5*(3), e006688. https://doi.org/10.1136/bmjopen-2014-006688](https://www.zotero.org/google-docs/?SJrRhI)

[Palmer, V., Gunn, J., Godbee, K., Potiriadis, M., Densley, K., Chondros, P., Herrman, H., Callander, R., Weavell, W., Furler, J., Piper, D., Pierce, D., Iedema, R., & Richard, L. (2015). *Getting to the CORE of the links between engagement, experience and recovery outcomes*. https://rune.une.edu.au/web/handle/1959.11/17332](https://www.zotero.org/google-docs/?SJrRhI)

[Palmer, V., Piper, D., Richard, L., Furler, J., Herrman, H., Cameron, J., Godbee, K., Pierce, D., Callander, R., Weavell, W., Gunn, J., & Iedema, R. (2016). Balancing Opposing Forces—A Nested Process Evaluation Study Protocol for a Stepped Wedge Designed Cluster Randomized Controlled Trial of an Experience Based Codesign Intervention: The CORE Study. *International Journal of Qualitative Methods*, *15*(1), 1609406916672216. https://doi.org/10.1177/1609406916672216](https://www.zotero.org/google-docs/?SJrRhI)

[Patel, R., Jayatilleke, N., Broadbent, M., Chang, C. K., Foskett, N., Gorrell, G., Hayes, R. D., Jackson, R., Johnston, C., Shetty, H., Roberts, A., McGuire, P., & Stewart, R. (2015). Negative symptoms in schizophrenia: A study in a large clinical sample of patients using a novel automated method. *BMJ Open*, *5*(9), e007619. https://doi.org/10.1136/bmjopen-2015-007619](https://www.zotero.org/google-docs/?SJrRhI)

[Patel, R., Lloyd, T., Jackson, R., Ball, M., Shetty, H., Broadbent, M., Geddes, J. R., Stewart, R., McGuire, P., & Taylor, M. (2015). Mood instability is a common feature of mental health disorders and is associated with poor clinical outcomes. *BMJ Open*, *5*(5), e007504. https://doi.org/10.1136/bmjopen-2014-007504](https://www.zotero.org/google-docs/?SJrRhI)

[Pazoki, R., Lin, B. D., van Eijk, K. R., Schijven, D., de Zwarte, S., Guloksuz, S., & Luykx, J. J. (2020). Phenome-wide and genome-wide analyses of quality of life in schizophrenia. *BJPsych Open*, *7*(1), e13. https://doi.org/10.1192/bjo.2020.140](https://www.zotero.org/google-docs/?SJrRhI)

[Pearlman, L. A. (1997). Trauma and the Self. *Journal of Emotional Abuse*, *1*(1), 7–25. https://doi.org/10.1300/J135v01n01_02](https://www.zotero.org/google-docs/?SJrRhI)

[Perala, J., Suvisaari, J., Saarni, S. I., Kuoppasalmi, K., Isometsa, E., Pirkola, S., Partonen, T., Tuulio-Henriksson, A., Hintikka, J., Kieseppa, T., Harkanen, T., Koskinen, S., & Lonnqvist, J. (2007). Lifetime prevalence of psychotic and bipolar I disorders in a general population. *Arch Gen Psychiatry*, *64*(1), 19–28. https://doi.org/10.1001/archpsyc.64.1.19](https://www.zotero.org/google-docs/?SJrRhI)

[Petrides, G., Malur, C., Braga, R. J., Bailine, S. H., Schooler, N. R., Malhotra, A. K., Kane, J. M., Sanghani, S., Goldberg, T. E., John, M., & Mendelowitz, A. (2015). Electroconvulsive therapy augmentation in clozapine-resistant schizophrenia: A prospective, randomized study. *The American Journal of Psychiatry*, *172*(1), 52–58. https://doi.org/10.1176/appi.ajp.2014.13060787](https://www.zotero.org/google-docs/?SJrRhI)

[Pogue-Geile, M. F., & Harrow, M. (1984). Negative and Positive Symptoms in Schizophrenia and Depression: A Followup. *Schizophrenia Bulletin*, *10*(3), 371–387. https://doi.org/10.1093/schbul/10.3.371](https://www.zotero.org/google-docs/?SJrRhI)

[Pogue-Geile, M. F., & Harrow, M. (1985). Negative Symptoms in Schizophrenia: Their Longitudinal Course and Prognostic Importance. *Schizophrenia Bulletin*, *11*(3), 427–439. https://doi.org/10.1093/schbul/11.3.427](https://www.zotero.org/google-docs/?SJrRhI)

[Porter, D. W., Kerr, B. D., Flatt, P. R., Holscher, C., & Gault, V. A. (2010). Four weeks administration of Liraglutide improves memory and learning as well as glycaemic control in mice with high fat dietary-induced obesity and insulin resistance. *Diabetes Obes Metab*, *12*(10), 891–899. https://doi.org/10.1111/j.1463-1326.2010.01259.x](https://www.zotero.org/google-docs/?SJrRhI)

[Posner, K., Brown, G. K., Stanley, B., Brent, D. A., Yershova, K. V., Oquendo, M. A., Currier, G. W., Melvin, G. A., Greenhill, L., Shen, S., & Mann, J. J. (2011). The Columbia-Suicide Severity Rating Scale: Initial validity and internal consistency findings from three multisite studies with adolescents and adults. *Am J Psychiatry*, *168*(12), 1266–1277. https://doi.org/10.1176/appi.ajp.2011.10111704](https://www.zotero.org/google-docs/?SJrRhI)

[Prasad, K. M. R., Sahni, S. D., Rohm, B. R., & Keshavan, M. S. (2005). Dorsolateral prefrontal cortex morphology and short-term outcome in first-episode schizophrenia. *Psychiatry Research*, *140*(2), 147–155. https://doi.org/10.1016/j.pscychresns.2004.05.009](https://www.zotero.org/google-docs/?SJrRhI)

[Qiu, C., & Fratiglioni, L. (2015). *A major role for cardiovascular burden in age-related cognitive decline*.](https://www.zotero.org/google-docs/?SJrRhI)

[Rabinowitz, J., Levine, S. Z., Garibaldi, G., Bugarski-Kirola, D., Berardo, C. G., & Kapur, S. (2012). Negative symptoms have greater impact on functioning than positive symptoms in schizophrenia: Analysis of CATIE data. *Schizophrenia Research*, *137*(1), 147–150. https://doi.org/10.1016/j.schres.2012.01.015](https://www.zotero.org/google-docs/?SJrRhI)

[Rajkumar, A. P., Horsdal, H. T., Wimberley, T., Cohen, D., Mors, O., Borglum, A. D., & Gasse, C. (2017). Endogenous and Antipsychotic-Related Risks for Diabetes Mellitus in Young People With Schizophrenia: A Danish Population-Based Cohort Study. *Am J Psychiatry*, *174*(7), 686–694. https://doi.org/10.1176/appi.ajp.2016.16040442](https://www.zotero.org/google-docs/?SJrRhI)

[Reed, R. A., Harrow, M., Herbener, E. S., & Martin, E. M. (2002). Executive function in schizophrenia: Is it linked to psychosis and poor life functioning? *The Journal of Nervous and Mental Disease*, *190*(11), 725–732. https://doi.org/10.1097/00005053-200211000-00001](https://www.zotero.org/google-docs/?SJrRhI)

[Reininghaus, U. A., Morgan, C., Simpson, J., Dazzan, P., Morgan, K., Doody, G. A., Bhugra, D., Leff, J., Jones, P., Murray, R., Fearon, P., & Craig, T. K. J. (2008). Unemployment, social isolation, achievement-expectation mismatch and psychosis: Findings from the AESOP Study. *Social Psychiatry and Psychiatric Epidemiology*, *43*(9), 743–751. https://doi.org/10.1007/s00127-008-0359-4](https://www.zotero.org/google-docs/?SJrRhI)

[Remington, G., KWON, J., COLLINS, A., LAPORTE, D., Mann, S., & Christensen, B. (2007). The use of electronic monitoring (MEMS®) to evaluate antipsychotic compliance in outpatients with schizophrenia. *Schizophrenia Research*, *90*(1–3), 229–237. https://doi.org/10.1016/j.schres.2006.11.015](https://www.zotero.org/google-docs/?SJrRhI)

[Remington, Gary, Addington, D., Honer, W., Ismail, Z., Raedler, T., & Teehan, M. (2017). Guidelines for the Pharmacotherapy of Schizophrenia in Adults. *Canadian Journal of Psychiatry. Revue Canadienne De Psychiatrie*, *62*(9), 604–616. https://doi.org/10.1177/0706743717720448](https://www.zotero.org/google-docs/?SJrRhI)

[Resnick, S. G., Rosenheck, R. A., & Lehman, A. F. (2004). An exploratory analysis of correlates of recovery. *Psychiatr Serv*, *55*(5), 540–547. https://doi.org/10.1176/appi.ps.55.5.540](https://www.zotero.org/google-docs/?SJrRhI)

[Revell, E. R., Neill, J. C., Harte, M., Khan, Z., & Drake, R. J. (2015). A systematic review and meta-analysis of cognitive remediation in early schizophrenia. *Schizophrenia Research*, *168*(1–2). https://doi.org/10.1016/j.schres.2015.08.017](https://www.zotero.org/google-docs/?SJrRhI)

[Ripke, S., Walters, J. T., & O’Donovan, M. C. (2020). Mapping genomic loci prioritises genes and implicates synaptic biology in schizophrenia. *MedRxiv*, 2020.09.12.20192922. https://doi.org/10.1101/2020.09.12.20192922](https://www.zotero.org/google-docs/?SJrRhI)

[Ritsner, M., Kurs, R., Gibel, A., Ratner, Y., & Endicott, J. (2005). Validity of an abbreviated quality of life enjoyment and satisfaction questionnaire (Q-LES-Q-18) for schizophrenia, schizoaffective, and mood disorder patients. *Quality of Life Research*, *14*(7), 1693–1703.](https://www.zotero.org/google-docs/?SJrRhI)

[Ritsner, M. S., Arbitman, M., Lisker, A., & Ponizovsky, A. M. (2012). Ten-year quality of life outcomes among patients with schizophrenia and schizoaffective disorder II. Predictive value of psychosocial factors. *Quality of Life Research : An International Journal of Quality of Life Aspects of Treatment, Care and Rehabilitation*, *21*(6), 1075–1084. https://doi.org/10.1007/s11136-011-0015-4](https://www.zotero.org/google-docs/?SJrRhI)

[Robert, G., Cornwell, J., Locock, L., Purushotham, A., Sturmey, G., & Gager, M. (2015). Patients and staff as codesigners of healthcare services. *BMJ*, *350*, g7714. https://doi.org/10.1136/bmj.g7714](https://www.zotero.org/google-docs/?SJrRhI)

[Robinson, D. G., Woerner, M. G., McMeniman, M., Mendelowitz, A., & Bilder, R. M. (2004). Symptomatic and functional recovery from a first episode of schizophrenia or schizoaffective disorder. *The American Journal of Psychiatry*, *161*(3), 473–479.](https://www.zotero.org/google-docs/?SJrRhI)

[Roe, D., Mashiach-Eizenberg, M., & Lysaker, P. H. (2011). The relation between objective and subjective domains of recovery among persons with schizophrenia-related disorders. *Schizophrenia Research*, *131*(1–3), 133–138. https://doi.org/10.1016/j.schres.2011.05.023](https://www.zotero.org/google-docs/?SJrRhI)

[Ronan, L., Alexander-Bloch, A. F., Wagstyl, K., Farooqi, S., Brayne, C., Tyler, L. K., Cam-CAN, & Fletcher, P. C. (2016). Obesity associated with increased brain age from midlife. *Neurobiology of Aging*, *47*, 63–70. https://doi.org/10.1016/j.neurobiolaging.2016.07.010](https://www.zotero.org/google-docs/?SJrRhI)

[Roncone, R., Falloon, I. R. H., Mazza, M., De Risio, A., Pollice, R., Necozione, S., Morosini, P., & Casacchia, M. (2002). Is theory of mind in schizophrenia more strongly associated with clinical and social functioning than with neurocognitive deficits? *Psychopathology*, *35*(5), 280–288.](https://www.zotero.org/google-docs/?SJrRhI)

[Rosen, C., Grossman, L. S., Harrow, M., Bonner-Jackson, A., & Faull, R. (2011). Diagnostic and prognostic significance of Schneiderian first-rank symptoms: A 20-year longitudinal study of schizophrenia and bipolar disorder. *Comprehensive Psychiatry*, *52*(2), 126–131. https://doi.org/10.1016/j.comppsych.2010.06.005](https://www.zotero.org/google-docs/?SJrRhI)

Rosenbaum, S., Morell, R., Abdel-Baki, A., Ahmadpanah, M., Anilkumar, T.V., Baie, L., Bauman, A., Bender, S.,Boyan Han, J., Brand, S., Bratland-Sanda, S., Bueno-Antequera, J., Camaz Deslandes, A., Carneiro., L., Carraro, A., Castañeda, C.P., Castro Monteiro, F., Chapman, J, Cahu, J.Y.,…Ward, P.B. (2020). Assessing physical activity in people with mental illness: 23-country reliability and validity of the simple physical activity questionnaire (SIMPAQ). *BMC Psychiatry 20* (108), 1-12. https://doi.org/10.1186/s12888-020-2473-0

[Rosenheck, R., Kasprow, W., Frisman, L., & Liu-Mares, W. (2003). Cost-effectiveness of supported housing for homeless persons with mental illness. *Archives of General Psychiatry*, *60*(9), 940–951. https://doi.org/10.1001/archpsyc.60.9.940](https://www.zotero.org/google-docs/?SJrRhI)

[Rosenheck, R., Leslie, D., Keefe, R., McEvoy, J., Swartz, M., Perkins, D., Stroup, S., Hsiao, J. K., & Lieberman, J. (2006). Barriers to Employment for People With Schizophrenia. *The American Journal of Psychiatry*, *163*(3), 411–417. https://doi.org/10.1176/appi.ajp.163.3.411](https://www.zotero.org/google-docs/?SJrRhI)

[Rosenheck, R., Mueser, K. T., Sint, K., Lin, H., Lynde, D. W., Glynn, S. M., Robinson, D. G., Schooler, N. R., Marcy, P., Mohamed, S., & Kane, J. M. (2017). Supported employment and education in comprehensive, integrated care for first episode psychosis: Effects on work, school, and disability income. *Schizophrenia Research*, *182*, 120–128. https://doi.org/10.1016/j.schres.2016.09.024](https://www.zotero.org/google-docs/?SJrRhI)

[Rotenberg, M. (2019). Rehabilitation and recovery for ethnic minority patients with severe mental illness. *BJPsych Advances*, *25*(4), 223–228. https://doi.org/10.1192/bja.2019.13](https://www.zotero.org/google-docs/?SJrRhI)

[Rotenberg, M., Anderson, K. K., & McKenzie, K. (2020). Social capital and psychosis: A scoping review. *Social Psychiatry and Psychiatric Epidemiology*, *55*(6), 659–671. https://doi.org/10.1007/s00127-019-01812-9](https://www.zotero.org/google-docs/?SJrRhI)

[Rotenberg, M., & Rudnick, A. (2017). Recent developments in person-centered psychiatry: Present and future psychiatric rehabilitation. *European Journal for Person Centered Healthcare*, *5*(2), 256–262.](https://www.zotero.org/google-docs/?SJrRhI)

[Ruzickova, M., Slaney, C., Garnham, J., & Alda, M. (2003). *Clinical Features of Bipolar Disorder With and Without Comorbid Diabetes Mellitus*.](https://www.zotero.org/google-docs/?SJrRhI)

[Ryder, A. G., Alden, L. E., & Paulhus, D. L. (2000). Is acculturation unidimensional or bidimensional? A head-to-head comparison in the prediction of personality, self-identity, and adjustment. *Journal of Personality and Social Psychology*, *79*(1), 49–65. https://doi.org/10.1037//0022-3514.79.1.49](https://www.zotero.org/google-docs/?SJrRhI)

[Sabe, M., Kirschner, M., & Kaiser, S. (2019). Prodopaminergic Drugs for Treating the Negative Symptoms of Schizophrenia: Systematic Review and Meta-analysis of Randomized Controlled Trials. *Journal of Clinical Psychopharmacology*, *39*(6), 658–664. https://doi.org/10.1097/JCP.0000000000001124](https://www.zotero.org/google-docs/?SJrRhI)

[Saha, S., Chant, D., & McGrath, J. (2007). A systematic review of mortality in schizophrenia: Is the differential mortality gap worsening over time? *Arch Gen Psychiatry*, *64*(10), 1123–1131. https://doi.org/64/10/1123 [pii] 10.1001/archpsyc.64.10.1123](https://www.zotero.org/google-docs/?SJrRhI)

[Sajatovic, M., Velligan, D. I., Weiden, P. J., Valenstein, M. A., & Ogedegbe, G. (2010). Measurement of psychiatric treatment adherence. *J Psychosom Res*, *69*(6), 591–599. https://doi.org/10.1016/j.jpsychores.2009.05.007](https://www.zotero.org/google-docs/?SJrRhI)

[Salomon, J. A., Haagsma, J. A., Davis, A., de Noordhout, C. M., Polinder, S., Havelaar, A. H., Cassini, A., Devleesschauwer, B., Kretzschmar, M., Speybroeck, N., Murray, C. J., & Vos, T. (2015). Disability weights for the Global Burden of Disease 2013 study. *Lancet Glob Health*, *3*(11), e712-23. https://doi.org/10.1016/S2214-109X(15)00069-8](https://www.zotero.org/google-docs/?SJrRhI)

[Salzer, M. S., & Brusilovskiy, E. (2014). Advancing Recovery Science: Reliability and Validity Properties of the Recovery Assessment Scale. *Psychiatric Services*, *65*(4), 442–453. https://doi.org/10.1176/appi.ps.201300089](https://www.zotero.org/google-docs/?SJrRhI)

[Samara, M. T., Dold, M., Gianatsi, M., Nikolakopoulou, A., Helfer, B., Salanti, G., & Leucht, S. (2016). Efficacy, Acceptability, and Tolerability of Antipsychotics in Treatment-Resistant Schizophrenia: A Network Meta-analysis. *JAMA Psychiatry*, *73*(3), 199–210. https://doi.org/10.1001/jamapsychiatry.2015.2955](https://www.zotero.org/google-docs/?SJrRhI)

[Sanghani, S. N., Petrides, G., & Kellner, C. H. (2018). Electroconvulsive therapy (ECT) in schizophrenia: A review of recent literature. *Current Opinion in Psychiatry*, *31*(3), 213–222. https://doi.org/10.1097/YCO.0000000000000418](https://www.zotero.org/google-docs/?SJrRhI)

[Santesteban-Echarri, O., Piskulic, D., Nyman, R. K., & Addington, J. (2020). Telehealth interventions for schizophrenia-spectrum disorders and clinical high-risk for psychosis individuals: A scoping review. *J Telemed Telecare*, *26*(1–2), 14–20. https://doi.org/10.1177/1357633X18794100](https://www.zotero.org/google-docs/?SJrRhI)

[Santoro, M. L., Ota, V., de Jong, S., Noto, C., Spindola, L. M., Talarico, F., Gouvea, E., Lee, S. H., Moretti, P., Curtis, C., Patel, H., Newhouse, S., Carvalho, C. M., Gadelha, A., Cordeiro, Q., Bressan, R. A., Belangero, S. I., & Breen, G. (2018). Polygenic risk score analyses of symptoms and treatment response in an antipsychotic-naive first episode of psychosis cohort. *Transl Psychiatry*, *8*(1), 174. https://doi.org/10.1038/s41398-018-0230-7](https://www.zotero.org/google-docs/?SJrRhI)

[Saperia, S., Da Silva, S., Siddiqui, I., McDonald, K., Agid, O., Remington, G., & Foussias, G. (2018). Investigating the predictors of happiness, life satisfaction and success in schizophrenia. *Compr Psychiatry*, *81*, 42–47. https://doi.org/10.1016/j.comppsych.2017.11.005](https://www.zotero.org/google-docs/?SJrRhI)

[Saxena, S., & Maj, M. (2017). *Physical health of people with severe mental disorders: Leave no one behind*.](https://www.zotero.org/google-docs/?SJrRhI)

[Schäfer, I., & Fisher, H. L. (2011). Childhood trauma and psychosis—What is the evidence? *Dialogues in Clinical Neuroscience*, *13*(3), 360–365.](https://www.zotero.org/google-docs/?SJrRhI)

[Schafer, J. (1997). *Analysis of Incomplete Multivariate Data*. Chapman and Hall/CRC. https://doi.org/10.1201/9780367803025](https://www.zotero.org/google-docs/?SJrRhI)

[Schizophrenia Working Group of the Psychiatric Genomics Consortium. (2014). Biological insights from 108 schizophrenia-associated genetic loci. *Nature*, *511*(7510), 421–427. https://doi.org/10.1038/nature13595](https://www.zotero.org/google-docs/?SJrRhI)

[Schmidt, S. J., Mueller, D. R., & Roder, V. (2011). Social cognition as a mediator variable between neurocognition and functional outcome in schizophrenia: Empirical review and new results by structural equation modeling. *Schizophrenia Bulletin*, *37 Suppl 2*, S41-54. https://doi.org/10.1093/schbul/sbr079](https://www.zotero.org/google-docs/?SJrRhI)

[Schofield, P., Thygesen, M., Das-Munshi, J., Becares, L., Cantor-Graae, E., Pedersen, C., & Agerbo, E. (2017). Ethnic density, urbanicity and psychosis risk for migrant groups – A population cohort study. *Schizophrenia Research*, *190*, 82–87. https://doi.org/10.1016/j.schres.2017.03.032](https://www.zotero.org/google-docs/?SJrRhI)

[Schooler, N. R. (2006). Relapse prevention and recovery in the treatment of schizophrenia. *The Journal of Clinical Psychiatry*, *67 Suppl 5*, 19–23.](https://www.zotero.org/google-docs/?SJrRhI)

[Seidell, J. C., Bakker, C. J., & van der Kooy, K. (1990). Imaging techniques for measuring adipose-tissue distribution—A comparison between computed tomography and 1.5-T magnetic resonance. *The American Journal of Clinical Nutrition*, *51*(6), 953–957. https://doi.org/10.1093/ajcn/51.6.953](https://www.zotero.org/google-docs/?SJrRhI)

[Sekar, A., Bialas, A. R., de Rivera, H., Davis, A., Hammond, T. R., Kamitaki, N., Tooley, K., Presumey, J., Baum, M., Van Doren, V., Genovese, G., Rose, S. A., Handsaker, R. E., Daly, M. J., Carroll, M. C., Stevens, B., & McCarroll, S. A. (2016). Schizophrenia risk from complex variation of complement component 4. *Nature*, *530*(7589), 177–183. https://doi.org/10.1038/nature16549](https://www.zotero.org/google-docs/?SJrRhI)

[Selten, J.-P., Ven, E. van der, & Termorshuizen, F. (2020). Migration and psychosis: A meta-analysis of incidence studies. *Psychological Medicine*, *50*(2), 303–313. https://doi.org/10.1017/S0033291719000035](https://www.zotero.org/google-docs/?SJrRhI)

[Sergi, M. J., Rassovsky, Y., Nuechterlein, K. H., & Green, M. F. (2006). Social perception as a mediator of the influence of early visual processing on functional status in schizophrenia. *The American Journal of Psychiatry*, *163*(3), 448–454. https://doi.org/10.1176/appi.ajp.163.3.448](https://www.zotero.org/google-docs/?SJrRhI)

[Sethi, S., & Brietzke, E. (2015). Omics-Based Biomarkers: Application of Metabolomics in Neuropsychiatric Disorders. *International Journal of Neuropsychopharmacology*, *19*(3). https://doi.org/10.1093/ijnp/pyv096](https://www.zotero.org/google-docs/?SJrRhI)

[Seymour, C. W., Kennedy, J. N., Wang, S., Chang, C. H., Elliott, C. F., Xu, Z., Berry, S., Clermont, G., Cooper, G., Gomez, H., Huang, D. T., Kellum, J. A., Mi, Q., Opal, S. M., Talisa, V., van der Poll, T., Visweswaran, S., Vodovotz, Y., Weiss, J. C., … Angus, D. C. (2019). Derivation, Validation, and Potential Treatment Implications of Novel Clinical Phenotypes for Sepsis. *JAMA*, *321*(20), 2003–2017. https://doi.org/10.1001/jama.2019.5791](https://www.zotero.org/google-docs/?SJrRhI)

[Shamsi, S., Lau, A., Lencz, T., Burdick, K. E., DeRosse, P., Brenner, R., Lindenmayer, J.-P., & Malhotra, A. K. (2011). Cognitive and symptomatic predictors of functional disability in schizophrenia. *Schizophrenia Research*, *126*(1–3), 257–264. https://doi.org/10.1016/j.schres.2010.08.007](https://www.zotero.org/google-docs/?SJrRhI)

[Shi, C., Yu, X., Cheung, E. F. C., Shum, D. H. K., & Chan, R. C. K. (2014). Revisiting the therapeutic effect of rTMS on negative symptoms in schizophrenia: A meta-analysis. *Psychiatry Research*, *215*(3), 505–513. https://doi.org/10.1016/j.psychres.2013.12.019](https://www.zotero.org/google-docs/?SJrRhI)

[Shifman, S., Bronstein, M., Sternfeld, M., Pisante-Shalom, A., Lev-Lehman, E., Weizman, A., Reznik, I., Spivak, B., Grisaru, N., Karp, L., Schiffer, R., Kotler, M., Strous, R. D., Swartz-Vanetik, M., Knobler, H. Y., Shinar, E., Beckmann, J. S., Yakir, B., Risch, N., … Darvasi, A. (2002). A highly significant association between a COMT haplotype and schizophrenia. *Am J Hum Genet*, *71*(6), 1296–1302. https://doi.org/10.1086/344514](https://www.zotero.org/google-docs/?SJrRhI)

[Shuster, A., Patlas, M., Pinthus, J. H., & Mourtzakis, M. (2012). The clinical importance of visceral adiposity: A critical review of methods for visceral adipose tissue analysis. *The British Journal of Radiology*, *85*(1009), 1–10. https://doi.org/10.1259/bjr/38447238](https://www.zotero.org/google-docs/?SJrRhI)

[Sicras-Mainar, A., Maurino, J., Ruiz-Beato, E., & Navarro-Artieda, R. (2014). Prevalence of metabolic syndrome according to the presence of negative symptoms in patients with schizophrenia. *Neuropsychiatric Disease and Treatment*, *11*. https://doi.org/10.2147/NDT.S75449](https://www.zotero.org/google-docs/?SJrRhI)

[Simpson, G. M., & Angus, J. W. (1970). A rating scale for extrapyramidal side effects. *Acta Psychiatrica Scandinavica*, *212*, 11–19.](https://www.zotero.org/google-docs/?SJrRhI)

[Sinclair, D. J., Zhao, S., Qi, F., Nyakyoma, K., Kwong, J. S., & Adams, C. E. (2019). Electroconvulsive therapy for treatment-resistant schizophrenia. *The Cochrane Database of Systematic Reviews*, *3*, CD011847. https://doi.org/10.1002/14651858.CD011847.pub2](https://www.zotero.org/google-docs/?SJrRhI)

[Sirey, J. A., Meyers, B. S., Teresi, J. A., Bruce, M. L., Ramirez, M., Raue, P. J., Perlick, D. A., & Holmes, D. (2005). The Cornell Service Index as a measure of health service use. *Psychiatric Services (Washington, D.C.)*, *56*(12), 1564–1569. https://doi.org/10.1176/appi.ps.56.12.1564](https://www.zotero.org/google-docs/?SJrRhI)

[Siskind, D. J., Hahn, M., Correll, C. U., Fink-Jensen, A., Russell, A. W., Bak, N., Broberg, B. V., Larsen, J., Ishoy, P. L., Vilsboll, T., Knop, F. K., Kisely, S., & Ebdrup, B. H. (2019). Glucagon-like peptide-1 receptor agonists for antipsychotic-associated cardio-metabolic risk factors: A systematic review and individual participant data meta-analysis. *Diabetes Obes Metab*, *21*(2), 293–302. https://doi.org/10.1111/dom.13522](https://www.zotero.org/google-docs/?SJrRhI)

[Siskind, D. J., Lee, M., Ravindran, A., Zhang, Q., Ma, E., Motamarri, B., & Kisely, S. (2018). Augmentation strategies for clozapine refractory schizophrenia: A systematic review and meta-analysis. *The Australian and New Zealand Journal of Psychiatry*, *52*(8), 751–767. https://doi.org/10.1177/0004867418772351](https://www.zotero.org/google-docs/?SJrRhI)

[Siskind, D. J., Siskind, V., & Kisely, S. (2017). Clozapine Response Rates among People with Treatment-Resistant Schizophrenia: Data from a Systematic Review and Meta-Analysis. *Canadian Journal of Psychiatry. Revue Canadienne De Psychiatrie*, *62*(11), 772–777. https://doi.org/10.1177/0706743717718167](https://www.zotero.org/google-docs/?SJrRhI)

[Skevington, S. M., Lotfy, M., & O’Connell, K. A. (2004). The World Health Organization’s WHOQOL-BREF quality of life assessment: Psychometric properties and results of the international field trial. A Report from the WHOQOL Group. *Quality of Life Research*, *13*(2), 299–310. https://doi.org/10.1023/B:QURE.0000018486.91360.00](https://www.zotero.org/google-docs/?SJrRhI)

[Slade, M., Amering, M., & Oades, L. (2008). Recovery: An international perspective. *Epidemiologia E Psichiatria Sociale*, *17*(2), 128–137. https://doi.org/10.1017/s1121189x00002827](https://www.zotero.org/google-docs/?SJrRhI)

[Smith, R. C., Boules, S., Mattiuz, S., Youssef, M., Tobe, R. H., Sershen, H., Lajtha, A., Nolan, K., Amiaz, R., & Davis, J. M. (2015). Effects of transcranial direct current stimulation (tDCS) on cognition, symptoms, and smoking in schizophrenia: A randomized controlled study. *Schizophrenia Research*, *168*(1–2), 260–266. https://doi.org/10.1016/j.schres.2015.06.011](https://www.zotero.org/google-docs/?SJrRhI)

[Sommer, I. E., Begemann, M. J. H., Temmerman, A., & Leucht, S. (2012). Pharmacological augmentation strategies for schizophrenia patients with insufficient response to clozapine: A quantitative literature review. *Schizophrenia Bulletin*, *38*(5), 1003–1011. https://doi.org/10.1093/schbul/sbr004](https://www.zotero.org/google-docs/?SJrRhI)

[Soontornniyomkij, V., Lee, E. E., Jin, H., Martin, A. S., Daly, R. E., Liu, J., Tu, X. M., Eyler, L. T., & Jeste, D. V. (2019). Clinical correlates of insulin resistance in chronic schizophrenia: Relationship to negative symptoms. *Frontiers in Psychiatry*, *10*(APR). https://doi.org/10.3389/fpsyt.2019.00251](https://www.zotero.org/google-docs/?SJrRhI)

[Soundy, A., Stubbs, B., Roskell, C., Williams, S. E., Fox, A., & Vancampfort, D. (2015). Identifying the facilitators and processes which influence recovery in individuals with schizophrenia: A systematic review and thematic synthesis. *Journal of Mental Health*, *24*(2), 103–110. https://doi.org/10.3109/09638237.2014.998811](https://www.zotero.org/google-docs/?SJrRhI)

[Spangaro, M., Mazza, E., Poletti, S., Cavallaro, R., & Benedetti, F. (2018). Obesity influences white matter integrity in schizophrenia. *Psychoneuroendocrinology*, *97*. https://doi.org/10.1016/j.psyneuen.2018.07.017](https://www.zotero.org/google-docs/?SJrRhI)

[Springham, N., & Robert, G. (2015). Experience based co-design reduces formal complaints on an acute mental health ward. *BMJ Open Quality*, *4*(1), u209153.w3970. https://doi.org/10.1136/bmjquality.u209153.w3970](https://www.zotero.org/google-docs/?SJrRhI)

[Staal, W. G., Hulshoff Pol, H. E., & Kahn, R. S. (1999). Outcome of schizophrenia in relation to brain abnormalities. *Schizophrenia Bulletin*, *25*(2), 337–348. https://doi.org/10.1093/oxfordjournals.schbul.a033382](https://www.zotero.org/google-docs/?SJrRhI)

[Steen, R. G., Mull, C., McClure, R., Hamer, R. M., & Lieberman, J. A. (2006). Brain volume in first-episode schizophrenia: Systematic review and meta-analysis of magnetic resonance imaging studies. *Br J Psychiatry*, *188*, 510–518. https://doi.org/10.1192/bjp.188.6.510](https://www.zotero.org/google-docs/?SJrRhI)

[Stefansson, H., Ophoff, R. A., Steinberg, S., Andreassen, O. A., Cichon, S., Rujescu, D., Werge, T., Pietilainen, O. P., Mors, O., Mortensen, P. B., Sigurdsson, E., Gustafsson, O., Nyegaard, M., Tuulio-Henriksson, A., Ingason, A., Hansen, T., Suvisaari, J., Lonnqvist, J., Paunio, T., … Collier, D. A. (2009). Common variants conferring risk of schizophrenia. *Nature*, *460*(7256), 744–747. https://doi.org/10.1038/nature08186](https://www.zotero.org/google-docs/?SJrRhI)

[Stergiopoulos, V., Gozdzik, A., Misir, V., Skosireva, A., Connelly, J., Sarang, A., Whisler, A., Hwang, S. W., O’Campo, P., & McKenzie, K. (2015). Effectiveness of Housing First with Intensive Case Management in an Ethnically Diverse Sample of Homeless Adults with Mental Illness: A Randomized Controlled Trial. *PLOS ONE*, *10*(7), e0130281. https://doi.org/10.1371/journal.pone.0130281](https://www.zotero.org/google-docs/?SJrRhI)

[Stover, P. J., Harlan, W. R., Hammond, J. A., Hendershot, T., & Hamilton, C. M. (2010). PhenX: A toolkit for interdisciplinary genetics research. *Current Opinion in Lipidology*, *21*(2), 136–140. https://doi.org/10.1097/MOL.0b013e3283377395](https://www.zotero.org/google-docs/?SJrRhI)

[Strassnig, M., Brar, J. S., & Ganguli, R. (2003). Body mass index and quality of life in community-dwelling patients with schizophrenia. *Schizophrenia Research*, *62*(1–2). https://doi.org/10.1016/S0920-9964(02)00441-3](https://www.zotero.org/google-docs/?SJrRhI)

[Strassnig, M. T., Raykov, T., O’Gorman, C., Bowie, C. R., Sabbag, S., Durand, D., Patterson, T. L., Pinkham, A., Penn, D. L., & Harvey, P. D. (2015). Determinants of different aspects of everyday outcome in schizophrenia: The roles of negative symptoms, cognition, and functional capacity. *Schizophrenia Research*, *165*(1), 76–82. https://doi.org/10.1016/j.schres.2015.03.033](https://www.zotero.org/google-docs/?SJrRhI)

[Strauss, G. P., Harrow, M., Grossman, L. S., & Rosen, C. (2010). Periods of recovery in deficit syndrome schizophrenia: A 20-year multi-follow-up longitudinal study. *Schizophrenia Bulletin*, *36*(4), 788–799. https://doi.org/10.1093/schbul/sbn167](https://www.zotero.org/google-docs/?SJrRhI)

[Stroup, T. S., Lieberman, J. A., McEvoy, J. P., Davis, S. M., Swartz, M. S., Keefe, R. S., Miller, A. L., Rosenheck, R. A., Hsiao, J. K., & Catie Investigators. (2009). Results of phase 3 of the CATIE schizophrenia trial. *Schizophr Res*, *107*(1), 1–12. https://doi.org/10.1016/j.schres.2008.10.011](https://www.zotero.org/google-docs/?SJrRhI)

[Stroup, T. Scott, Gerhard, T., Crystal, S., Huang, C., & Olfson, M. (2015). Comparative Effectiveness of Clozapine and Standard Antipsychotic Treatment in Adults With Schizophrenia. *American Journal of Psychiatry*, *173*(2), 166–173. https://doi.org/10.1176/appi.ajp.2015.15030332](https://www.zotero.org/google-docs/?SJrRhI)

[Stubbs, B., Koyanagi, A., Veronese, N., Vancampfort, D., Solmi, M., Gaughran, F., Carvalho, A. F., Lally, J., Mitchell, A. J., Mugisha, J., & Correll, C. U. (2016). Physical multimorbidity and psychosis: Comprehensive cross sectional analysis including 242,952 people across 48 low- and middle-income countries. *BMC Medicine*, *14*(1). https://doi.org/10.1186/s12916-016-0734-z](https://www.zotero.org/google-docs/?SJrRhI)

[Su, C.-T., Ng, H.-S., Yang, A.-L., & Lin, C.-Y. (2014). Psychometric evaluation of the Short Form 36 Health Survey (SF-36) and the World Health Organization Quality of Life Scale Brief Version (WHOQOL-BREF) for patients with schizophrenia. *Psychological Assessment*, *26*(3), 980–989. https://doi.org/10.1037/a0036764](https://www.zotero.org/google-docs/?SJrRhI)

[Suijkerbuijk, Y. B., Schaafsma, F. G., van Mechelen, J. C., Ojajärvi, A., Corbière, M., & Anema, J. R. (2017). Interventions for obtaining and maintaining employment in adults with severe mental illness, a network meta-analysis. *The Cochrane Database of Systematic Reviews*, *9*, CD011867. https://doi.org/10.1002/14651858.CD011867.pub2](https://www.zotero.org/google-docs/?SJrRhI)

[Sullivan, P. F., Kendler, K. S., & Neale, M. C. (2003). Schizophrenia as a complex trait: Evidence from a meta-analysis of twin studies. *Arch Gen Psychiatry*, *60*(12), 1187–1192. https://doi.org/10.1001/archpsyc.60.12.1187](https://www.zotero.org/google-docs/?SJrRhI)

[Sutin, A. R., Stephan, Y., & Terracciano, A. (2015). Weight Discrimination and Risk of Mortality. *Psychological Science*, *26*(11). https://doi.org/10.1177/0956797615601103](https://www.zotero.org/google-docs/?SJrRhI)

[Swartz, M. S., Perkins, D. O., Stroup, T. S., Davis, S. M., Capuano, G., Rosenheck, R. A., Reimherr, F., McGee, M. F., Keefe, R. S. E., McEvoy, J. P., Hsiao, J. K., Lieberman, J. A., & CATIE Investigators. (2007). Effects of antipsychotic medications on psychosocial functioning in patients with chronic schizophrenia: Findings from the NIMH CATIE study. *The American Journal of Psychiatry*, *164*(3), 428–436. https://doi.org/10.1176/appi.ajp.164.3.428](https://www.zotero.org/google-docs/?SJrRhI)

[Takeuchi, H., Kantor, N., Sanches, M., Fervaha, G., Agid, O., & Remington, G. (2017). One-year symptom trajectories in patients with stable schizophrenia maintained on antipsychotics versus placebo: Meta-analysis. *The British Journal of Psychiatry*, *211*(3), 137–143. https://doi.org/10.1192/bjp.bp.116.186007](https://www.zotero.org/google-docs/?SJrRhI)

[Talbot, K., Eidem, W. L., Tinsley, C. L., Benson, M. A., Thompson, E. W., Smith, R. J., Hahn, C. G., Siegel, S. J., Trojanowski, J. Q., Gur, R. E., Blake, D. J., & Arnold, S. E. (2004). Dysbindin-1 is reduced in intrinsic, glutamatergic terminals of the hippocampal formation in schizophrenia. *J Clin Invest*, *113*(9), 1353–1363. https://doi.org/10.1172/JCI20425](https://www.zotero.org/google-docs/?SJrRhI)

[Tarcijonas, G., Foran, W., Haas, G. L., Luna, B., & Sarpal, D. K. (2020). Intrinsic Connectivity of the Globus Pallidus: An Uncharted Marker of Functional Prognosis in People With First-Episode Schizophrenia. *Schizophrenia Bulletin*, *46*(1), 184–192. https://doi.org/10.1093/schbul/sbz034](https://www.zotero.org/google-docs/?SJrRhI)

[Temesgen, W. A., Chien, W. T., Valimaki, M. A., & Bressington, D. (2020). Predictors of subjective recovery from recent-onset psychosis in a developing country: A mixed-methods study. *Social Psychiatry and Psychiatric Epidemiology*, *55*(9), 1187–1199. https://doi.org/10.1007/s00127-020-01853-5](https://www.zotero.org/google-docs/?SJrRhI)

[Tempelaar, W. M., Termorshuizen, F., MacCabe, J. H., Boks, M. P. M., & Kahn, R. S. (2017). Educational achievement in psychiatric patients and their siblings: A register-based study in 30 000 individuals in The Netherlands. *Psychological Medicine*, *47*(4), 776–784. https://doi.org/10.1017/S0033291716002877](https://www.zotero.org/google-docs/?SJrRhI)

[Teo, C., Zai, C., Borlido, C., Tomasetti, C., Strauss, J., Shinkai, T., Le Foll, B., Wong, A., Kennedy, J. L., & De Luca, V. (2012). Analysis of treatment-resistant schizophrenia and 384 markers from candidate genes. *Pharmacogenet Genomics*, *22*(11), 807–811. https://doi.org/10.1097/FPC.0b013e3283586c04](https://www.zotero.org/google-docs/?SJrRhI)

[Tharyan, P., & Adams, C. E. (2005). Electroconvulsive therapy for schizophrenia. *The Cochrane Database of Systematic Reviews*, *2*, CD000076. https://doi.org/10.1002/14651858.CD000076.pub2](https://www.zotero.org/google-docs/?SJrRhI)

[The Access Point. (2018). *Seeking Supportive Housing: Characteristics, Needs and Outcomes of Applicants to The Access Point*. https://www.wellesleyinstitute.com/wp-content/uploads/2018/04/The-Access-Point-Waiting-List-Analysis-March-2018.pdf](https://www.zotero.org/google-docs/?SJrRhI)

[Thomas, E. C., Despeaux, K. E., Drapalski, A. L., & Bennett, M. (2017). Person-Oriented Recovery of Individuals With Serious Mental Illnesses: A Review and Meta-Analysis of Longitudinal Findings. *Psychiatric Services*, *69*(3), 259–267. https://doi.org/10.1176/appi.ps.201700058](https://www.zotero.org/google-docs/?SJrRhI)

[Tiihonen, J., Tanskanen, A., & Taipale, H. (2018). 20-Year Nationwide Follow-Up Study on Discontinuation of Antipsychotic Treatment in First-Episode Schizophrenia. *American Journal of Psychiatry*, *175*(8), 765–773. https://doi.org/10.1176/appi.ajp.2018.17091001](https://www.zotero.org/google-docs/?SJrRhI)

[Tolman, A. W., & Kurtz, M. M. (2012). Neurocognitive predictors of objective and subjective quality of life in individuals with schizophrenia: A meta-analytic investigation. *Schizophrenia Bulletin*, *38*(2), 304–315. https://doi.org/10.1093/schbul/sbq077](https://www.zotero.org/google-docs/?SJrRhI)

[Tomiyama, A. J., Carr, D., Granberg, E. M., Major, B., Robinson, E., Sutin, A. R., & Brewis, A. (2018). How and why weight stigma drives the obesity “epidemic” and harms health. *BMC Med*, *16*(1), 123. https://doi.org/10.1186/s12916-018-1116-5](https://www.zotero.org/google-docs/?SJrRhI)

[Torous, J., Woodyatt, J., Keshavan, M., & Tully, L. M. (2019). A new hope for early psychosis care: The evolving landscape of digital care tools. *Br J Psychiatry*, *214*(5), 269–272. https://doi.org/10.1192/bjp.2019.8](https://www.zotero.org/google-docs/?SJrRhI)

[Tsemberis, S., McHugo, G., Williams, V., Hanrahan, P., & Stefancic, A. (2007). Measuring homelessness and residential stability: The residential time-line follow-back inventory. *Journal of Community Psychology*, *35*(1), 29–42. https://doi.org/10.1002/jcop.20132](https://www.zotero.org/google-docs/?SJrRhI)

[Turner, D. T., van der Gaag, M., Karyotaki, E., & Cuijpers, P. (2014). Psychological interventions for psychosis: A meta-analysis of comparative outcome studies. *Am J Psychiatry*, *171*(5), 523–538. https://doi.org/10.1176/appi.ajp.2013.13081159](https://www.zotero.org/google-docs/?SJrRhI)

[Üçok, A., & Ergül, C. (2014). Persistent negative symptoms after first episode schizophrenia: A 2-year follow-up study. *Schizophrenia Research*, *158*(1), 241–246. https://doi.org/10.1016/j.schres.2014.07.021](https://www.zotero.org/google-docs/?SJrRhI)

[Ustun, T. B., Chatterji, S., Kostanjsek, N., Rehm, J., Kennedy, C., Epping-Jordan, J., Saxena, S., von Korff, M., Pull, C., & Who Nih Joint Project. (2010). Developing the World Health Organization Disability Assessment Schedule 2.0. *Bull World Health Organ*, *88*(11), 815–823. https://doi.org/10.2471/BLT.09.067231](https://www.zotero.org/google-docs/?SJrRhI)

[Valaparla, V. L., Nehra, R., Mehta, U. M., Thirthalli, J., & Grover, S. (2017). Social cognition of patients with schizophrenia across the phases of illness—A longitudinal study. *Schizophrenia Research*, *190*, 150–159. https://doi.org/10.1016/j.schres.2017.03.008](https://www.zotero.org/google-docs/?SJrRhI)

[Van der Does, A. J., Dingemans, P. M., Linszen, D. H., Nugter, M. A., & Scholte, W. F. (1993). Symptom dimensions and cognitive and social functioning in recent-onset schizophrenia. *Psychological Medicine*, *23*(3), 745–753. https://doi.org/10.1017/s0033291700025514](https://www.zotero.org/google-docs/?SJrRhI)

[Van Eck, R. M., Burger, T. J., Vellinga, A., Schirmbeck, F., & de Haan, L. (2018). The Relationship Between Clinical and Personal Recovery in Patients With Schizophrenia Spectrum Disorders: A Systematic Review and Meta-analysis. *Schizophrenia Bulletin*, *44*(3), 631–642. https://doi.org/10.1093/schbul/sbx088](https://www.zotero.org/google-docs/?SJrRhI)

[van Os, J., & Kapur, S. (2009). Schizophrenia. *Lancet*, *374*(9690), 635–645. https://doi.org/10.1016/S0140-6736(09)60995-8](https://www.zotero.org/google-docs/?SJrRhI)

[Vancampfort, D., Probst, M., Scheewe, T., Knapen, J., De Herdt, A., & De Hert, M. (2012). The functional exercise capacity is correlated with global functioning in patients with schizophrenia. *Acta Psychiatrica Scandinavica*, *125*(5). https://doi.org/10.1111/j.1600-0447.2011.01825.x](https://www.zotero.org/google-docs/?SJrRhI)

[Vassos, E., Pedersen, C. B., Murray, R. M., Collier, D. A., & Lewis, C. M. (2012). Meta-analysis of the association of urbanicity with schizophrenia. *Schizophrenia Bulletin*, *38*(6), 1118–1123. https://doi.org/10.1093/schbul/sbs096](https://www.zotero.org/google-docs/?SJrRhI)

[Velligan, D. I., Bow-Thomas, C. C., Huntzinger, C., Ritch, J., Ledbetter, N., Prihoda, T. J., & Miller, A. L. (2000). Randomized controlled trial of the use of compensatory strategies to enhance adaptive functioning in outpatients with schizophrenia. *The American Journal of Psychiatry*, *157*(8), 1317–1323. https://doi.org/10.1176/appi.ajp.157.8.1317](https://www.zotero.org/google-docs/?SJrRhI)

[Velligan, D. I., Diamond, P. M., Maples, N. J., Mintz, J., Li, X., Glahn, D. C., & Miller, A. L. (2008). Comparing the efficacy of interventions that use environmental supports to improve outcomes in patients with schizophrenia. *Schizophrenia Research*, *102*(1–3), 312–319. https://doi.org/10.1016/j.schres.2008.02.005](https://www.zotero.org/google-docs/?SJrRhI)

[Velligan, D. I., Mahurin, R. K., Diamond, P. L., Hazleton, B. C., Eckert, S. L., & Miller, A. L. (1997). The functional significance of symptomatology and cognitive function in schizophrenia. *Schizophrenia Research*, *25*(1), 21–31. https://doi.org/10.1016/S0920-9964(97)00010-8](https://www.zotero.org/google-docs/?SJrRhI)

[Velligan, D. I., Prihoda, T. J., Ritch, J. L., Maples, N., Bow-Thomas, C. C., & Dassori, A. (2002). A randomized single-blind pilot study of compensatory strategies in schizophrenia outpatients. *Schizophrenia Bulletin*, *28*(2), 283–292. https://doi.org/10.1093/oxfordjournals.schbul.a006938](https://www.zotero.org/google-docs/?SJrRhI)

[Velthorst, E., Koeter, M., der Gaag, M. van, Nieman, D. H., Fett, A.-K. J., Smit, F., Staring, A. B. P., Meijer, C., & de Haan, L. (2014). Adapted cognitive–behavioural therapy required for targeting negative symptoms in schizophrenia: Meta-analysis and meta-regression. *Psychological Medicine*, 1–13. https://doi.org/10.1017/S0033291714001147](https://www.zotero.org/google-docs/?SJrRhI)

[Velthorst, Eva, Fett, A.-K. J., Reichenberg, A., Perlman, G., van Os, J., Bromet, E. J., & Kotov, R. (2017). The 20-Year Longitudinal Trajectories of Social Functioning in Individuals With Psychotic Disorders. *The American Journal of Psychiatry*, *174*(11), 1075–1085. https://doi.org/10.1176/appi.ajp.2016.15111419](https://www.zotero.org/google-docs/?SJrRhI)

[Ventura, J., Ered, A., Gretchen-Doorly, D., Subotnik, K. L., Horan, W. P., Hellemann, G. S., & Nuechterlein, K. H. (2015). Theory of mind in the early course of schizophrenia: Stability, symptom and neurocognitive correlates, and relationship with functioning. *Psychological Medicine*, *45*(10), 2031–2043. https://doi.org/10.1017/S0033291714003171](https://www.zotero.org/google-docs/?SJrRhI)

[Ventura, J., Lukoff, D., Nuechterlein, K. H., Liberman, R. P., Green, M. F., & Shaner, A. (1993). Appendix 1: Brief Psychiatric Rating Scale ŽBPRS. Expanded Version Ž4.0. Scales, anchor points and administration manual. *International Journal of Methods in Psychiatric Research*, *3*, 227–243.](https://www.zotero.org/google-docs/?SJrRhI)

[Ventura, J., Subotnik, K. L., Gitlin, M. J., Gretchen-Doorly, D., Ered, A., Villa, K. F., Hellemann, G. S., & Nuechterlein, K. H. (2015). Negative symptoms and functioning during the first year after a recent onset of schizophrenia and 8 years later. *Schizophrenia Research*, *161*(2–3), 407–413. https://doi.org/10.1016/j.schres.2014.10.043](https://www.zotero.org/google-docs/?SJrRhI)

[Ventura, Joseph, Hellemann, G. S., Thames, A. D., Koellner, V., & Nuechterlein, K. H. (2009). Symptoms as mediators of the relationship between neurocognition and functional outcome in schizophrenia: A meta-analysis. *Schizophrenia Research*, *113*(2–3), 189–199. https://doi.org/10.1016/j.schres.2009.03.035](https://www.zotero.org/google-docs/?SJrRhI)

[Vernon, J. A., Grudnikoff, E., Seidman, A. J., Frazier, T. W., Vemulapalli, M. S., Pareek, P., Goldberg, T. E., Kane, J. M., & Correll, C. U. (2014). Antidepressants for cognitive impairment in schizophrenia—A systematic review and meta-analysis. *Schizophrenia Research*, *159*(2–3), 385–394. https://doi.org/10.1016/j.schres.2014.08.015](https://www.zotero.org/google-docs/?SJrRhI)

[Villalta-Gil, V., Vilaplana, M., Ochoa, S., Haro, J. M., Dolz, M., Usall, J., & Cervilla, J. (2006). Neurocognitive performance and negative symptoms: Are they equal in explaining disability in schizophrenia outpatients? *Schizophrenia Research*, *87*(1), 246–253. https://doi.org/10.1016/j.schres.2006.06.013](https://www.zotero.org/google-docs/?SJrRhI)

[Vita, A., Minelli, A., Barlati, S., Deste, G., Giacopuzzi, E., Valsecchi, P., Turrina, C., & Gennarelli, M. (2019). Treatment-Resistant Schizophrenia: Genetic and Neuroimaging Correlates. *Front Pharmacol*, *10*, 402. https://doi.org/10.3389/fphar.2019.00402](https://www.zotero.org/google-docs/?SJrRhI)

[Viviano, J. D., Buchanan, R. W., Calarco, N., Gold, J. M., Foussias, G., Bhagwat, N., Stefanik, L., Hawco, C., DeRosse, P., Argyelan, M., Turner, J., Chavez, S., Kochunov, P., Kingsley, P., Zhou, X., Malhotra, A. K., Voineskos, A. N., & Social Processes Initiative in Neurobiology of the Schizophrenia(s) Group. (2018). Resting-State Connectivity Biomarkers of Cognitive Performance and Social Function in Individuals With Schizophrenia Spectrum Disorder and Healthy Control Subjects. *Biological Psychiatry*, *84*(9), 665–674. https://doi.org/10.1016/j.biopsych.2018.03.013](https://www.zotero.org/google-docs/?SJrRhI)

[Voineskos, A. N., Foussias, G., Lerch, J., Felsky, D., Remington, G., Rajji, T. K., Lobaugh, N., Pollock, B. G., & Mulsant, B. H. (2013). Neuroimaging evidence for the deficit subtype of schizophrenia. *JAMA Psychiatry*, *70*(5), 472–480. https://doi.org/10.1001/jamapsychiatry.2013.786](https://www.zotero.org/google-docs/?SJrRhI)

[Wang, L., Lakin, J., Riley, C., Korach, Z., Frain, L. N., & Zhou, L. (2018). Disease Trajectories and End-of-Life Care for Dementias: Latent Topic Modeling and Trend Analysis Using Clinical Notes. *AMIA Annu Symp Proc*, *2018*, 1056–1065.](https://www.zotero.org/google-docs/?SJrRhI)

[Wang, L., Sha, L., Lakin, J. R., Bynum, J., Bates, D. W., Hong, P., & Zhou, L. (2019). Development and Validation of a Deep Learning Algorithm for Mortality Prediction in Selecting Patients With Dementia for Earlier Palliative Care Interventions. *JAMA Netw Open*, *2*(7), e196972. https://doi.org/10.1001/jamanetworkopen.2019.6972](https://www.zotero.org/google-docs/?SJrRhI)

[Weiden, P. J., Kozma, C., Grogg, A., & Locklear, J. (2004). Partial compliance and risk of rehospitalization among California Medicaid patients with schizophrenia. *Psychiatr Serv*, *55*(8), 886–891. https://doi.org/10.1176/appi.ps.55.8.886](https://www.zotero.org/google-docs/?SJrRhI)

[Weinberger, D. R. (2019). Thinking About Schizophrenia in an Era of Genomic Medicine. *American Journal of Psychiatry*, *176*(1), 12–20. https://doi.org/10.1176/appi.ajp.2018.18111275](https://www.zotero.org/google-docs/?SJrRhI)

[Werner, M. C. F., Wirgenes, K. V., Haram, M., Bettella, F., Lunding, S. H., Rodevand, L., Hjell, G., Agartz, I., Djurovic, S., Melle, I., Andreassen, O. A., & Steen, N. E. (2020). Indicated association between polygenic risk score and treatment-resistance in a naturalistic sample of patients with schizophrenia spectrum disorders. *Schizophr Res*, *218*, 55–62. https://doi.org/10.1016/j.schres.2020.03.006](https://www.zotero.org/google-docs/?SJrRhI)

[Westcott, C., Waghorn, G., McLean, D., Statham, D., & Mowry, B. (2015). Interest in Employment Among People with Schizophrenia. *American Journal of Psychiatric Rehabilitation*, *18*(2), 187–207. https://doi.org/10.1080/15487768.2014.954162](https://www.zotero.org/google-docs/?SJrRhI)

[Wharton, S., Lau, D. C. W., Vallis, M., Sharma, A. M., Biertho, L., Campbell-Scherer, D., Adamo, K., Alberga, A., Bell, R., Boulé, N., Boyling, E., Brown, J., Calam, B., Clarke, C., Crowshoe, L., Divalentino, D., Forhan, M., Freedhoff, Y., Gagner, M., … Wicklum, S. (2020). Obesity in adults: A clinical practice guideline. *CMAJ*, *192*(31). https://doi.org/10.1503/cmaj.191707](https://www.zotero.org/google-docs/?SJrRhI)

[Whiteford, H. A., Ferrari, A. J., Degenhardt, L., Feigin, V., & Vos, T. (2015). The global burden of mental, neurological and substance use disorders: An analysis from the Global Burden of Disease Study 2010. *PLoS ONE*, *10*(2), e0116820. https://doi.org/10.1371/journal.pone.0116820](https://www.zotero.org/google-docs/?SJrRhI)

[Whitley, R., Shepherd, G., & Slade, M. (2019). Recovery colleges as a mental health innovation. *World Psychiatry*, *18*(2), 141–142. https://doi.org/10.1002/wps.20620](https://www.zotero.org/google-docs/?SJrRhI)

[Whitley, R., Sitter, K. C., Adamson, G., & Carmichael, V. (2020). A meaningful focus: Investigating the impact of involvement in a participatory video program on the recovery of participants with severe mental illness. *Psychiatric Rehabilitation Journal*. https://doi.org/10.1037/prj0000416](https://www.zotero.org/google-docs/?SJrRhI)

[Wiersma, D., Wanderling, J., Dragomirecka, E., Ganev, K., Harrison, G., an der Heiden, W., Nienhuis, F. J., & Walsh, D. (2000). Social disability in schizophrenia: Its development and prediction over 15 years in incidence cohorts in six European centres. *Psychological Medicine*, *30*(5), 1155–1167.](https://www.zotero.org/google-docs/?SJrRhI)

[Willette, A. A., & Kapogiannis, D. (2015). *Does the brain shrink as the waist expands?*](https://www.zotero.org/google-docs/?SJrRhI)

[Williams, D. R., Yan Yu, null, Jackson, J. S., & Anderson, N. B. (1997). Racial Differences in Physical and Mental Health: Socio-economic Status, Stress and Discrimination. *Journal of Health Psychology*, *2*(3), 335–351. https://doi.org/10.1177/135910539700200305](https://www.zotero.org/google-docs/?SJrRhI)

[Wodchis, W. P., Austin, P. C., & Henry, D. A. (2016). A 3-year study of high-cost users of health care. *CMAJ*, *188*(3), 182–188. https://doi.org/10.1503/cmaj.150064](https://www.zotero.org/google-docs/?SJrRhI)

[Wodchis, W. P., Bushmeneva, K., Nikitovic, M., & McKillop, I. (2013). *Guidelines on Person-Level Costing Using Administrative Databases in Ontario*. 71.](https://www.zotero.org/google-docs/?SJrRhI)

[World Health Organization. (1996). *WHOQOL-BREF : introduction, administration, scoring and generic version of the assessment: Field trial version, December 1996*. *WHOQOL-BREF*. WHO IRIS. https://apps.who.int/iris/handle/10665/63529](https://www.zotero.org/google-docs/?SJrRhI)

[Wray, R., Agic, B., Bennett-AbuAyyash, C., Kanee, M., Tuck, A., Lam, R., Mohamed, A., & Hyman, I. (2013). *We ask because we care: The Tri-Hospital + TPH health equity data collection research project: Full Report.*](https://www.zotero.org/google-docs/?SJrRhI)

[Yanos, P. T., Roe, D., Markus, K., & Lysaker, P. H. (2008). Pathways Between Internalized Stigma and Outcomes Related to Recovery in Schizophrenia Spectrum Disorders. *Psychiatric Services*, *59*(12), 1437–1442. https://doi.org/10.1176/ps.2008.59.12.1437](https://www.zotero.org/google-docs/?SJrRhI)

[Young, D. K., Cheng, D., & Ng, P. (2020). Predictors of Personal Recovery of People with Severe Mental Illness in a Chinese Society: A Cross-Sectional Study with a Random Sample. *International Journal of Mental Health and Addiction*, *18*(4), 1168–1179. https://doi.org/10.1007/s11469-019-00134-w](https://www.zotero.org/google-docs/?SJrRhI)

[Young, S. J., Praskova, A., Hayward, N., & Patterson, S. (2017). Attending to physical health in mental health services in Australia: A qualitative study of service users’ experiences and expectations. *Health & Social Care in the Community*, *25*(2), 602–611.](https://www.zotero.org/google-docs/?SJrRhI)

[Yu, A. P., Atanasov, P., Ben-Hamadi, R., Birnbaum, H., Stensland, M. D., & Philips, G. (2009). Resource utilization and costs of schizophrenia patients treated with olanzapine versus quetiapine in a Medicaid population. *Value in Health: The Journal of the International Society for Pharmacoeconomics and Outcomes Research*, *12*(5), 708–715. https://doi.org/10.1111/j.1524-4733.2008.00498.x](https://www.zotero.org/google-docs/?SJrRhI)

[Yu, H., Shimakawa, A., Hines, C. D. G., McKenzie, C. A., Hamilton, G., Sirlin, C. B., Brittain, J. H., & Reeder, S. B. (2011). Combination of complex-based and magnitude-based multiecho water-fat separation for accurate quantification of fat-fraction. *Magnetic Resonance in Medicine*, *66*(1), 199–206. https://doi.org/10.1002/mrm.22840](https://www.zotero.org/google-docs/?SJrRhI)

[Yu, H., Shimakawa, A., McKenzie, C. A., Brodsky, E., Brittain, J. H., & Anderson, S. B. (2008). Multiecho water-fat separation and simultaneous R2* estimation with multifrequency fat spectrum modeling. *Magnetic Resonance in Medicine*, *60*(5), 1122–1134. https://doi.org/10.1002/mrm.21737](https://www.zotero.org/google-docs/?SJrRhI)

[Zai, C. C., Goncalves, V. F., Tiwari, A. K., Gagliano, S. A., Hosang, G., de Luca, V., Shaikh, S. A., King, N., Chen, Q., Xu, W., Strauss, J., Breen, G., Lewis, C. M., Farmer, A. E., McGuffin, P., Knight, J., Vincent, J. B., & Kennedy, J. L. (2015). A genome-wide association study of suicide severity scores in bipolar disorder. *J Psychiatr Res*, *65*, 23–29. https://doi.org/10.1016/j.jpsychires.2014.11.002](https://www.zotero.org/google-docs/?SJrRhI)

[Zai, C., Fabbri, C., Hosang, G., Zhang, R., Koyama, E., de Luca, V., Tiwari, A., King, N., Strauss, J., Jones, I., Jones, L., Breen, G., Farmer, A., McGuffin, P., Vincent, J., Kennedy, J., & Lewis, C. (n.d.). Genome-wide association study of suicidal behaviour severity in mood disorders. *World Journal of Biological Psychiatry*.](https://www.zotero.org/google-docs/?SJrRhI)

[Zhao, X., Shi, Y., Tang, J., Tang, R., Yu, L., Gu, N., Feng, G., Zhu, S., Liu, H., Xing, Y., Zhao, S., Sang, H., Guan, Y., St Clair, D., & He, L. (2004). A case control and family based association study of the neuregulin1 gene and schizophrenia. *J Med Genet*, *41*(1), 31–34. https://doi.org/10.1136/jmg.2003.014977](https://www.zotero.org/google-docs/?SJrRhI)

[Zhou, L., Baughman, A. W., Lei, V. J., Lai, K. H., Navathe, A. S., Chang, F., Sordo, M., Topaz, M., Zhong, F., Murrali, M., Navathe, S., & Rocha, R. A. (2015). Identifying Patients with Depression Using Free-text Clinical Documents. *Stud Health Technol Inform*, *216*, 629–633.](https://www.zotero.org/google-docs/?SJrRhI)

[Zimet, G. D., Powell, S. S., Farley, G. K., Werkman, S., & Berkoff, K. A. (1990). Psychometric Characteristics of the Multidimensional Scale of Perceived Social Support. *Journal of Personality Assessment*, *55*(3–4), 610–617. https://doi.org/10.1080/00223891.1990.9674095](https://www.zotero.org/google-docs/?SJrRhI)

[Zimmermann, G., Favrod, J., Trieu, V. H., & Pomini, V. (2005). The effect of cognitive behavioral treatment on the positive symptoms of schizophrenia spectrum disorders: A meta-analysis. *Schizophr Res*, *77*(1), 1–9. https://doi.org/10.1016/j.schres.2005.02.018](https://www.zotero.org/google-docs/?SJrRhI)
